# Sequential and directional insulation by conserved CTCF sites underlies the Hox timer in stembryos

**DOI:** 10.1038/s41588-023-01426-7

**Published:** 2023-06-15

**Authors:** Hocine Rekaik, Lucille Lopez-Delisle, Aurélie Hintermann, Bénédicte Mascrez, Célia Bochaton, Alexandre Mayran, Denis Duboule

**Affiliations:** 1 School of Life Sciences, École Polytechnique Fédérale de Lausanne (EPFL), Lausanne, Switzerland; 2 Department of Genetics and Evolution, University of Geneva, Geneva, Switzerland; 3 Collège de France, Paris, France

**Keywords:** Embryogenesis, Epigenetics, Gene regulation

## Abstract

During development, *Hox* genes are temporally activated according to their relative positions on their clusters, contributing to the proper identities of structures along the rostrocaudal axis. To understand the mechanism underlying this Hox timer, we used mouse embryonic stem cell-derived stembryos. Following *Wnt* signaling, the process involves transcriptional initiation at the anterior part of the cluster and a concomitant loading of cohesin complexes enriched on the transcribed DNA segments, that is, with an asymmetric distribution favoring the anterior part of the cluster. Chromatin extrusion then occurs with successively more posterior CTCF sites acting as transient insulators, thus generating a progressive time delay in the activation of more posterior-located genes due to long-range contacts with a flanking topologically associating domain. Mutant stembryos support this model and reveal that the presence of evolutionary conserved and regularly spaced intergenic CTCF sites controls the precision and the pace of this temporal mechanism.

## Abstract (web summary)

Temporal activation of *Hox* genes in mouse pseudo-embryos in vitro initiates at the anterior part of the cluster and is accompanied by asymmetric loading of cohesin. Posterior CTCF sites then successively act as transient insulators, regulating the timed transcription of more posterior-located genes.

## Main

In mammals, *Hox* genes are transcribed during gastrulation, when the embryo produces and organizes its major body axis^1^. By the end of gastrulation, the embryo displays the classical distribution of *Hox* mRNAs, with progressively overlapping domains. Consequently, cells at various anterior–posterior (AP) body levels express distinct combinations of HOX proteins, which may genetically instruct cellular populations as to which morphologies they should produce^2,3^. The spatial activation of any *Hox* gene is largely fixed by its relative position within its genomic cluster^4^, an unusual property described in flies^5,6^ and in most animals with an AP axis^7–9^. In vertebrates, this mechanism is linked to a time sequence in transcriptional activation, initially observed in mammals^10,11^ and subsequently generalized^12,13^. Although the function of this timer has been discussed before^14–16^, its mechanism has remained poorly characterized due to the difficulties of analyzing the few neuro-mesodermal progenitor cells that feed the elongating axis with new mesoderm and neurectoderm tissue^17,18^ and where most *Hox* genes are activated during axial extension.

A model was proposed whereby a progressive and directional opening of a closed chromatin configuration would parallel a stepwise accessibility of neighboring genes to activating factors^19,20^, with the onset of activation depending on *Wnt* signaling^21^, a signaling pathway active at the most posterior part of the developing embryo^22^. In subsequent phases, Cdx transcription factors were reported to activate more centrally located *Hox* genes^23–25^, while *Gdf11* signaling might regulate more 5′-located (posterior) genes^26,27^. Coincidentally, the mapping of CTCF-binding sites (CBSs) within *Hox* clusters revealed the following three sub-domains: an ‘anterior’ domain devoid of CTCF sites, a centrally located domain where a series of CTCF sites are orientated toward the 3′ end of the clusters and a posterior domain where several CTCF sites display the opposite orientation^28^. This organization of CTCF sites is highly conserved either between species^29^ or between paralogous gene clusters^28^, that is, over several hundred million years of evolution, raising the hypothesis that they may act as checkpoints in the temporal activation of interspersed *Hox* genes, due to their involvement in the making and stabilization of large loops along with the cohesin complex^30,31^.

Here we revisit this question by using gastruloids^32^ derived from aggregated mouse embryonic stem (mES) cells cultivated in vitro for several days. After activating *Wnt* signaling for 24 h, such ‘stembryos’^33^ start to elongate a protrusion that resembles the outgrowth of the tail bud^34^. We show that the *Hox* timer starts with a *Wnt*-dependent transcription of the CTCF-free part of the cluster, which triggers an increased asymmetric loading of cohesin complexes over this domain. This is rapidly followed by the stepwise transcriptional activation of genes in the CTCF-rich region after a 3′-to-5′ progression in loop-extrusion, along with progressive changes in the chromatin architecture of the locus. We challenged this model by using mutant stembryos and further showed that, while the first phase is sufficient to introduce a 5′-to-3′ asymmetry in transcription, CTCF sites organize and secure the sequence and the pace of this timer.

## Results

### Timecourse of *Hox* gene activation in stembryos

**Fig. 1:**
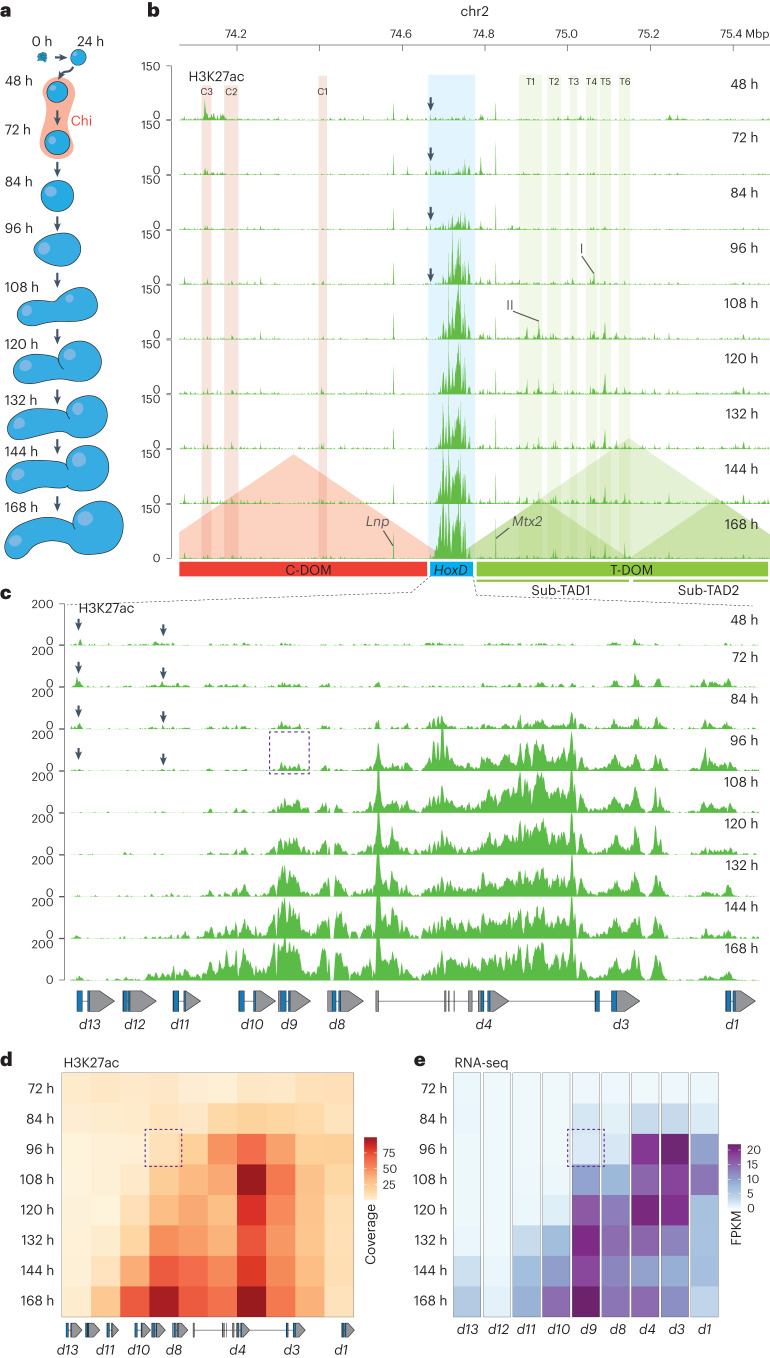
Sequential activation of the *HoxD* gene cluster upon *Wnt* signaling. **a**, Various stembryonic stages were used for the timecourse with the pulse of *Wnt* agonist in red. **b**, Timecourse of H3K27ac ChIP–seq over the entire *HoxD* locus. Genomic coordinates are on top, and the positions of C-DOM and T-DOM are shown in red and green, respectively, at the bottom. The quantification of the H3K27ac signals within the colored vertical columns is in Extended Data Fig. 1. Acetylation peaks at the *Mtx* and *Lnp* promoters are indicated. I and II point to the acetylation peaks used in Extended Data Fig. 1. **c**, Magnification of the *HoxD* cluster showing the progressive anterior to posterior spreading of H3K27ac. The start of robust coverage in the anterior part is counterbalanced by a decrease in the weaker acetylation initially detected in posterior regions (black arrows in **b** and **c**). **d**, Heatmap of H3K27ac ChIP–seq coverage over the *HoxD* cluster at various timepoints. Squares represent 10-kb DNA segments. **e**, Heatmap of FPKM values of *Hoxd* genes transcripts over time (average of two replicates). The dashed region around *Hoxd9* at 96 h in **c**–**e** highlights the increased activation in all subsequent stages when compared to *Hoxd4*. Genomic coordinates for **c** and **d**—chr2:74667374–74767842.

In gastrulating mouse embryos, *Wnt* signaling contributes to the formation of the primitive streak from epiblast cells. Likewise, in stembryos cultured as described in ref. ^34^, a pulse of the *Wnt* agonist Chiron 48 h after aggregation of mES cells, that is, between 48 h and 72 h, triggers the differentiation of these epiblast-like cells to form a multilayered structure resembling a posterior elongating body axis. We generated a 12-h timecourse chromatin immunoprecipitation followed by sequencing (ChIP–seq) dataset for H3K27ac, a chromatin mark found at active enhancers and associated with transcription (Fig. 1a–c), and analyzed the various H3K27ac profiles over the *HoxD* cluster and its flanking centromeric and telomeric regulatory domains (C-DOM and T-DOM, respectively), two topologically associating domains (TADs) with the latter being split into sub-TADs 1 and 2 containing enhancers of various specificities (for example, ref. ^35^).

**Extended Data Fig. 1:**
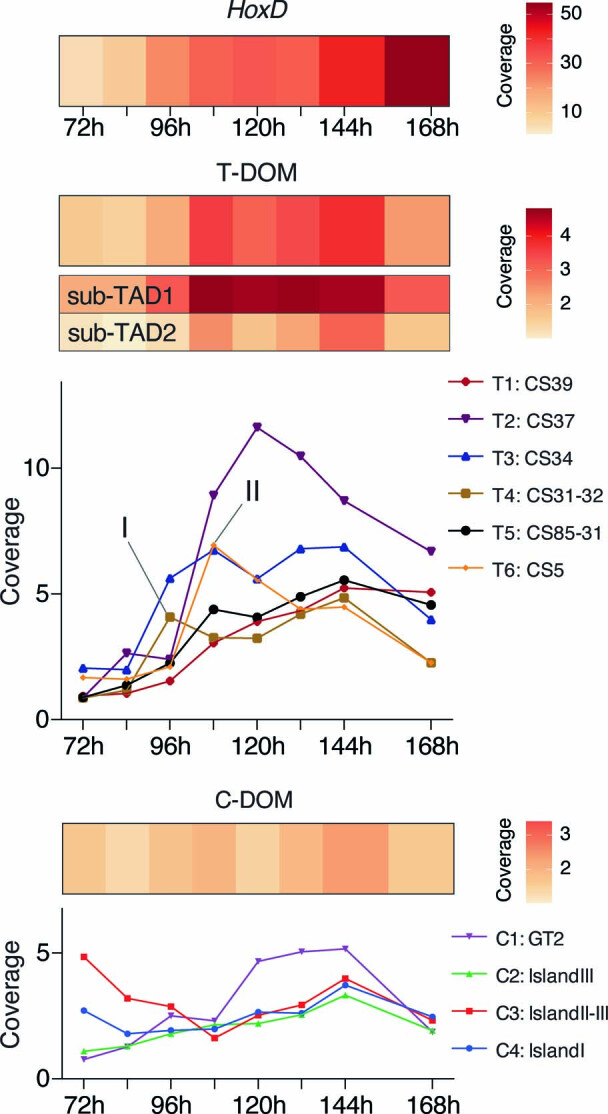
Quantification of the H3K27ac ChIP-seq coverage over the *HoxD* cluster (top), the T-DOM TAD (middle) and the C-DOM TAD (bottom). Each square represents the average coverage value for a given time-point. The increase in H3K27ac coverage over the *HoxD* cluster and T-DOM (with a strong preference for sub-TAD1) contrasts with C-DOM (bottom). Graphs showing quantifications over time of the colored vertical segments illustrated in Fig. 1b (red for C-DOM and green for T-DOM). Each line represents a specific H3K27 acetylated element within the *HoxD* regulatory landscape. The time sequence in the activation of these elements in T-DOM does not correlate with their distance from the genes cluster (I and II).

Soon after Chiron treatment (72 h), H3K27ac peaks appeared in the ‘anterior’ part of the cluster (Fig. 1c). From 72 h to 84 h, signals covered mostly *Hoxd1, Hoxd3* and *Hoxd4*, whereas no signal was detected in T-DOM. Acetylation signals appeared there at 96 h and became stronger in sub-TAD1 along with the increased acetylation of the *HoxD* cluster (Fig. 1b, 96 h, green columns). The appearance of these peaks matched the time window of transcriptional activation of the cluster (Extended Data Fig. 1). In contrast, no significant H3K27ac signal scored in C-DOM (Fig. 1b, pink columns, and Extended Data Fig. 1), showing the asymmetry and the importance of T-DOM in this early activating phase.

After the increase of H3K27ac over the *Hoxd1*–*Hoxd3* region at 72 h, acetylation marks rapidly spread over the whole anterior cluster, up to between *Hoxd4* and *Hoxd8* (Fig. 1c,d, 84 h). From 96 h, more progressive acetylation was observed throughout the rest of the cluster, eventually covering the *Hoxd8*–*Hoxd12* segment at 168 h (Fig. 1c,d). This multiphasic dynamic (Supplementary Movie 1) was corroborated both by transcriptome analysis (Fig. 1e) and by the profiles of bound RNA pol II (Supplementary Note 1 and Supplementary Figs. 1 and 2). Indeed, *Hoxd1* to *Hoxd4* mRNAs were detected at 84 h and became more abundant at 96 h, a stage where *Hoxd8* and *Hoxd9* expression was low. The amounts of mRNAs from the latter two genes only increased at 108 h. Likewise, expression of *Hoxd10* and *Hoxd11* was scored at 132 h and *Hoxd13* at around 144 h, correlating with the acetylation dynamics (Fig. 1d,e).

Of note, a low level of H3K27ac was scored over the entire posterior part of the cluster at 72 h, which was erased after the activation of the cluster at 96 h (Fig. 1b,c, black arrows). Transcripts from these genes were nevertheless not detected at these early timepoints, suggesting that the cluster was still in a bivalent chromatin configuration, as in mES cells^36^. Also, while the initial activation of *Hoxd1*–*Hoxd4* occurred before any T-DOM activity was scored, the progressive activation starting at *Hoxd8* was clearly associated with the acetylation of many sub-TAD1 sequences, suggesting long-range regulation participates to the activation of more central and posterior genes.

### Loading of the cohesin complex in stembryos

**Fig. 2:**
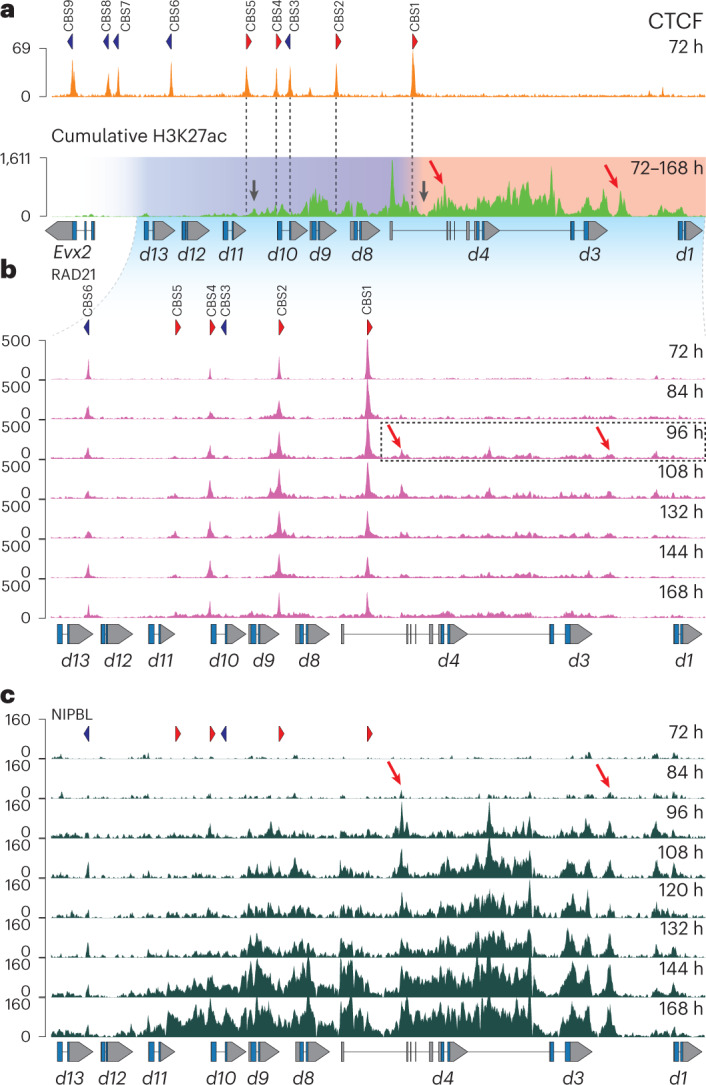
Dynamics and directional cohesin loading at the *HoxD* cluster. **a**, CTCF ChIP–seq profile using stembryos at 72 h (upper panel). The orientations of CBS1-9 motifs are shown with red and blue arrowheads. Below are cumulative H3K27ac ChIP–seq signals from 72 h to 168 h showing the various points of transitions in transcriptional activation over time. The red domain delineates the initial and rapid phase of acetylation, while the following and more progressive activation phase occurs in the blue domain. Black arrows point to regions where the progression in H3K27 acetylation was delayed (Supplementary Movie 1). **b**, Timecourse of RAD21 ChIP–seq profiles. CBS number and orientations are as in **a**. The accumulation of RAD21 outside CBSs is exemplified by the dashed box (quantifications in Extended Data Fig. 2a). **c**, Timecourse of NIPBL ChIP–seq profiles over *Hoxd* genes. NIPBL binding corresponds to acetylated regions. Progressive NIPBL enrichment correlates with the activation dynamics of the cluster. Red arrows in **a**–**c** highlight two acetylated regions where NIPBL is detected and RAD21 accumulates. Genomic coordinates—chr2:74667374–74767842.

In stembryos, we identified nine occupied CBSs inside *HoxD* (Fig. 2a, upper panel). Four of the five ‘anterior’ CBSs displayed an orientation opposite to those CBSs located in T-DOM (Fig. 2a, CBS1, CBS2, CBS4 and CBS5, red), whereas the four CBSs located more ‘posteriorly’ had the reverse orientation (Fig. 2a, CBS6, CBS7, CBS8 and CBS9, blue), identical to the embryonic pattern^28,37^. Because genes in the CTCF-free domain are activated almost concomitantly, we asked whether the subsequent time progression in acetylation depends on the presence of the CBSs. A cumulative profile of H3K27ac signals between 72 h and 168 h showed transitions over time (Fig. 2a, lower panel) with CBSs systematically matching poorly acetylated regions, some of them positioned where the 3′-to-5′ progression in acetylation was slowed down (Fig. 2a, black arrows, and Supplementary Movie 1), suggesting that CBSs may influence the progressive spreading of acetylation.

**Extended Data Fig. 2:**
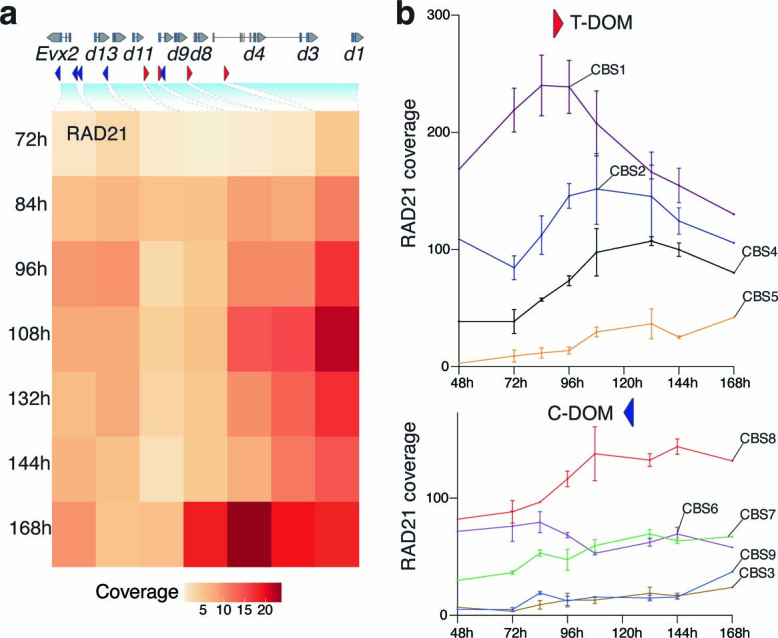
Quantification of RAD21 coverage. **a**, Heatmaps of RAD21 ChIP-seq average coverage over inter-CBS regions at the indicated time-points (n = 1). Each square represents a segment located between two CBS within the *HoxD* cluster (scheme on top). The positions and orientations of CBS are indicated with red and blue arrowheads. An increased RAD21 coverage is detected over the activated region of the gene cluster, when compared to the silent posterior part. **b**, RAD21 ChIP-seq coverage over those CBS located within the cluster and oriented either toward T-DOM (top), or towards C-DOM (bottom). The dynamic of coverage over time (x-axis) indicates a progressive accumulation of extruding cohesin at those CBS oriented toward T-DOM, that is, where active loading was observed (n = 2; 48 and 168 h: n = 1; 96 and 144 h: n = 3). Line is mean ± SD.

We then looked at the presence of cohesin complexes by using RAD21, which was expectedly detected at several CBSs^38^, although with various enrichments (Fig. 2b and Extended Data Fig. 2). Along with stembryonic development, low but significant levels of RAD21 accumulated outside CBSs, within gene bodies (Fig. 2b, dashed box). This latter accumulation spread toward more posterior genes in subsequent stages, along with transcription (Fig. 2b and Extended Data Fig. 2a; compare 96 h with 168 h). To back up this observation, we profiled NIPBL, one of the loading factors of the cohesin complex^39^ (Fig. 2c).

The NIPBL profile matched RAD21 binding, as illustrated by the enrichment over the *Hoxd1*–*Hoxd4* segment between 84 h and 96 h, that is, when RAD21 signals started to appear (Fig. 2b,c, between red arrows). Signals then spread toward the posterior cluster with a strong re-enforcement up to *Hoxd9* at 132 h, reaching *Hoxd11* at 168 h (Extended Data Fig. 2a). NIPBL binding, thus, matched the activation of the cluster, and NIPBL-enriched peaks colocalized with high H3K27ac (Fig. 2a, lower panel, red arrows). The profile of NIPBL at 168 h precisely matched the H3K27ac profile, with signals covering *Hoxd11* and barely reaching the *Hoxd12* gene. In many respects, these two profiles looked similar, suggesting a link between the recruitment of NIPBL and transcription^40^. NIPBL was slightly enriched at some promoters^41,42^, and a massive coverage was scored over the entire transcribed domains.

### A self-propagating mechanism

The CBSs with an orientation toward T-DOM (CBS1, CBS2, CBS4 and CBS5) showed variations in RAD21 accumulation reflecting their position within the cluster; RAD21 at CBS1 increased upon transcriptional activation of *Hoxd1* to *Hoxd4*, whereas the amount of RAD21 at CBS2, CBS4 and CBS5 increased sequentially in time, following the progression of cohesin loading (Extended Data Fig. 2b). We interpret this dynamic as the persistence at CBSs of cohesin complexes loaded anteriorly due to active transcription, which would extrude until reaching the next CBS with proper orientation. In this view, the decrease in RAD21 accumulation at CBS1 reflects the extension of cohesin loading toward the posterior end of the cluster, leading to an averaging of RAD21 accumulation at all CBSs with an ‘anterior’ (blue) orientation. This is supported by the absence of any change in RAD21 accumulation at CBS6, CBS7, CBS8 and CBS9, whose orientations (red) prevent the accumulation of cohesin loaded onto the active part of the cluster (Extended Data Fig. 2b, bottom).

**Extended Data Fig. 3:**
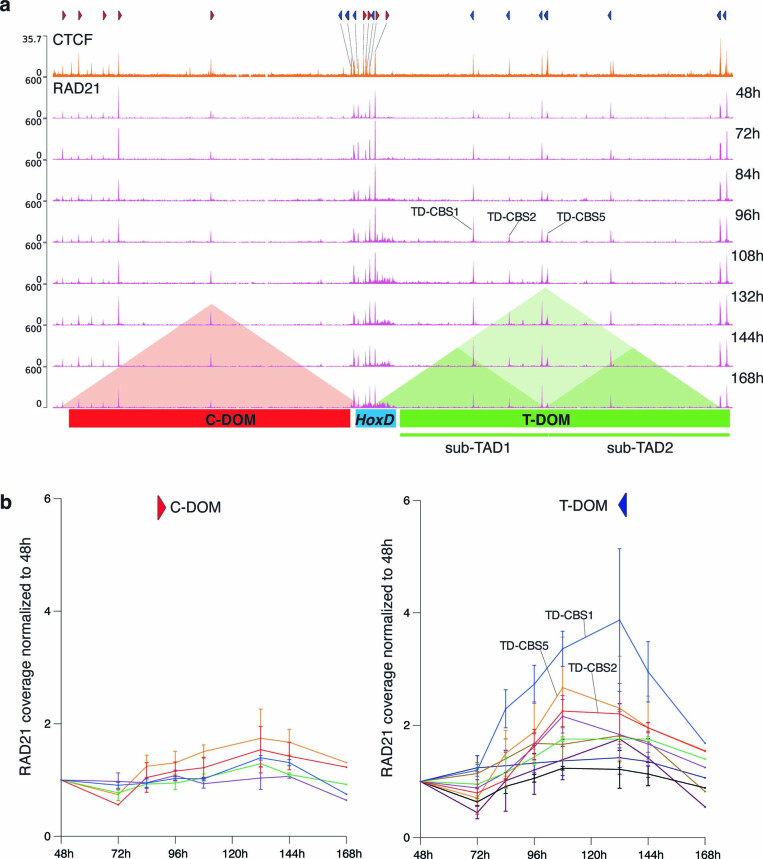
CTCF (a, top) and RAD21 (a, bottom) ChIP-seq profiles over the *HoxD* locus. **a**, The positions and orientations of CBS are indicated with red and blue arrowheads. The labeled CBS within the sub-TAD1 (on the 96 h profile) are those involved with the translocation of the cluster. Genomic coordinates: chr2:73900037-75621560. **b**, RAD21 ChIP-seq coverage over those CBS located outside the cluster, either within T-DOM (right), or within C-DOM (left). The quantification of RAD21 coverage over time (x-axis) indicates a progressive accumulation of extruding cohesin at some CBS located within sub-TAD1 (n = 2; 48 and 168 h: n = 1; 96 and 144 h: n = 3). Line is mean ± SD.

RAD21 accumulation also increased between 72 h and 120 h at T-DOM CBS1, CBS2 and CBS5, three sites interacting with internal *HoxD* CTCF sites due to their convergent orientations^37^ (Extended Data Fig. 3a,b), in support of a function for these T-DOM CBS at these stages, for example, by bringing *Hoxd* genes to the vicinity of T-DOM-located enhancers. The extension of transcription toward a more ‘posterior’ part of the cluster would, in turn, recruit NIPBL and cohesin complexes in between CBSs, leading to extrusion now starting also at more posterior positions, thus feeding a self-propagating mechanism of successive transcriptional activation.

### Dynamics of chromatin topology

**Fig. 3:**
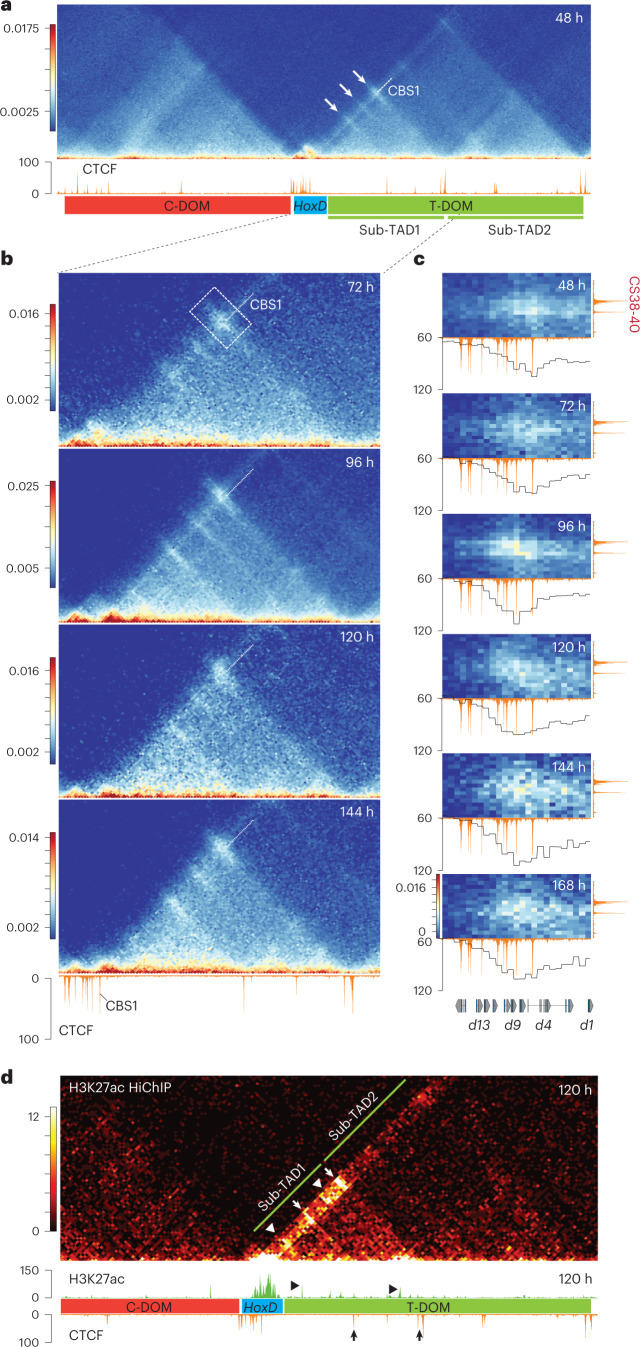
Topological reorganization upon transcriptional activation. **a**,**b**, Timecourse CHi-C at 5-kb resolution using stembryos. **a**, Contacts maps including the *HoxD* cluster (blue rectangle) and both C-DOM (red) and T-DOM (green) TADs. A CTCF ChIP–seq profile is shown below, as for all panels, to help visualize interactions. The arrows indicate strong initial contacts between CBS in the cluster and CBS within sub-TAD1. The line crossing all contact points and prolonged by a white dashed line shows contacts established by CBS1 (quantified in Extended Data Fig. 4). Genomic coordinates—chr2:73900037–75621560. **b**, Magnifications of the *HoxD*–sub-TAD1 interval at various timepoints to show the transition of contacts initially established by CBS1 (dashed white line), then by more posterior CBS in the cluster (see the changing position of the strong contact points relative to the CBS1 dashed line (quantified in Extended Data Fig. 4)). The increase in contacts with sub-TAD1 also includes *Hoxd* gene body and was quantified (Extended Data Fig. 4). Genomic coordinates—chr2:74645050–75200352. **c**, Contacts heatmap between the gene cluster (*x* axis) and the CBS-rich CS38-40 region (*y* axis) at 48–168 h. Bin size is 5 kb. Below each heatmap is a CTCF ChIP track at 168 h and a plot with the average value for each bin representing its interaction with the CS38-40 region. Interactions progressively involve more and more posterior CTCF sites (sum of three independent replicates, 48 h, 96 h and 144 h; single replicate, 72 h, 120 h and 168 h). Genomic coordinates on *x* axis—chr2:74637423–74765000 and on *y* axis—chr2:75105000–75190000. **d**, H3K27ac HiChIP using 120 h wild-type stembryos (top), CTCF (168 h) and H3K27ac (120 h; bottom). Bin size is 10 kb. Higher contact frequency is observed between the acetylated region in the *HoxD* cluster and sub-TAD1 when compared to sub-TAD2, with hotspots matching either the CBS or some acetylated peaks within sub-TAD1 (arrows and arrowheads, respectively). Genomic coordinates—chr2:74190037–75621560.

**Extended Data Fig. 4:**
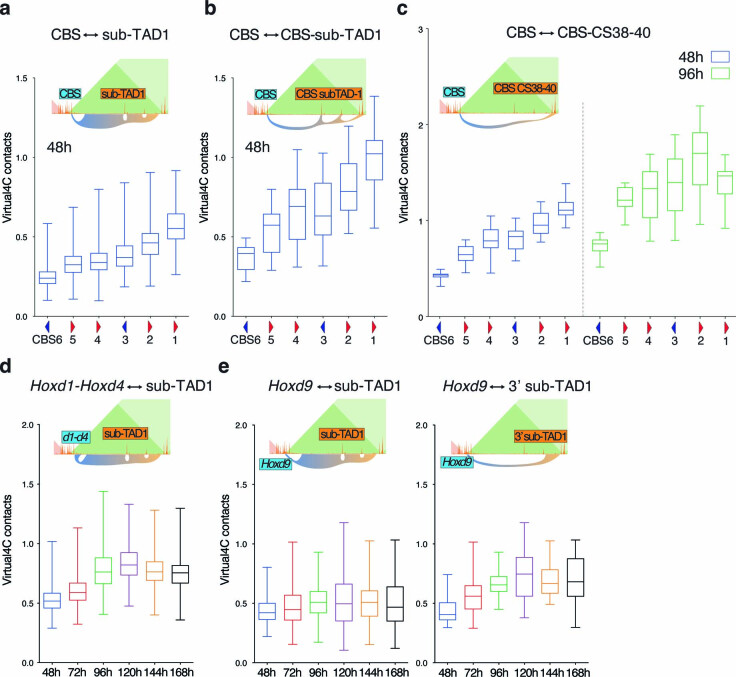
Virtual 4C contacts generated from Capture Hi-C datasets produced from control stembryos. For each quantification, a scheme depicts the viewpoint (blue) and the contact established (orange). **a**, Quantification of contacts between the various CBSs in *HoxD* and regions of sub-TAD1 (sub-TAD1 CBSs excluded) at 48 h. **b**, Quantification of contacts between the various CBSs in *HoxD* and the CBSs located in sub-TAD1 at 48 h. CBS1 establishes more contacts than any other CBS within *HoxD*, with sub-TAD1. **c**, Quantification of contacts between the various CBSs in *HoxD* and those CBSs within the CS38-40 region at 48 h and 96 h, showing the preponderant contacts with CBS2 after activation (96 h). **d**, Contacts between the *Hoxd1-Hoxd4* region and sub-TAD1 increased upon cluster activation at 96 h. **e**, Contacts between *Hoxd9* and sub-TAD1 (left), or the 3′ region of sub-TAD1 only (right) at 96 h show increased interactions with the 3′ part of the regulatory landscape upon activation of the cluster. Box plots with median value and 25–75% percentiles, whiskers represent minimum and maximum (48, 96 and 144 h: sum of 3 independent replicates; 72, 120 and 168 h: single replicate).

We looked at chromatin dynamic and produced a 24-h time-series of capture Hi-C (CHi-C) datasets, from 48 h, that is, before activation (Fig. 3a). Interactions were observed among *Hoxd* genes themselves, likely due to their coverage by H3K27me3 (refs. ^19,43^) leading to a ‘negative’ micro-TAD (Fig. 3a). Also, the cluster displayed several interactions with T-DOM, particularly with sub-TAD1 (Fig. 3a, arrows). Contacts established between CBSs in the cluster and sub-TAD1 were quantified (Extended Data Fig. 4a,b), and CBS1 unambiguously established the strongest contacts of all CBSs within the *HoxD* cluster (Fig. 3a, white dashed line).

After Chiron treatment, this distribution changed and interactions between CBS1 and the sub-TAD1 CBSs (Fig. 3b, 96 h) were no longer the most prominent. Instead, interactions between these latter CBSs and CBS2 became stronger. This shift in the balance of contacts toward a more posterior part of the cluster paralleled the cohesin dynamics, which progressively engaged more posterior CBSs (Extended Data Fig. 3b), suggesting a link between the dynamics of cohesin loading and a topological reorganization of the locus. This was visualized by fixing the position of contacts involving CBS1 (Fig. 3b, white dashed line) and following the position of the main contact (Fig. 3b, boxed in 72 h) moving up relative to this fixed line, that is, involving now more posterior CBSs. Contacts within this boxed area (Fig. 3b,c) were quantified and plotted as curves showing a shift of contact frequencies along with time, from the preferential use of CBS1 to the recruitment of more posterior CBSs (Fig. 3c; compare 48 h with 120 h and see also Supplementary Movie 2).

These directional and stepwise increases in contact frequency with sub-TAD1 (or part thereof) are also applied to the *Hoxd* gene bodies. *Hoxd1* to *Hoxd4* increased contact frequency with the whole sub-TAD1 at 96 h (Extended Data Fig. 4d). *Hoxd9* did not but increased its interactions with the 3′ region of sub-TAD1 (Extended Data Fig. 4e), a region rich in H3K27ac potentially reflecting enhancer–promoter contacts. These interactions were also documented by an H3K27ac HiChIP dataset using 120 h stembryos (Fig. 3d), a stage when anterior genes were activated and cohesin loading was already initiated. The contact map produced a stripe illustrating preferential interactions between the sub-TAD1 region and the active, H3K27ac-positive segment of the *HoxD* cluster, whereas contacts with sub-TAD2 were much less robust (Fig. 3d). The hotspots within the stripe matched either sub-TAD1 CBSs (Fig. 3d, arrows) or acetylated peaks within sub-TAD1 (Fig. 3d, arrowheads), consistent with previous reports that had identified such stripes at active enhancers or within NIPBL-enriched regions^44,45^.

### Cluster opening and cohesin loading

**Extended Data Fig. 5:**
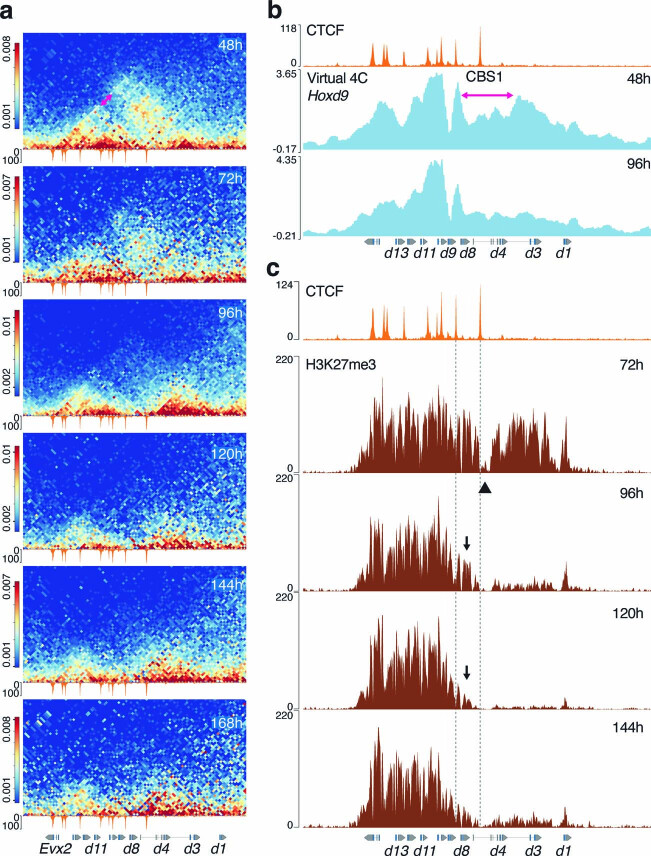
Dynamic reallocation of intra-cluster interactions. **a**, Magnifications of the capture Hi-C map shown in Fig. 3, focusing on the interactions internal to *HoxD* at 2 kb resolution. A CTCF ChIP track at 168 h is shown below each panel and the genes are at the bottom. The double red arrow indicates the looping out of a central part of the cluster. Full translocation of the anterior part is observed at 96 h and a weakening of the negative domain (left) is observed thereafter (48, 96 and 144 h: sum of 3 independent replicates; 72, 120 and 168 h: single replicate). Genome coordinates: chr2:74636423-74780095. **b**, Virtual 4C contacts generated from Capture Hi-C data at 48 h and 96 h, using the *Hoxd9* gene body as a viewpoint (red vertical line). After translocation, the contacts with *Hoxd4*-*Hoxd1* are much reduced. The region of the cluster that initially loops out is indicated with a red arrow in (same as in panel A, top) (sum of 3 independent replicates). **c**, H3K27me3 ChIP-seq of stembryos at various time points, showing the activation of the anterior part, followed by a step wise retraction (black arrows) up to 120 h, when it appears stabilized (see the text). A CTCF ChIP-seq profile is on top for alignment. The fragment looping out is indicated with arrowhead. The two vertical lines help to position CBS1 and CBS2. Genomic coordinates for **b** and **c**: chr2:74616423-74812095.

Next, we analyzed the CHi-C maps at the 2-kb resolution, relative to the CTCF-binding profile (Extended Data Fig. 5a). At 48 h, a virtual 4C profile displayed the negative micro-TAD (Extended Data Fig. 5b), with interactions between *Hoxd9* and the entire cluster (Extended Data Fig. 5b, 48 h). However, a chromatin segment including CBS1, located between *Hoxd4* and *Hoxd8*, showed weaker interactions with the cluster, leading to a ‘gap’ in the micro-TAD (Extended Data Fig. 5a, double arrows), which was also visible using virtual 4C (Extended Data Fig. 5b, double arrows). This ~20-kb region, thus, looped out from the micro-TAD, likely triggered by interactions between CBS1 and T-DOM CBSs (Fig. 3a, top).

**Extended Data Fig. 6:**
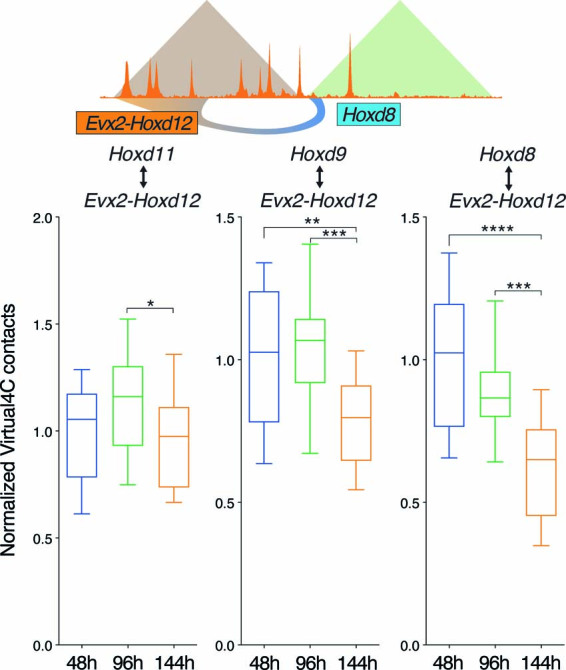
Virtual 4C contacts generated from Capture Hi-C datasets produced out of control stembryos at 48 h, 96 h and 144 h. The contacts between the various viewpoints (*Hoxd* genes shown on top) and the *Evx2-Hoxd12* region showed a progressive decrease upon activation of the cluster, particularly visible for both *Hoxd8* and *Hoxd9*. Contacts values were normalized to 48 h for each viewpoint. Asterisks represent significant changes against as determined by the Kruskal-Wallis followed by Dunn’s multiple comparisons test (* is p-value < 0.05, ** < 0.01, *** < 0.001, **** < 0.0001). Box plots with median value and 25–75% percentiles, whiskers represent minimum and maximum (sum of 3 independent replicates).

After Chiron treatment (72 h), the gene cluster ‘opened’ to form two separate micro-TADs (Extended Data Fig. 5a; compare 48 h and 96 h and see also Supplementary Movie 2). These two micro-TADs represent the transcriptionally inactive part of the cluster on the ‘posterior’ side and the active part on the ‘anterior’ side (Extended Data Fig. 5a, left and right), as further shown by H3K27me3 (Extended Data Fig. 5c). The dynamic of intracluster interactions during this transition was quantified (Extended Data Fig. 6). At 72 h, the region around the position of CBS1 was depleted of H3K27me3 (Extended Data Fig. 5c, arrowhead). At 120 h, H3K27me3 had been further erased toward the centromeric direction between CBS1 and CBS2, again indicating the progression of transcription (Extended Data Fig. 5c).

**Extended Data Fig. 7:**
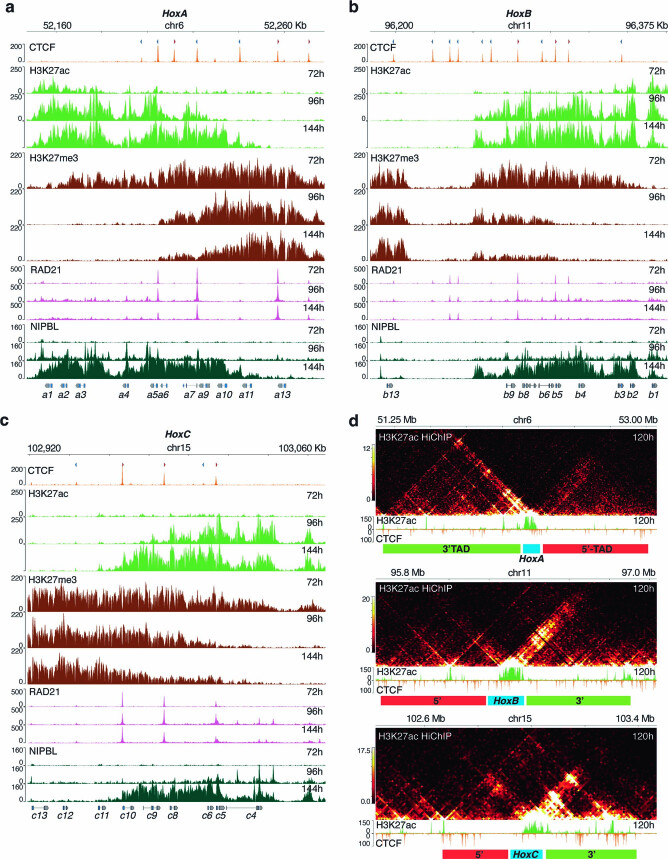
CBS organization, chromatin marks and dynamic activation of genes at the *HoxA*, *HoxB* and *HoxC* clusters. For each gene cluster (**a**, **b** and **c**) are shown a ChIP-seq profile of CTCF binding at 72 h (top) with arrowheads indicating the orientation of each CBS, and H3K27ac, H3K27me3, RAD21 and NIPBL ChIP-seq profiles of stembryos at 72, 96 and 144 h showing the dynamic changes upon the opening of the *Hox* clusters. **d**, H3K27ac HiChIP heatmaps using 120 h wild type stembryos at the *HoxA* (top), *HoxB* (middle) and *HoxC* (bottom) loci. The gene cluster (blue), the 3′TAD (green) and the 5′TAD (red) are shown at the bottom of each locus. Preferential interactions between the active genes at the cluster and the 3′ regulatory region are apparent in all loci. Bin size is 10 kb.

At 96 h, the H3K27ac and H3K27me3 profiles were mutually exclusive, with an H3K27ac domain activated early on and enriched in cohesin complexes, and a transcriptionally inert H3K27me3 domain, a situation observed in all four *Hox* clusters (Extended Data Fig. 7). The boundary between these domains was at the position of CBS1 (Extended Data Fig. 5c, arrowhead). Subsequently, the H3K27me3 domain retracted until 120 h to become apparently stabilized (Extended Data Fig. 5c; compare 120 h with 144 h); meanwhile, the H3K27ac active domain expanded. The apparent stabilization in H3K27me3 distribution at late stages likely reflected a dilution effect, for only very ‘posterior’ stembryonic cells will activate new *Hoxd* genes and hence the vast majority of analyzed cells would keep their H3K27me3 domain unchanged.

This progression was quantified over time, and interactions between the *Evx2–Hoxd12* region and *Hoxd8* decreased already at 96 h (Extended Data Fig. 6, right). Contacts with *Hoxd9* decreased at 144 h only, a timepoint when interactions with *Hoxd11* were only slightly reduced (Extended Data Fig. 6, left). This progression occurred along with the stepwise recruitment of new CTCF sites in the cluster, which interacted with the T-DOM CBS1-5 (Fig. 3a and Extended Data Fig. 3). In the CHi-C maps, this was illustrated by the transition from discrete points of interactions into small bars of increasing length over time, the latter indicating the averaging of different loops between one CTCF site in T-DOM and an increasing number of sites within the *HoxD* cluster (Fig. 3b; compare 72 h and 144 h and see also Supplementary Movie 2).

### A model for the *Hox* timer

**Fig. 4:**
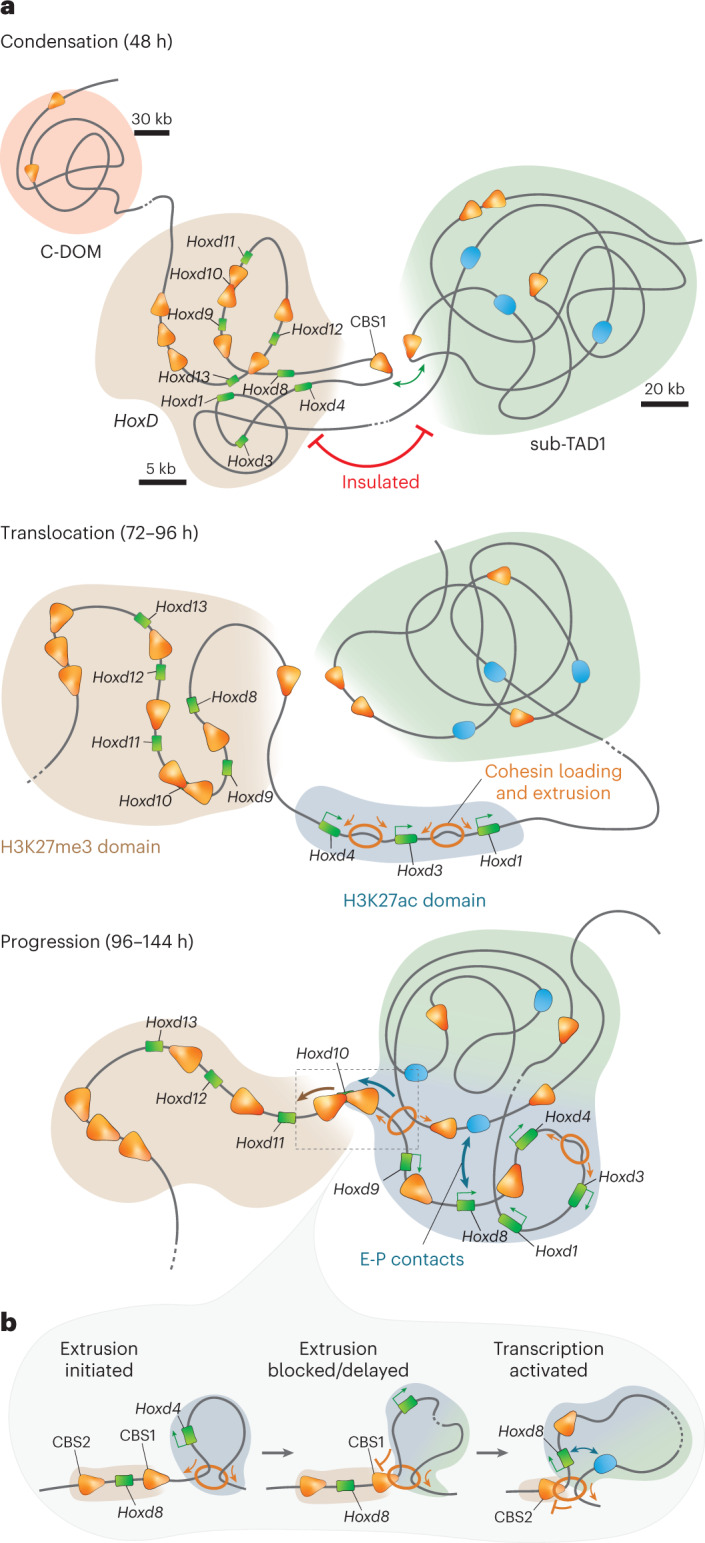
A model for the *Hox* timer. **a**, Three phases in the dynamic activation of the *HoxD* locus are discussed further. (1) Condensation—initially, the cluster is in a condensed state (brown domain), covered by H3K27me3. It is relatively well insulated from T-DOM and its sub-TAD1 (although with constitutive contact points), except for a ~20-kb DNA fragment containing CBS1, which loops out toward the sub-TAD1 located CBS (CBS1, green double arrow). (2) Translocation—while CBS1 keeps contacting the sub-TAD1 CBSs, *Wnt-dependent* transcriptional activation of anterior genes dissociates the cluster into two parts, with a now fully acetylated anterior domain where cohesin complexes will be preferentially loaded (blue domain). (3) Progression—active loop-extrusion in this transcribed part will initially reach CBS1 as a first block, to subsequently reach CBS2 with a given frequency, thus activating *Hoxd8* whose transcription will, in turn, recruit cohesin complexes, and hence a self-entertained mechanism will spread toward the posterior part, concomitantly to a retraction of the H3K27me3 domain. **b**, In this model, the ‘progression’ phase is tightly linked to extrusion by cohesin complexes, with initial recruitment at anterior regions and a delayed progression caused by occupied CTCF sites of the proper orientations. CBSs are represented by orange arrows indicating their orientation, genes are represented by green rectangles, enhancers are represented by blue ovals and cohesin rings are represented by orange circles.

We propose the following model: initially, the cluster is in a globular form, with many internal interactions that are driven by H3K27me3 (refs. ^19,20^; Fig. 4a, top, brown domain). However, a central segment containing CBS1 loops out and contacts several CTCF sites within the flanking T-DOM (Fig. 4a, green domain), in particular, TD-CBS5 (Fig. 4a, top). *Wnt* signaling activates *Hoxd1*, rapidly followed by the anterior genes up to CBS1. This activation coincides with a ‘translocation’ of this anterior part as if the cluster dissociated into two halves, one transcribed (Fig. 4a, middle, blue domain) and the other one still silent (Fig. 4a, middle, brown domain). Transcription in the anterior domain then recruits cohesin complexes (Fig. 4a, middle, blue domain), which start to extrude DNA until extrusion reaches CBS1. Over time and at a given frequency^46^, extrusion will bypass CBS1 and reach CBS2, which like CBS1 will also start looping toward the CBS sites located within sub-TAD1. The gene located in between will, thus, fall into the positive domain and become activated due to its proximity to T-DOM enhancers (Fig. 4a, bottom, E-P contacts). In turn, this newly transcribed gene will be targeted by NIPBL, and cohesin will now be recruited more posteriorly, in addition to the former domain, thus increasing the probability to bypass CBS2 and hitting yet another CBS. This leads to a self-entertained mechanism progressing toward the posterior end of the gene cluster (Fig. 4b), following the successive delays imposed by the ‘insulating’ properties of CTCF sites with an orientation toward sub-TAD1.

Our datasets suggest that this model also applies to other *Hox* clusters, where both the distribution and orientations of CTCF sites are well conserved (Extended Data Fig. 7). In all cases, fast activation of those genes located in the CTCF-free region is accompanied by accumulation of NIPBL and RAD21 and an increase in contacts with the surrounding 3′ regulatory landscape (Extended Data Fig. 7). The further spreading of transcription must also involve upstream factors, for example, CDX proteins^22–24^, which have important roles in trunk extension^47,48^. In our stembryos, analyses of both *Cdx1* and *Cdx2* were compatible with their proposed regulatory function upstream *Hox* genes (Supplementary Note 2 and Supplementary Fig. 3).

### Testing the model

**Fig. 5:**
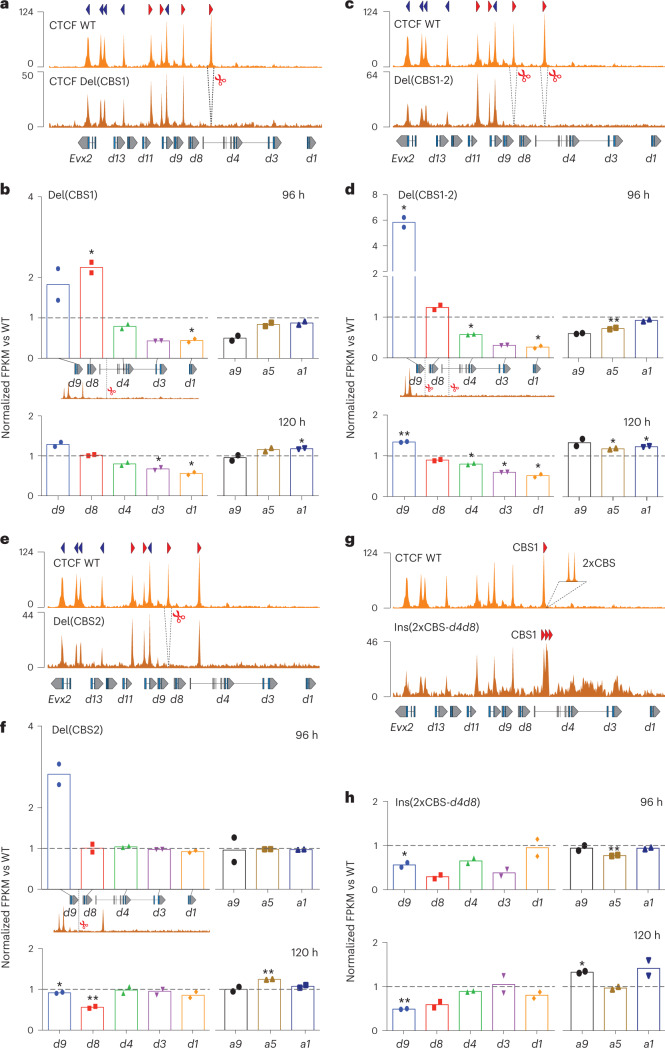
Deletions and insertion of CBS disturb the *Hox* timer. **a**, CTCF ChIP–seq of either control stembryos at 168 h (top) or Del(CBS1) stembryos at 96 h (bottom). Scissors delineate the deletion. **b**, Normalized FPKM values of *Hoxd* and *Hoxa* RNAs from Del(CBS1) stembryos compared to controls at 96 h and 120 h. The vertical line indicates the deleted CBS1. **c**, CTCF control (top) and Del(CBS1-2) (bottom) profiles at 96 h. **d**, Normalized FPKM values of *Hoxd* and *Hoxa* RNAs produced from Del(CBS1-2) stembryos and compared to controls at 96 h (top) and 120 h (bottom). **e**, CTCF control (top) and Del(CBS2) (bottom) profiles at 96 h. **f**, Normalized FPKM values of *Hoxd* and *Hoxa* RNAs from Del(CBS2) stembryos compared to controls at 96 h (top) and 120 h (bottom). **g**, CTCF control (top) and Ins(2×CBS-*d4d8*) (bottom) profiles at 144 h mapped onto an in silico reconstructed mutant genome. **h**, Normalized FPKM values of *Hoxd* and *Hoxa* RNAs produced from Ins(2×CBS-*d4d8*) stembryos and compared to controls at 96 h (top) and 120 h (bottom). In **b**, **d**, **f** and **h**, *n* = two independent samples. Values are represented as means. *P* values were determined by Welch’s unequal variances *t*-test (*P < 0.05 and **P < 0.01). In **e**, the number and orientations of CBS are indicated with red arrowheads. Genomic coordinates—chr2:74636423–74780095.

**Extended Data Fig. 8:**
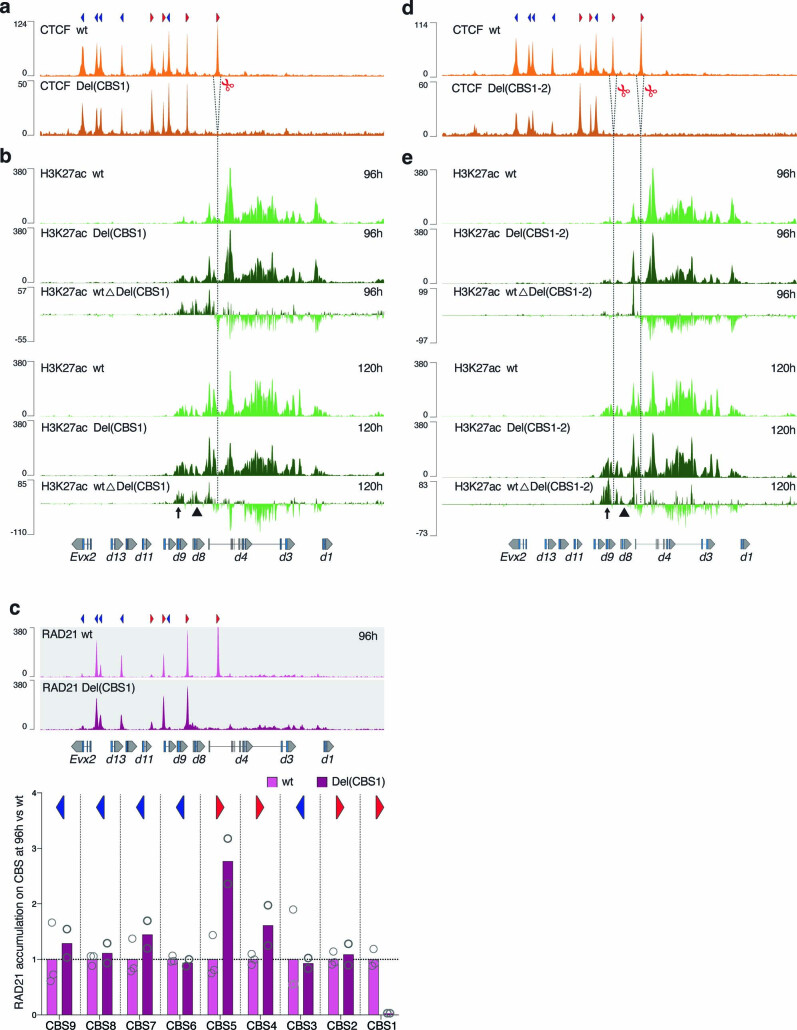
H3K27ac and RAD21 in stembryos lacking CBS1 and CBS2. **a**, CTCF ChIP-seq of control stembryos at 168 h (top) and Del(CBS1) stembryos at 96 h (bottom). The deleted fragment containing CBS1 is indicated with scissors. **b**, H3K27ac ChIP-seq using control and Del(CBS1) stembryos at 96 h (top) and 120 h (bottom) (average of two replicates). A subtraction track with acetylation coverage is shown for each time-point, with colors indicating either control (light green) or Del(CBS1) (dark green) enrichments. The vertical line indicates the position of deleted CBS1, that is, exactly where the inversion of the enrichment occurred. **c**, RAD21 ChIP-seq of control stembryos (top) and Del(CBS1) stembryos (bottom) at 96 h (one replicate shown). Bottom panel show the quantification of RAD21 accumulation at CBS of the *HoxD* locus using ChIP-seq data from Del(CBS1) and compared to control stembryos at 96 h (Del(CBS1): n = 2, wt: n = 3). The orientations of CBS1-9 motifs are shown with red and blue arrowheads. Values are normalized to wt. A redistribution of RAD21 binding toward posterior CBS is observed in the mutant. Values are represented as means. **d**, CTCF ChIP-seq of control stembryos at 168 h (top) and Del(CBS1-2) stembryos at 96 h (bottom). The deleted fragments containing CBS1 and CBS2 are indicated with scissors. **e**, H3K27ac ChIP-seq using control and Del(CBS1-2) stembryos at 96 h (top) and 120 h (bottom) (average of two replicates). A subtraction track with acetylation coverage is shown for each time-point, with colors indicating either control (light green) or Del(CBS1-2) (dark green) enrichments. The vertical lines indicate the position of deleted CBS1 and CBS2. In **b** and **e**, acetylation enrichment at *Hoxd9* and *Hoxd8* is indicated with black arrows and black arrowhead, respectively. Genomic coordinates: chr2:74636423-74780095.

We produced mutant stembryos with individual or multiple CBS deletions. The first mutant lacked CBS1 (Fig. 5a, *HoxD*^*Del(CBS1)*^^−^^*/*^^−^ or Del(CBS1)), and its normalized H3K27ac profile showed enrichment over *Hoxd8* and *Hoxd9*. In contrast, enrichment in the anterior part was decreased (Extended Data Fig. 8). This inversion of H3K27ac difference occurred right at the position of the deleted CBS, and no gain in acetylation was observed for more posterior genes (Extended Data Fig. 8b), suggesting that a faster progression in acetylation had occurred, with CBS1 acting as an initial barrier between the early activated *Hoxd1–Hoxd4* region and more centrally located genes. This was confirmed by RNA-seq (Fig. 5b), with a decreased expression of those genes located anterior to CBS1, whereas *Hoxd8* and *Hoxd9* mRNA levels were higher than in controls. A more robust effect was scored at 96 h than at 120 h for the gain of *Hoxd9* transcripts, while the *Hoxd8* mRNA level was back to normal at 120 h, suggesting that CBS1 impacts the timing of transcription rather than its maintenance. The quantification of RAD21 in the CBS1 deletion showed increased accumulation at CBS4 and CBS5 in the mutant at 96 h (Extended Data Fig. 8c), suggesting a redistribution of the cohesin located at CBS1 along with the premature activation of posterior genes.

We next analyzed stembryos mutant for both CBS1 and CBS2 (Fig. 5c, Del(CBS1-2)). mRNA levels showed a gain in *Hoxd9* at 96 h higher than in Del(CBS1) (Fig. 5d), in contrast with the moderate, if any, increase in *Hoxd8* transcripts level, which was below that observed when only the first CTCF was mutated (Fig. 5b). Weakening of these effects was again observed at 120 h, although the gain of *Hoxd9* was maintained (Fig. 5d), along with an expression boundary shifted anteriorly (Supplementary Note 3 and Supplementary Fig. 4). At 96 h, a decrease in acetylation over the anterior part of the cluster was observed, as well as a clear gain over the TSS of a long *Hoxd4* transcript immediately past the first CTCF site. Gain of acetylation over *Hoxd8* was, however, not observed, whereas a weak signal only was gained over *Hoxd9* (Extended Data Fig. 8d,e, top). This tendency was reinforced at 120 h, with a gain over *Hoxd9* stronger than in the Del(CBS1) mutant (Extended Data Fig. 8b,e, bottom, black arrows), whereas the gain over *Hoxd8* was weaker than in the Del(CBS1) mutant (Extended Data Fig. 8b,e, bottom, black arrowheads). We concluded that the main upregulation effect upon removing a CBS was observed for the gene located in 5′ of this CBS, that is, *Hoxd8* for Del(CBS1) and *Hoxd9* for Del(CBS1-2), a conclusion further challenged by deleting CBS2 alone (Del(CBS2); Fig. 5e). In these mutants, expression of all anterior genes and of *Hoxd8* remained unchanged at 96 h, whereas a clear increase in *Hoxd9* transcripts was scored (Fig. 5f), which again was no longer observed at 120 h.

**Extended Data Fig. 9:**
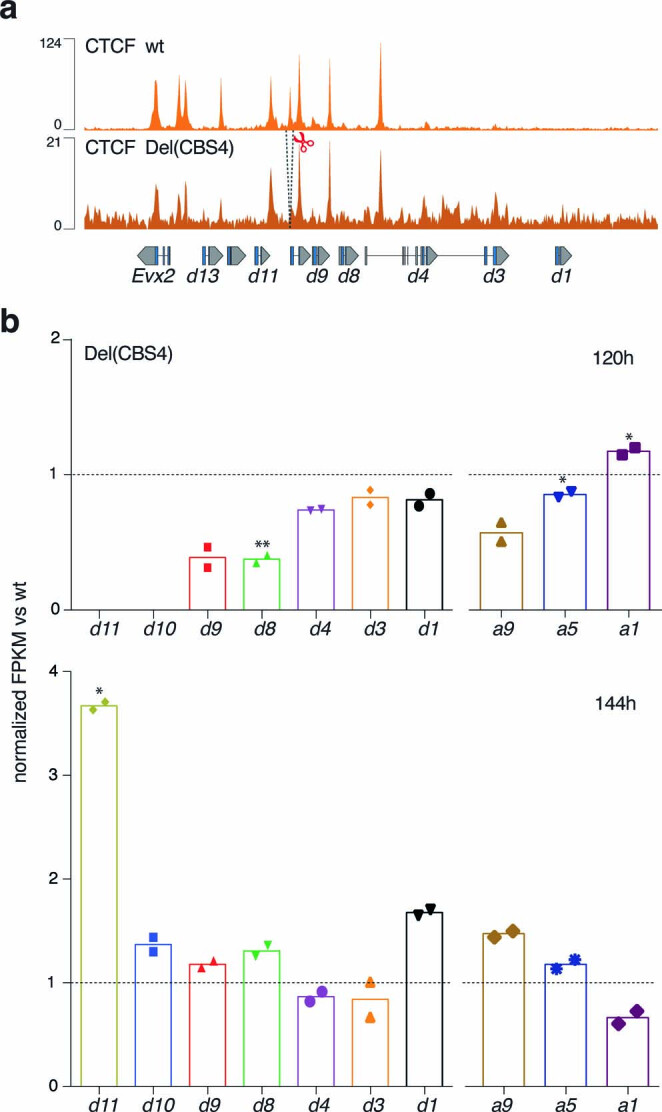
Deletion of CBS4 and effect on the surrounding *Hoxd* genes. **a**, CTCF ChIP-seq of control at 168 h (top) and Del(CBS4) at 120 h stembryos (bottom). The deleted fragment containing CBS4 is indicated with scissors and dashed lines. Genomic coordinates: chr2:74636423-74780095. **b**, Normalized FPKM values of *Hoxd* and *Hoxa* genes obtained from RNA-seq libraries (n = 2) produced from mutant Del(CBS4) stembryos and compared to controls at both 120 h and 144 h (expression of *Hoxd10* and *Hoxd11* was not considered at 120 h). Values are represented as means. P-values were determined by Welch’s unequal variances t-test (* is p-value < 0.05, ** < 0.01).

At this late stage, a decrease in the expression level of *Hoxd8* was observed, indicating that CBS2 may participate in the maintenance of *Hoxd8* expression. A similar effect was observed when CBS4 was deleted alone (Extended Data Fig. 9, Del(CBS4)), because expression of *Hoxd8* and *Hoxd9*, the genes located anterior to the deleted CBS4, was decreased at 120 h (Extended Data Fig. 9). In contrast, the *Hoxd11* mRNA level, that is, the gene located in 5′ of CBS4, was clearly upregulated at 144 h. Overall, these results point to a dual function for these CTCF sites; while CBSs are used as insulators for genes positioned posteriorly, as exemplified by CBS2 and *Hoxd9*, they are also used as anchoring points for those genes located anteriorly, as observed with CBS2 and *Hoxd8*. As a result, the deletion of a CBS site will tend to activate prematurely the gene positioned in 5′, while it will fail to maintain the expression of the 3′-located gene at its normal level.

We also inserted two supernumerary CBSs within a 2-kb distance next to CBS1. In these stembryos, three CTCF sites, instead of one, are now ‘opening’ the series of CBSs, all oriented toward sub-TAD1 (Fig. 5g, Ins(2×CBS-*d4d8*)). mRNA levels of several genes were substantially decreased at 96 h (from *Hoxd3* to *Hoxd9*; Fig. 5h, top). At 120 h, the genes positioned posterior to the three compact CBSs (*Hoxd8* and *Hoxd9*) were still affected in their transcription, suggesting that the initial delay in the expression of central genes was persistent in this case (Fig. 5h, bottom). In addition, the effects of larger deletions of both the anterior part of the cluster and sub-TAD1 were analyzed (Supplementary Note 4 and Supplementary Figs. 5 and 6).

### CTCF as a timekeeper

**Fig. 6:**
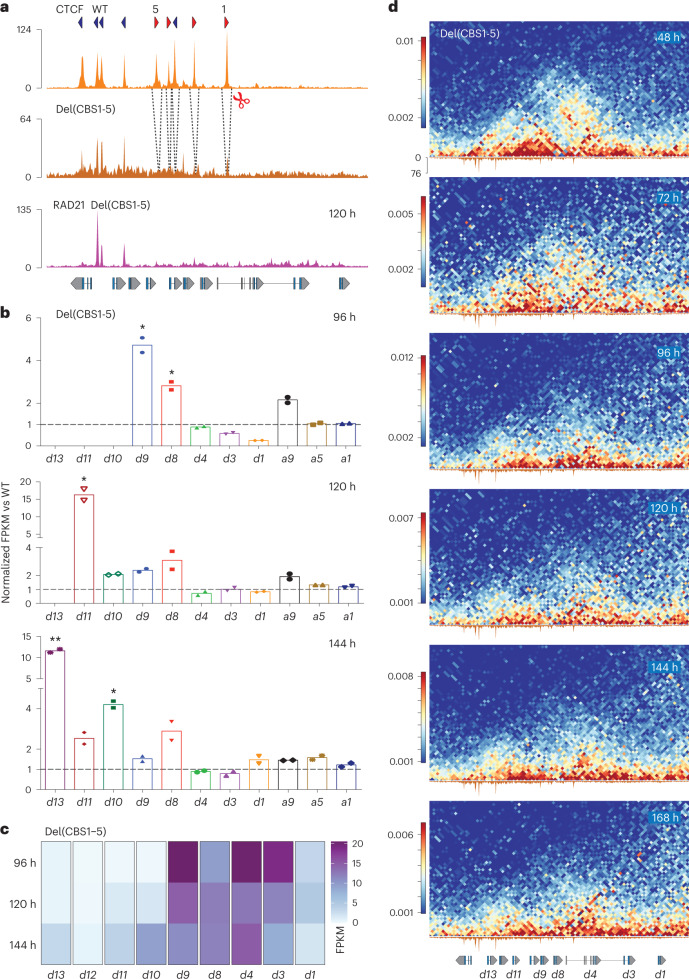
Impact of multiple CBS deletions *in cis* on the *Hox* timer. **a**, CTCF ChIP–seq data from control at 168 h (top) and Del(CBS1-5) mutant stembryos at 96 h (middle). Deleted CBS are indicated with dashed lines. The RAD21 profile at 120 h (bottom) shows no accumulation at the deleted sites (compare with Fig. 2b). Genomic coordinates—chr2:74636423–74780095. **b**, Normalized FPKM values for *Hoxd* and *Hoxa* RNAs from Del(CBS1-5) stembryos compared to control at 96 h, 120 h and 144 h. Expression of *Hoxd10* and *Hoxd11* was not considered at 96 h, nor that of *Hoxd13* at 96 h and 120 h. Increased expression was observed for central and posterior *Hoxd* genes in mutant stembryos (*n* = 2). Values are represented as means. *P* values were determined by Welch’s unequal variances *t*-test (*P < 0.05 and **P < 0.01). **c**, Heatmap of FPKM values for *Hoxd* genes transcripts in Del(CBS1-5) mutant stembryos at various timepoints (average of two replicates). **d**, Timecourse CHi-C using Del(CBS1-5) stembryos. Bin size is 2 kb, and libraries are mapped onto wild-type genome (mm10). When compared to the control specimen (Extended Data Fig. 5), the translocation (if any) of the cluster into two globules is very much perturbed. Genomic coordinates—chr2:74636423–74780095.

**Extended Data Fig. 10:**
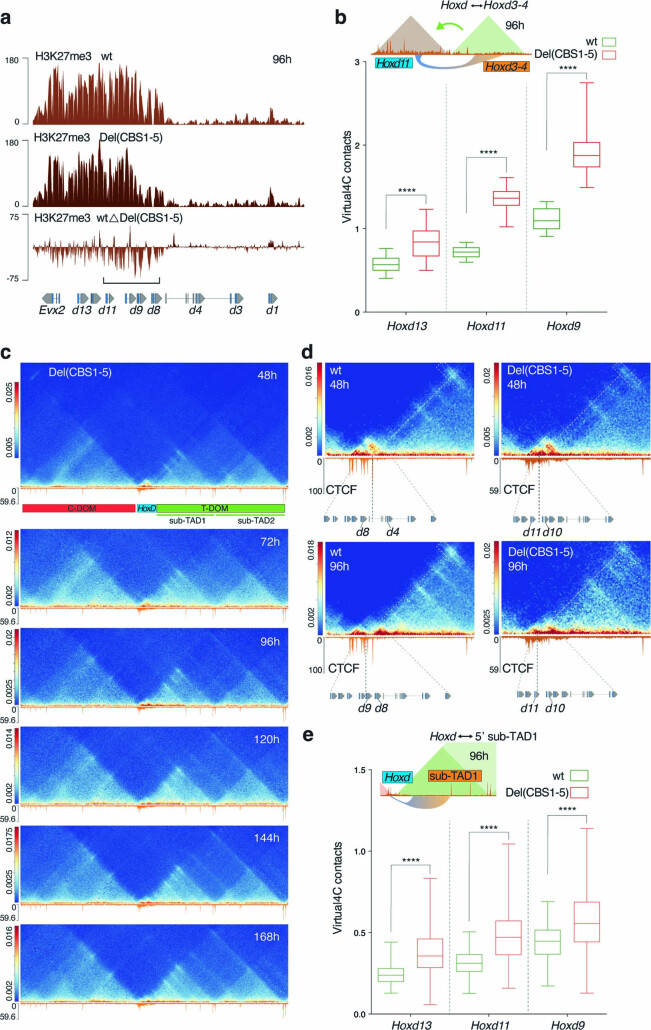
H3K27me3 profile and capture Hi-C using Del(CBS1-5) stembryos. **a**, H3K27me3 ChIP-seq using control (top, light brown) and Del(CBS1-5) (middle, dark brown) stembryos at 96 h (average of two replicates). The subtraction track is shown below and illustrates the decrease in H3K27me3 marks over the central and posterior part of the *HoxD* cluster (bracket). Genomic coordinates: chr2:74646423-74780095. **b**, Virtual4C contacts generated from Capture Hi-C datasets produced out of control and Del(CBS1-5) mutant stembryos at 96 h. Interactions are quantified between those *Hoxd* genes shown below (viewpoint) and the activated *Hoxd3*-*Hoxd4* part of the cluster. Increased contacts are scored in mutant stembryos for all three viewpoints, illustrating the loss, in CTCF mutant stembryos, of a clear partition of the gene cluster into two separate positive and negative domains. **c**, Time course capture Hi-C using Del(CBS1-5) stembryos. Six time points are shown with bin size 5 kb. Libraries were mapped on a control genome (mm10). Genomic coordinates: chr2:73900037-75621560. **d**, Magnifications of the interactions between the *Hoxd* genes cluster and the CS38-40 region (dashed box) in control (left) and Del(CBS1-5) mutant specimen (right), either before (48 h, top) or after (96 h, bottom) the translocation of the anterior part of the cluster. Diagonal and vertical dashed lines indicate the position of the most prominant interactions within the *Hoxd* gene cluster with the CS38-40 region. While a shift toward a posterior position is observed between 48 h and 96 h in control stembryos (from CBS1 to the *Hoxd8*-*Hoxd9* interval), interactions scored in the Del(CBS1-5) mutant condition remain diffused over the gene cluster and only a slight change in the posterior limit is observed (around *Hoxd11*). Genomic coordinates: chr2:74590000-74985000 **e**, Virtual4C contacts generated from Capture Hi-C datasets produced in control and Del(CBS1-5) mutant stembryos at 96 h. Interactions between the indicated *Hoxd* genes (viewpoints) and the 5′ region of sub-TAD1 are significantly higher in Del(CBS1-5) mutant. In **b** and **e**, box plots with median value and 25–75% percentiles, whiskers represent minimum and maximum. Asterisks represent significant changes determined by the Mann-Whitney nonparametric test (**** is p-value < 0.0001) (wt: sum of 3 independent replicates, Del(CBS1-5) mutant: single replicate).

Finally, we processed stembryos lacking from CBS1 to CBS5 in *cis* (Fig. 6a). Expression of posterior genes was upregulated at all stages analyzed (Fig. 6b). At 120 h, both *Hoxd10* and *Hoxd11* expressions were gained and at 144 h, *Hoxd13* expression was also gained. Therefore, the absence of CTCF in the anterior and central parts of the cluster leads to the premature activation of those genes located 5′ to CBS1. Premature activation was confirmed by comparing normalized H3K27me3 profiles between control and CBS1-5 mutant stembryos at 96 h (Extended Data Fig. 10a). While the anterior part was mostly similar to the control, the entire H3K27me3 domain up to the *Evx2* gene was ‘weakened’, with a particular loss over the *Hoxd11* to *Hoxd8* region (Extended Data Fig. 10a, bracket at the bottom), and the whole cluster, thus, became somehow transcriptionally leaky. However, the boundaries of the H3K27me3 domain did not change in mutant stembryos, indicating that CTCF does not interfere with the initial distribution of polycomb marks in mES cells. Despite this severe impact on the timing of activation, a weak tendency to follow a colinear activation was still observed, in particular, at late stages and from *Hoxd9* onwards (Fig. 6c; compare 120–144 h).

A time series of CHi-C datasets with Del(CBS1-5) stembryos showed changes in the dynamic of chromatin architecture compared to controls (Fig. 6d). First, the *Wnt*-dependent translocation of the cluster into two domains was no longer observed. Second, at 96 h, the progression of contacts toward more posterior parts of the cluster was not observed either in mutant stembryos. Instead, after Chiron treatment, the anterior part did not clearly segregate from the central and posterior regions and contacts remained distributed throughout (Fig. 6d), reflecting a diffused but general activation at a time when controls display two well-ordered negative and positive domains with a dynamic transition from the former to the latter. This was quantified by measuring virtual 4C contacts between either *Hoxd13*, *Hoxd11* or *Hoxd9* and the *Hoxd3–Hoxd4* region, which were markedly increased in the mutant specimen (Extended Data Fig. 10b).

When looking at contacts between the cluster and sub-TAD1 region 38–40, the stepwise extension of the loops toward more posteriorly located CBS observed in control was not scored in mutant specimens. Instead, contacts were detected throughout the cluster, already from an early stage onwards, which translated into a bar rather than a spot in the CHi-C map (Extended Data Fig. 10d). Altogether, these results indicated that CTCF proteins are both mandatory to protect posterior *Hoxd* genes to be contacted prematurely and essential for the precision and the pace of the *Hox* timer, although a remnant of a colinear process was observed in their almost complete absence.

## Discussion

In this study, we used stembryos to analyze the mechanism underlying the *Hox* timer, due to their prominent ‘posterior’ identities^34,49,50^ and consequent enrichment in cells implementing this process. However, the progressive restriction of expression domains toward the posterior aspect of elongating stembryos^34^ dilutes any measured parameters and, thus, underestimates them, a bias for which we decided not to introduce any correction index. This is particularly important to consider when small increases are observed, such as the posterior spreading of elongating RNA pol II, which are thus underestimated in this study. This is also visible with the incomplete retraction of H3K27me3 upon activation of posterior genes because the samples analyzed over time contain increasing amounts of negative cells for any newly activated gene due to its increasing posterior restriction.

We propose a three-step mechanism for the *Hox* timer. The first step is condensation in which the *Hox* clusters tend to condense into a negative globule, covered with H3K27me3. Although this had been observed previously in mES cells^19,20^, stembryos revealed that a central part of the cluster containing CBS1 loops out of this negative globule and is tightly anchored to several CBS located within the flanking sub-TAD1, prefiguring the two microdomains to come, as if the cluster would be already in a pre-activation configuration. During the second translocation step, a quasi-simultaneous activation of the CTCF-free region occurs, along with a rapid deposition of a paused form of RNA pol II up to *Hoxd10*, its ser2 phosphorylation subsequently occurring in a time sequence.

During this translocation phase, the anterior cluster dissociates from the posterior part and cohesin complexes are loaded onto this early transcribed segment^40–42^, with loop-extrusion mostly involving CBS1. Then extrusion recruits more posteriorly located CBS during the progression phase, a spreading documented by our timecourse interaction profiles and also suggested by the change in the relative distribution of RAD21 over the CTCF sites.

Whether the mechanism of progression, that is, what makes loop-extrusion going through CBS1 at some point to reach CBS2, is active or passive is hard to evaluate. A passive process where extrusion would go through CBS1 either due to a punctual lack of CTCF binding or due to a leakage in the blocking efficiency of a bound CTCF, in both cases at a given frequency, could certainly account for the progression^46^. Indeed, each event of this kind, leading to the transcription of the gene located more ‘posteriorly’, would recruit cohesin at the promoter of the newly transcribed gene^40,51^ and, thus, promote extrusion from a more posterior position. After a certain time, cohesin loading would have progressed toward more posterior genes, along with their transcription, while still being loaded in 3′ of CBS1. Alternatively, the progression could be actively regulated by some CTCF cofactors such as MAZ, which was shown to contribute to CTCF insulation in the HoxA cluster^52^, or by the WAPL-binding partner PDS5, which also contributes to boundary function^53^. In any case, this mechanism could account for the conservation in the number, positions and orientations of CTCF sites among paralogous mammalian clusters, in particular for the rather regular spacing between these CTCF sites, a feature that had remained unexplained when considered only in the context of a TAD boundary, which does not require such an iterated organization (for example, ref. ^54^).

The initial transcriptional asymmetry is likely imposed by *Wnt* signaling, which impacts *Hox* genes located in the ‘anterior’ part of the cluster^21^. From there, an intrinsic progression in transcription toward the opposite end of the gene cluster may naturally occur in this gene-dense environment, due to the presence of the transcription machinery, factors and chromatin remodelers. In this context, the series of CTCF sites would determine the precision of the process as well as its proper sequence and pace, rather than being mandatory for a global time delay in transcription to occur. From an evolutionary viewpoint, the separation between an intrinsic tendency to produce a time sequence in the activation of a series of genes in *cis* and the mechanism regulating the pace and the precision of this mechanism may allow some adaptive flexibility. Indeed, a slight variation in the timing of activation may have important consequences upon axial structures, and such variations might have been essential to produce the distinct *Hox* combinations observed in different vertebrates^55,56^. For example, changes in the number of CTCF sites, their positions or even their binding affinities may have triggered modifications in the expression timing of *Hox* genes. On the other hand, the presence of four *Hox* gene clusters containing similarly organized CTCF sites could make the overall collinear process robust and resilient to potential perturbations.

## Methods

### Culture of gastruloids

mES cells were routinely cultured in gelatinized tissue-culture dishes with 2i^57^ LIF DMEM medium composed of DMEM + GlutaMAX supplemented with 10% mES certified FBS, nonessential amino acids, sodium pyruvate, beta-mercaptoethanol, penicillin/streptomycin, 100 ng ml^−1^ of mouse LIF, 3 µM of GSK-3 inhibitor (CHIR99021) and 1 µM of MEK1/2 inhibitor (PD0325901). Cells were passaged every 3 d and maintained in a humidified incubator (5% CO_2_, 37 °C). The differentiation protocol for gastruloids was previously described^34^. Briefly, mES cells were collected after Accutase treatment, washed and resuspended in prewarmed N2B27 medium (50% DMEM/F12 and 50% Neurobasal supplemented with 0.5× N2 and 0.5× B27). In total, 300 cells were seeded in 40 µl of N2B27 medium in each well of a low-attachment, rounded-bottom 96-well plate. Forty-eight hours after aggregation, 150 µl of N2B27 medium supplemented with 3 µM of GSK-3 inhibitor was added to each well. In total, 150 µl of the medium was then replaced every 24 h. Collection of gastruloids for each timepoint was performed indiscriminately up to 96 h after aggregation. Starting from 108 h, only those gastruloids showing a clear elongating shape with no sign of apoptosis were collected and processed for subsequent analysis. Because gastruloids grown under various protocols in various laboratories can be very different from one another (for example, refs. ^34,50,58^), we refer to them as stembryos throughout the paper to prevent confusion.

### Generation of mutant mES cells

Wild-type mES cells (EmbryoMax 129/SVEV) were used to generate mutant cell lines following the CRISPR/Cas9 genome editing protocol described in ref. ^59^. sgRNA targeting guides (Supplementary Table 1) were cloned into a Cas9-T2A-Puromycin expressing plasmid containing the U6-gRNA scaffold (gift of A. Németh; Addgene plasmid, 101039). mES cells were transfected with 8 µg of plasmid using the Promega FuGENE 6 transfection kit and dissociated 48 h later for puromycin selection (1.5 µg ml^−1^). Clone picking was conducted 5–6 d later, and positive mES cell clones were assessed by PCR screen using the MyTaq PCR mix kit (Meridian Bioscience) and specific primers surrounding the targeted region (Supplementary Table 2). Mutations were verified for both alleles by Sanger sequencing (Supplementary Table 3). When heterozygous mutations were obtained, the transfection procedure was reiterated until homozygous mES cell clones were obtained. For the Del(CBS1-2) mutant, the deletion of CBS2 was carried out on top of Del(CBS1) mES cells. The Ins(2×CBS-d4d8) cell line was produced by the insertion of a 900-bp DNA cassette at a 2-kb distance of 5′ to CBS1. The cassette contains two CTCF sites with the same orientation as CBS1. The transfection was performed with 1.5 µg of the plasmid containing the recombination cassette along with 8 µg of the sgRNA plasmid. The Del(CBS1-5) and Del(sub-TAD1) mES cell lines were derived from mouse blastocysts at the Mouse Clinical Institute (www.ics-mci.fr). The Del(CBS1-5) mouse is described in ref. ^28^. Del(sub-TAD1) mice were obtained by electroporating the CRISPR guide (Supplementary Table 1) and the *Cas9* mRNA into fertilized mouse embryos. The deletion covers the sub-TAD1 region including *the Mtx2* gene, but not the CS38-40 boundary. To overcome *Mtx2* knockout homozygous lethality, mice carrying a transgene, a modified mouse fosmid containing a sequence covering the *Mtx2* region (based on RP24-284D11 from https://bacpacresources.org; ref. ^60^), were crossed with the Del(sub-TAD1) mice for rescue.

### Laboratory animals

Ten-week-old mice (*Mus musculus*) were used for mES cell line derivation. Animals were kept in a continuous back cross with BL6 × CBA F1 hybrids. Mice were housed in the University of Geneva Sciences III animalerie, with light 07:00–19:00 in the summer and 06:00–18:00 in winter. The ambient temperatures were maintained between 22–23 °C and 45–55% humidity, and the air was renewed 17 times per hour. All experiments with animals were performed in agreement with the Swiss Law on Animal Protection (LPA) under license numbers GE 81/14.

### Next-generation sequencing analysis and figure generation

All NGS analyses, except for single-cell RNA-seq, were performed on a local installation of galaxy^61^. The calculations for single-cell RNA-seq have been performed using the facilities of the Scientific IT and Application Support Center of EPFL. All command lines and scripts to regenerate figures are available at https://github.com/lldelisle/scriptsForRekaikEtAl2022 (ref. ^62^). Most of the genomic tracks were plotted using pyGenomeTracks version 3.7 (refs. ^63,64^) and modified with Illustrator 2022. All boxplots and bar plots were plotted using Prism 9, and heatmaps were plotted with R (www.r-project.org).

### Mutant genomes in silico

Chromosome 2 matching the three mutants (Ins(2×CBS-d4d8), Del(d1-d4) and Del(sub-TAD1)) was generated manually using the chromosome 2 sequence from UCSC of mm10 and results from Sanger sequencing of the breakpoints^65^. The mutant chromosome 2 sequence was concatenated with the sequences of all other autosomes, chr X, chr Y and mitochondrial DNA from mm10 (UCSC).

### RNA-seq

Stembryos for each condition were collected and pooled in a 2-ml Eppendorf tube. After centrifugation and medium removal, pelleted stembryos were stored at −80 °C until RNA extraction. RNeasy Plus Micro kit (Qiagen) with on-column DNase digestion was used for RNA extraction following the manufacturer’s instructions. RNA-seq library preparation with Poly-A selection was performed with 1 µg of purified RNA using the TruSeq Stranded mRNA kit from Illumina and following the manufacturer’s protocol. Library quality was assessed with a fragment analyzer before sequencing on a NextSeq 500 sequencer as paired-end, 75-bp reads. For data analysis, adapter sequences were trimmed from reads using cutadapt^66^ version 1.16 (-a ‘GATCGGAAGAGCACACGTCTGAACTCCAGTCAC’ -A ‘GATCGGAAGAGCGTCGTGTAGGGAAAGAGTGTAGATCTCGGTGGTCGCCGTATCATT’). Trimmed reads were aligned on mm10 with STAR version 2.7.7a^67^ with ENCODE options using a custom gtf based on Ensembl version 102 (ref. ^68^). Only uniquely mapped reads (tag NH:i:1) were kept using bamFilter version 2.4.1 (ref. ^69^). FPKM values were computed using cufflinks version 2.2.1 (refs. ^70,71^) with default parameters. To reduce the impact of variations in gastruloid growth speed, the FPKM values of *HoxD* genes (except *Hoxd13*) and some *HoxA* genes (*Hoxa1*, *Hoxa5* and *Hoxa9*) were normalized using FPKM values of genes from other clusters (*Hoxa* and *Hoxc* genes were used to normalize *Hoxd*, whereas *Hoxb* and *Hoxc* genes were used to normalize *Hoxa*; see GitHub repository for more details).

### Single-cell RNA-seq

Stembryos were grown to 96 h and 120 h after aggregation, and those that failed to elongate at 120 h were not used. In total, 48–96 stembryos were collected at 96 h or 120 h after aggregation and washed twice in 1 ml of PBS. Samples were dissociated in 100 µl Accutase for 5 min at 37 °C. Full dissociation was achieved by mechanical dissociation (pipetting) and verified to ensure the absence of doublet cells. Stembryonic cells were then washed twice in 500 µl PBS and resuspended in an adequate volume of PBS 0.04% BSA at 10^6^ cells per ml. All centrifugation steps were done for 5 min at 350*g* in DNA low-binding Eppendorf tubes. Single-cell suspensions were filtered using Flowmi Cell Strainer (40 µm) and subjected to single-cell RNA-seq using the 10x Genomics platform (V3 and V3.1 chemistry) following the manufacturer’s recommendation. Up to 6,000 cells were targeted per sample. cDNA preparations were performed according to 10x Genomics recommendations, amplified for 10–12 cycles and sequenced on a HiSeq 4000 with the cbot2 chemistry. At least two replicates were performed for each timepoint. In addition, a third 120-h timepoint was generated by dissection in two portions to separate the anterior and posterior halves, and each portion was analyzed separately by single-cell RNA-seq. For data processing, the Cell Ranger pipeline version 6.0 (10x Genomics) was used with the default parameters on each fastq pair of single-cell RNA-seq datasets to perform the alignment, by applying filtering and count barcodes and UMIs. Alignment was performed against the mouse reference genome mm10 and a modified gtf file based on Ensembl version 98 (ref. ^72^). Each gene–cell count matrix was then analyzed using Seurat version 4.0.2 (ref. ^73^). A Seurat object was generated by filtering out barcodes with less than 200 identified gene and genes identified in less than three cells. Then, further filtering was applied to remove low-quality cells and doublets. For each dataset, the mean RNA count was used, and cells with either less than 40% of the mean or more than 2.5-fold of the mean were removed. In addition, cells with more than 8% or less than 0.05% mitochondrial counts were removed. Each dataset was then normalized using the NormalizeData command, the 3,000 most variable features were identified and the cell cycle was scored (using the 2019 updated gene list from Seurat). Then, all individual datasets were merged into a single Seurat object. The merged dataset was then normalized, and the 3,000 most variable features were identified. The data scaling was performed using the ScaleData command using the percentage of mitochondrial count and cell cycle score.

### ChIP and ChIPmentation (ChIP-M)

ChIP and ChIP-M experiments were performed according to the protocol described in refs. ^37,74^ and adapted for stembryos samples. Briefly, collected stembryos were pooled in a 15 ml falcon tube, washed with PBS and resuspended in 1 ml PBS containing 1% formaldehyde for fixation for 10 min at room temperature. The crosslink reaction was stopped by adding a glycine solution to a final concentration of 0.125 M. Fixed stembryos were pelleted and stored at −80 °C until further use. Samples were resuspended in a sonication buffer (Tris–HCl, pH = 8.0, 50 mM; EDTA, 10 mM; SDS, 0.25% and protease inhibitors) and sonicated in a Covaris E220 device for 14 min (duty cycle 2% and peak incident power 105 W) to obtain an average chromatin fragment size of 300–500 bp. A dilution buffer (HEPES, pH = 7.3, 20 mM; EDTA, 1 mM; NP40, 0.1%; NaCl, 150 mM and protease inhibitors) was added to the sonicated chromatin and incubated with the antibody–bead complex (Pierce Protein A/G Magnetic Beads, Thermo Fisher Scientific) overnight at 4 °C. Sequential washes were then performed twice with RIPA buffer (Tris–HCl, pH = 8.0, 10 mM; EDTA, 1 mM; sodium deoxycholate, 0.1%; TritonX-100, 1%; NaCl, 140 mM and protease inhibitors), RIPA High salt buffer (Tris–HCl, pH = 8.0, 10 mM; EDTA, 1 mM; sodium deoxycholate, 0.1%; TritonX-100, 1%; NaCl, 500 mM and protease inhibitors), LiCl buffer (Tris–HCl, pH = 8.0, 10 mM; EDTA, 1 mM; LiCl, 250 mM; sodium deoxycholate, 0.5%; NP40, 0.5% and protease inhibitors) and Tris–HCl buffer (pH = 8.0, 10 mM and protease inhibitors). For ChIP experiments (4 µg H3K27ac (Abcam, ab4729), 5 µg H3K27me3 (Active Motif, 39155) and 8 µg PolII-Ser2p (Millipore, 04-1571)), DNA fragments were incubated in the elution buffer (Tris–HCl, pH = 8.0, 10 mM; EDTA, 5 mM; NaCl, 300 mM and SDS, 0.1%) containing proteinase K and purified using the Qiagen MiniElute kit. A phosphatase inhibitor (PhosStop, Roche) was used during PolII-Ser2p ChIP incubation and beads wash. The DNA library was produced using TruSeq adapters and amplified with the Kapa HiFi library kit (Roche) using a number of cycles determined by qPCR. For ChIP-M samples (5 µg CTCF (Active Motif, 61311), 3 µg PolII (Abcam, ab817), 5 µg RAD21 (Abcam, ab992) and 5 µg NIPBL (Bethyl Laboratories, A301-779A)), DNA fragments bound to the antibody–bead complex were tagmented using the Nextera tagmentation kit. Beads were resuspended in the tagmentation buffer and incubated at 37 °C for 2 min with 1 µl of the Tn5 transposase. Fragments were then eluted and purified, as described previously, and amplified using Nextera primers. Final DNA libraries were purified and size selected using AMPure XP magnetic beads (Beckman Coulter), and fragment analysis was performed before sequencing on a NextSeq 500 sequencer as paired-end, 75-bp reads. For data analysis, fastqs of the three replicates of wt_144 h_pSer2PolII were concatenated before any processing. Adapter sequences and bad quality bases were trimmed from reads using cutadapt^66^ version 1.16 (-a ‘GATCGGAAGAGCACACGTCTGAACTCCAGTCAC’ -A ‘GATCGGAAGAGCGTCGTGTAGGGAAAGAGTGTAGATCTCGGTGGTCGCCGTATCATT’ for ChIP and -a ‘CTGTCTCTTATACACATCTCCGAGCCCACGAGAC’ -A ‘CTGTCTCTTATACACATCTGACGCTGCCGACGA’ for ChIPmentation --quality-cutoff=30). Trimmed reads were mapped on the mm10 genome (or the mutant genome Ins(2×CBS-d4d8) for the corresponding mutant CTCF ChIP-M) using Bowtie2 version 2.3.4.1 (ref. ^75^). Only alignments with proper pairs and mapping quality above 30 were kept using Samtools 1.8. Peaks and coverage were obtained with macs2 version 2.1.1.20160309 (--format BAMPE --gsize 1870000000 --call-summits --bdg). To better compare ChIP tracks, ChIP samples for the same protein with a timecourse were normalized together using a custom Python script (available in the GitHub repository). Similarly, this normalization was used when mutant and control were performed in parallel (GitHub repository). ChIP quantifications were performed with multiBigWigSummary from deepTools version 3.0.0 (ref. ^64^). The cumulative H3K27ac profile of Fig. 2a was computed with a custom Python script available on the GitHub repository. To reduce the impact of variations in gastruloid growth speed for the H3K27ac and H3K27me3 datasets in Extended Data Figs. 8 and 10a, the profiles of mutant and corresponding wild-type were corrected using the timecourse experiment and the profile at the *HoxA* cluster as a guide (see GitHub repository for more details). The profiles were then averaged for the two replicates, and differences were performed using the bash script available in the GitHub repository. Motif analysis of CDX2 ChIP–seq peaks in Supplementary Fig. 5 was performed with HOMER version 4.10.

### CHi-C

Collected stembryos were pooled and fixed for 10 min in 1% formaldehyde at room temperature and stored at −80 °C until further use. Lysis was performed with TissueLyser II and metallic beads for 30 s at 3 hertz before incubation at 4° for 30 min. The rest of the protocol for the CHi-C library generation is described in ref. ^76^. The customized SureSelectXT RNA probe used for DNA capture covers the region from chr2:72240000 to 76840000 (mm9). For data analysis, TruSeq adapters and bad quality bases were removed with cutadapt^66^ version 1.16 (-a ‘AGATCGGAAGAGCACACGTCTGAACTCCAGTCAC’ -A ‘AGATCGGAAGAGCGTCGTGTAGGGAAAGAGTGTAGATCTCGGTGGTCGCCGTATCATT’ --minimum-length=15 --quality-cutoff=30). Trimmed reads were processed with HiCUP version 0.6.1 (ref. ^77^) with default parameters on mm10 or on the corresponding mutant genome. The output BAM was converted to Juicebox format, thanks to a custom Python script available at https://testtoolshed.g2.bx.psu.edu/repository/download?repository_id=be5040251cd4afb7&changeset_revision=44365a4feb3b&file_type=gz. Only pairs with both mates falling into the capture region (mm10:chr2:72402000–77000000) and with both mates with quality above 30 were kept. Filtered pairs were loaded into 2 kb and 5 kb matrices with cooler version 0.7.4 (ref. ^78^). For 48 h, 96 h and 144 h, in those wild-type samples where three replicates were available, raw matrices were summed before further processing. The matrices were balanced with cooler balance (--cis-only). For the Del(sub-TAD1) mutant, the balancing was done with the option–ignore-diags 5 instead of --ignore-diags 2 to discard the bias due to the presence of a BAC containing the region (mm10:chr2:74747769–74913171). In Fig. 3c, the histograms were produced with a Plot profile on ImageJ version 2.1.0/1.53c. Virtual 4C was computed using the same script as in ref. ^28^ inspired by the method described in ref. ^79^ (whenever multiple replicates were available, valid pair files were concatenated). The output bedgraph was then used for the quantifications.

### HiChIP

Wild-type stembryos were collected at 120 h and fixed for 10 min in 1% formaldehyde at room temperature before proceeding with the library generation. The protocol for H3K27ac HiChIP was adapted from ref. ^80^. Briefly, after lysis, DpnII digestion, biotin fill-in and blunt-end ligation (CHi-C), the samples were sonicated in a Covaris E220 instrument for 10 min (duty cycle 2% and peak incident power 105 W) and incubated overnight at 4° with magnetic beads conjugated to the H3K27ac antibody (Abcam, ab4729). The next day, beads were washed, as described in the ChIP protocol, before the elution of DNA fragments. A biotin pull-down was then performed (Dynabeads MyOne Streptavidin T1) followed by a TruSeq library preparation using fragments bound to the streptavidin beads. A qPCR was used to estimate library depth before final amplification with the Kapa HiFi library kit. Library was purified, size selected and quality assessed with a fragment analyzer before sequencing on a NextSeq 500 sequencer as paired-end, 75 bp reads. HiChIP reads were processed similarly to CHi-C, except that the adapters were removed on each read independently and bad quality pairs were conserved, pairs were not filtered for the capture region, pairs were loaded into 10 kb bins and no balancing was performed.

### Generation of movies

For Supplementary Movie 1, the H3K27ac profiles of Fig. 1c were quantified on each bin of 10 bp. Between every measured timepoint (12-h timecourse), intermediate timepoints (every hour) were generated by linear extrapolation on each bin using the previous and the next timepoints such as to smoothen the profiles and to see their dynamic. Profiles were then generated by pyGenomeTracks and combined in a film with ImageJ. For Supplementary Movie 2, the balanced, 5-kb bins, cool files used in Fig. 3a,b were used to generate intermediate cool files for each hour using a linear extrapolation on each 5-kb bin using the previous and the next timepoint. Profiles were then generated by pyGenomeTracks. Annotations (arrows, bars) were manually added, and all frames were combined in a film using ImageJ.

### Whole-mount in situ hybridization (WISH)

Stembryos were collected at 120 h and processed following a previously reported WISH procedure^34^. Briefly, they were fixed overnight in 4% PFA at 4 °C and stored in methanol at −20 °C until ready for processing. Each sample was rehydrated and prepared with Proteinase K (EuroBio) at 2.5 µg ml^−1^ for 2 min. After postfixation, stembryos were prehybridized in a blocking solution at 68 °C for 4 h before incubation overnight with specific digoxigenin-labeled RNA *Hoxd4* (ref. ^81^) and *Hoxd9* (ref. ^10^) probes at a final concentration of 200 ng ml^−1^. The next day, samples were washed and incubated with an anti-DIG antibody coupled to alkaline phosphatase (Roche, 1:3,000). Staining was performed with BM-Purple (Roche). Images of stembryos were captured with an Olympus DP74 camera mounted on an Olympus MVX10 microscope using the Olympus cellSens Standard 2.1 software. Quantification of the size of transcript domains was performed using ImageJ software (Supplementary Table 4).

### Statistics and reproducibility

Statical analyses were performed using R and Prism 9. All statistical tests used are two-sided (see details in the GitHub repository). No statistical method was used to predetermine the sample size. When quantification was needed, at least two biological replicates were produced. No data were excluded from the analyses.

### Reporting summary

Further information on research design is available in the Nature Portfolio Reporting Summary linked to this article.

## Online content

Any methods, additional references, Nature Portfolio reporting summaries, source data, extended data, supplementary information, acknowledgements, peer review information; details of author contributions and competing interests; and statements of data and code availability are available at 10.1038/s41588-023-01426-7.

## Supplementary information

Supplementary Information Supplementary Notes 1–4, Figs. 1–6 and Tables 1–4.

Reporting Summary

Supplementary Movie 1 Dynamic of colinear activation at the *HoxD* cluster.

Supplementary Movie 2 Time-lapse reconstruction of the dynamic topology at the *HoxD* locus upon transcriptional activation.

## Data Availability

All raw and processed datasets are available in the Gene Expression Omnibus (GEO) repository under accession number GSE205783.

## Code availability

All scripts necessary to reproduce figures from raw data are available at https://github.com/lldelisle/scriptsForRekaikEtAl2022 (ref. ^62^).
