# Supplementary material for: Sequential and directional insulation by conserved CTCF sites underlies the Hox timer in stembryos
Source: Nat Genet. 2023 Jun 15;55(7):1164–75. doi: 10.1038/s41588-023-01426-7 (PMC10335938; doi:10.1038/s41588-023-01426-7)
Supplement: Supplementary file 1 — Supplementary Notes 1–4, Figs. 1–6 and Tables 1–4. [file 41588_2023_1426_MOESM1_ESM.pdf]

nature genetics

Article https://doi.org/10.1038/s41588-023-01426-7

Sequential and directional insulation by conserved CTCF sites underlies the Hox timer in stembryos

In the format provided by the authors and unedited

SUPPLEMENTARY MATERIAL

Table of content

Supplementary Notes 1-4

Supplementary Movies 1-2

Supplementary Tables 1-4

SUPPLEMENTARY NOTES

Supplementary Notes 1

Paused and elongating pol II

To complement the analyses of the spreading of H3K27ac over the posterior part of the *HoxD* cluster, we ChIPed-seq the large subunit of pol II using a pan-specific antibody recognizing the C-terminal domain (CTD) of RPB1. Pol II occupancy was somewhat similar to the H3K27 acetylation profile, yet with an unexpected accumulation over *Hoxd8* and *Hoxd9* at 96h already (Supplementary Fig. 1a), which was higher than both the H3K27ac profiles and transcripts levels. Consequently, while a colinear distribution was scored when comparing the 72h, 120h and 168h time points, profiles were virtually identical between the entire 96h to 132h time window (Supplementary Fig. 1a-c). This poor colinear progression of pol II was like that reported in amphibians [1]. However, when we ChIPed pol II phosphorylated in serine 2 (Ser2-p) of the CTD domain, a modification that accompanies the transition from pausing to productive elongation, which usually covers the transcription unit with a robust enrichment in the 3' part (e.g. [2]) a different distribution was scored (Supplementary Fig. 1d).

The pol II Ser2-p profile at 96h was clearly different from that of the pan-pol II antibody and involved mainly the *Hoxd1* to *Hoxd4* region with a weak coverage of *Hoxd8*, correlating well with the H3K27ac and RNA-seq profiles at this stage (Supplementary Fig. 1d). The signals had now extended towards more posterior genes and was enriched in 3'of *Hoxd8* (Supplementary Fig. 1d, left arrow in 84h), whereas at 144h, the signal had moved in 3' of *Hoxd9* (Supplementary Fig. 1d, arrowhead). At this latter stage, the coverage of the pol II Ser2-p was eventually similar to that

seen with the pan pol II antibody. These differences between the two forms of pol II (Supplementary Fig. 1d, compare the signals with arrows in 96h to 144h) suggest that, while actively transcribed regions (pol II Ser2-p positive) were spreading from the 3’ to the 5’ parts of the cluster, Pol II remained positioned throughout, likely under a paused state.

Supplementary Figure 1

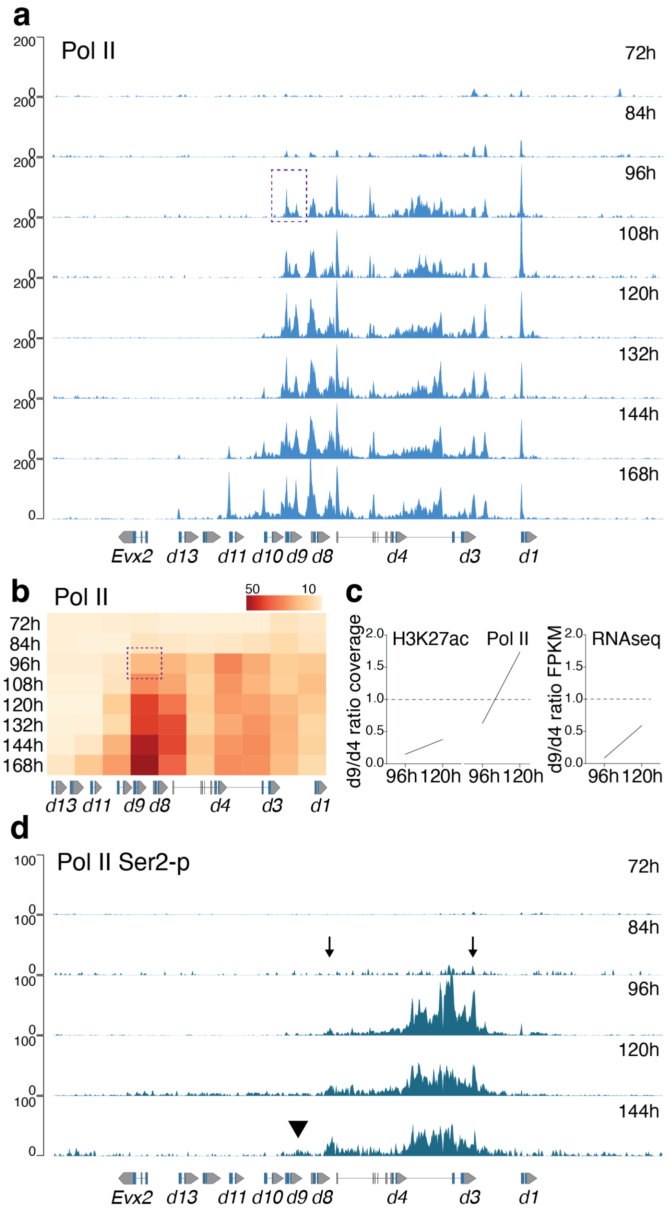

Supplementary Fig. 1. Pol II distribution during the activation of the *HoxD* cluster. a, Pol II ChIP-seq profiles over *HoxD* at different stembryonic stages. While signals are enriched over *Hoxd1* and *Hoxd3* at 72h and 84h, a surprisingly fast and large coverage in Pol II is scored at 96h, up to *Hoxd9* (dashed region). **b,** Heatmap of Pol II ChIP-seq coverage over the *HoxD* cluster divided into 10 kb bins. **c,** Computed ratio between *Hoxd9* (dashed square) and *Hoxd4* coverage (fourth bin from *Hoxd1*) for both H3K27ac and Pol II at 96h and 120h (left panel), and between their FPKM values (right panel). Comparable ratio between

H3K27 acetylation and transcription was observed, unlike with Pol II where enrichment was already observed over *Hoxd9* at 96h. **d,** ChIP-seq profiles of Pol II phosphorylated at serine 2 at five time-points. The dynamic of the elongating Pol II Ser2-p was comparable to H3K27 acetylation though with a slight delay. In **a** and **d**, genomic coordinates: chr2:74636423-74780095.

Such striking differences were also readily observed at both *HoxA* and *HoxB* clusters (Supplementary Fig. 2a, b, respectively). We concluded that elongating pol II follows a colinear dynamic first detected in anterior regions of the clusters devoid of CTCF binding sites (see below), then progressively spreading towards posterior regions similar to H3K27 acetylation, unlike paused pol II, which seems to be recruited in a rather simultaneous manner over an extended central part of the clusters including the transition from the 3' CTCF-free region towards the CTCF rich region.

Supplementary Figure 2

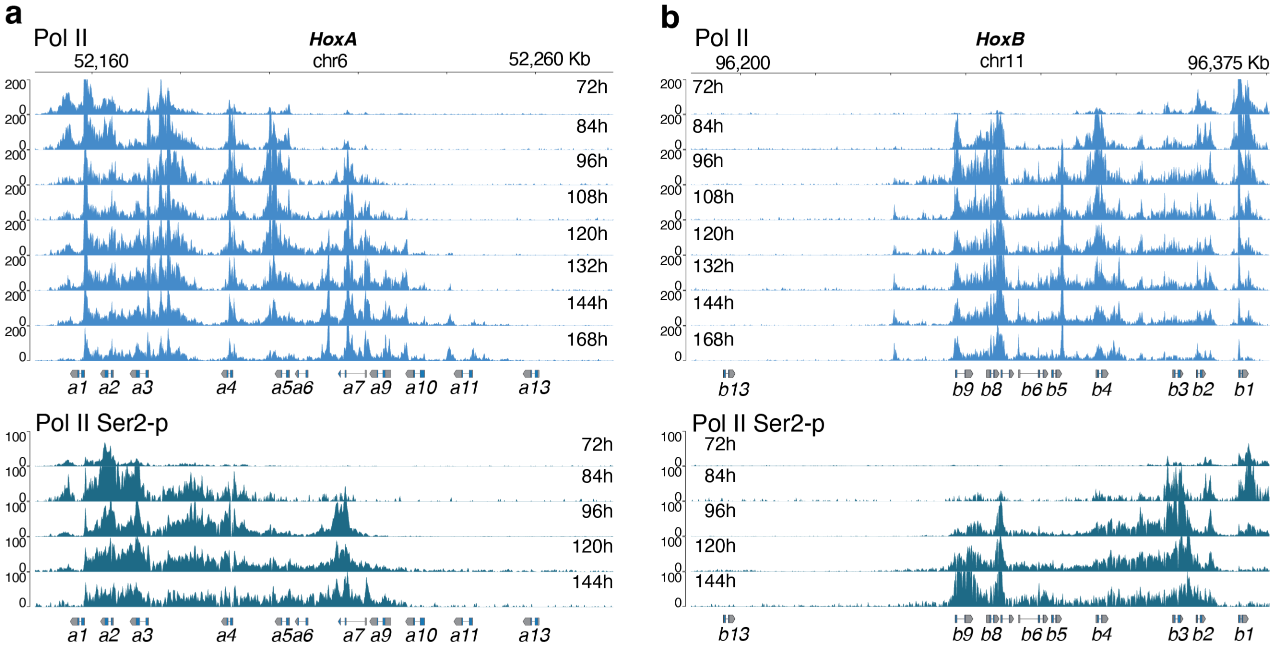

Supplementary Fig. 2. Pol II and Pol II Ser2-p profiles on *HoxA* and *HoxB* clusters. On top a time course of Pol II ChIP-seq profiles over the *HoxA* (**a**) and *HoxB* (**b**) genes clusters in stembryos from 72h to 168h, showing a rapid spreading of Pol II over the gene clusters. Bottom: The use of an antibody against Pol II with phosphorylated serine 2 in ChIP-seq profiles at various time points reveals distinct, more progressive and colinear profiles, suggesting that Pol II is initially pausing over the gene clusters before starting to elongate.

Supplementary Notes 2

Expression of CDX genes in stembryos and binding profiles

After the initial activation of anterior *Hox* genes, partly in response to *Wnt* signaling [21],[22], *Cdx* transcription factors were reported to activate more centrally located *Hox* genes [23–25], until the location of the TAD boundary (the site of inversion in the orientations of the CTCF sites) located inside the *Hox* clusters, which generally separates the *Hox12* and *Hox13* genes from all other anterior *Hox* genes. Other studies have proposed that the transcription of the latter posterior genes depends on TGFbeta signaling, in particular by *Gdf11* [26,27]. Coincidentally, the mapping of CTCF binding sites within *Hox* clusters revealed three sub-domains; an 'anterior' domain devoid of CTCF sites; a centrally located domain where series of CTCF sites are orientated towards the 3' end of the clusters, and a posterior domain where several CTCF sites display the opposite orientation [28]. A potential upstream function for CDX proteins was thus investigated.

CDX proteins [3–5] play important roles in trunk extension [6,7] and, in our stembryos, both *Cdx1* and *Cdx2* were expressed as early as 72h with their mRNAs reaching peak levels at around 84h (Supplementary Fig. 3a). Single-cell RNA-seq at 96h and 120h revealed a clear overlap between *Hoxd8*, *Hoxd9* and *Cdx1*/*Cdx2* RNAs positive cells, in particular in neuro-mesodermal progenitors (NMPs) and early presomitic mesoderm (PSM) cells (Supplementary Fig. 3b-d), suggesting that a direct regulation by CDX proteins is possible.

We produced a ChIP-seq dataset using an antibody against CDX2 and performed a HOMER *de novo* motif analysis of CDX2 peaks, which revealed homeobox-like binding motifs and scored CDX-known motifs among the top two high-scoring results (Supplementary Fig. 3e). Binding into the *HoxD* cluster was detected as early as in 72h, in several positions in the central region containing *Hoxd3* to *Hoxd9* (Supplementary Fig. 3f, orange arrows), i.e., corresponding to those genes previously proposed to be activated by CDX proteins. Three prominent peaks were scored with decreasing intensities at 120h, thus matching the timing of transcriptional activation of central genes. No binding was observed beyond *Hoxd9* at any stage analyzed, supporting CDX2 as participating to the activation of central *Hox* genes only. However, while these three peaks covered the expected *Hoxd4* to *Hoxd9* region, no evidence was found for a progressive recruitment along the cluster and the peak around *Hoxd9* appeared consistently more enriched than the two others.

Supplementary Figure 3

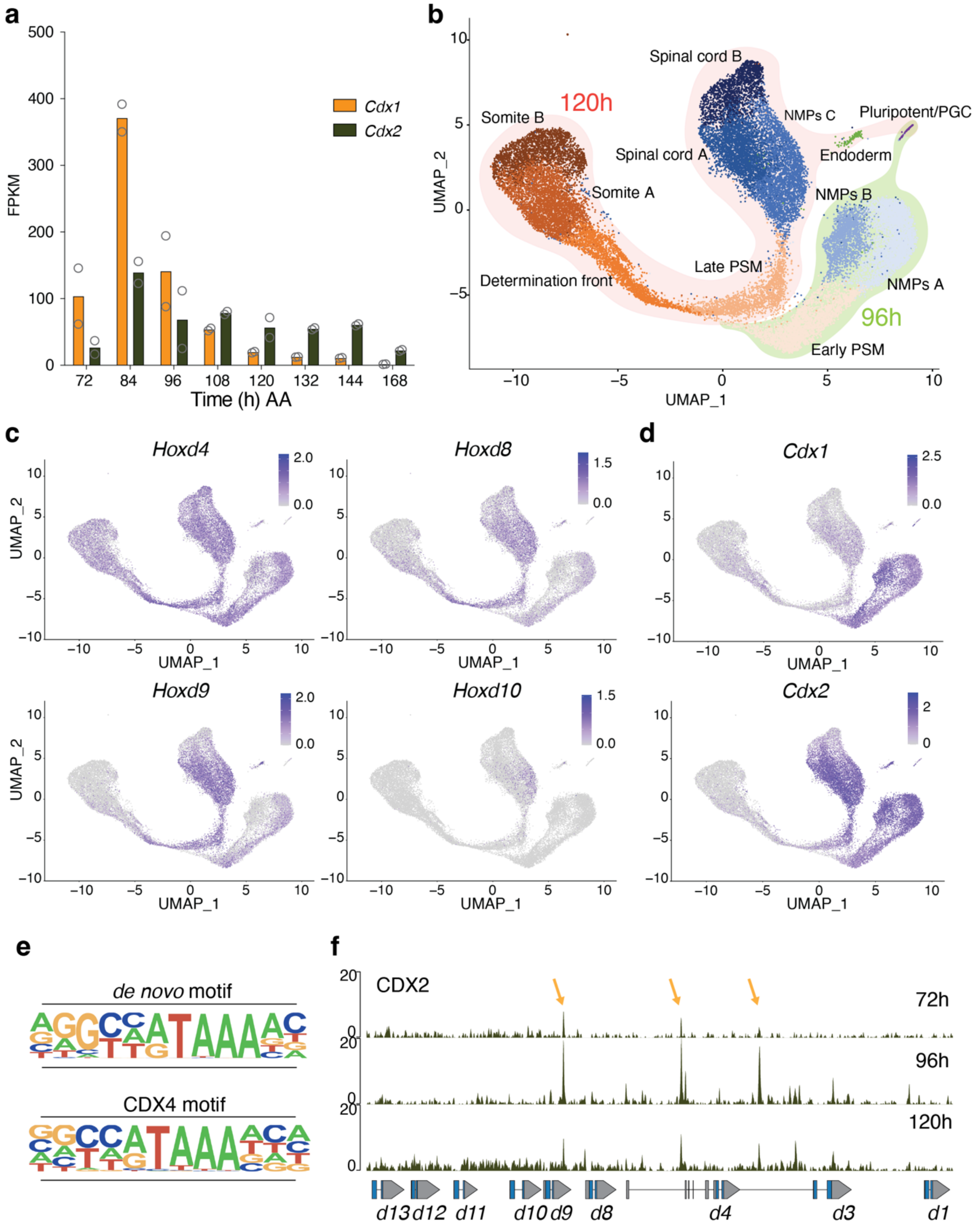

Supplementary Fig. 3. Analysis of *Cdx* genes expression and CDX binding to the *HoxD* cluster. a. FPKM values of *Cdx1* and *Cdx2* from RNA-seq produced in wild type stembryos at the indicated time points (n=2). Expression peak was reached at 84h for both genes. Values are represented as means. **b.** single-cell RNA-seq produced in wild type stembryos at 96h and 120h. UMAP projection and clustering of pooled cells showing segregation of 96h (green cluster) from 120h (red cluster) stembryonic cells. Labels indicate cellular identity for each sub-population. NMPs, neuro-mesodermal progenitors; PSM, presomitic mesoderm; PGC, Primordial Germ Cell. (**c, d.**) Single-cell expression of *Hoxd* (**c**) and *Cdx* (**d**) genes over

the UMAP clustering map. A clear overlap was observed between *Cdx* and *Hoxd8* to *Hoxd10*. **e.** HOMER motif analysis of CDX2 ChIP-seq peaks, with *de novo* motifs (top, rank:1, 49.60%, p-value: 1e-1076) and known motifs (bottom, CDX4, rank:1, 57.57%, p-value: 1e-968). **f.** CDX2 ChIP-seq profiles over the *Hoxd* genes cluster at 72h, 96h and 120h. CDX2 binding peaks within the central part of the cluster are indicated with arrows. No colinear progression was observed in CDX2 recruitment over the cluster. Genomic coordinates: chr2:74667374-74767842.

Supplementary Notes 3

Spatial expression of *Hoxd9* in mutant stembryos

In our stembryos lacking CBSs and activating prematurely the gene located immediately in 5' (posteriorly), we asked how such a premature expression would affect the spatial distribution of *Hoxd* mRNAs during stembryonic development. As an example, whole-mount *in situ* hybridization (WISH) for both *Hoxd4* and *Hoxd9* was performed on control and Del(CBS1-2) stembryos at 120h. In this mutant, the expression domain of *Hoxd9* (the gene located right after the two deleted CBSs) was clearly 'anteriorized', matching the *Hoxd4* domain (Supplementary Fig. 4). In contrast, the distribution of *Hoxd4* mRNAs remained unchanged. This result confirmed previous analyses carried out in mice lacking CBS1-2 [8] and demonstrated the tight relationships between the timing of *Hox* gene transcription and the spatial distribution of their RNAs.

Supplementary Figure 4

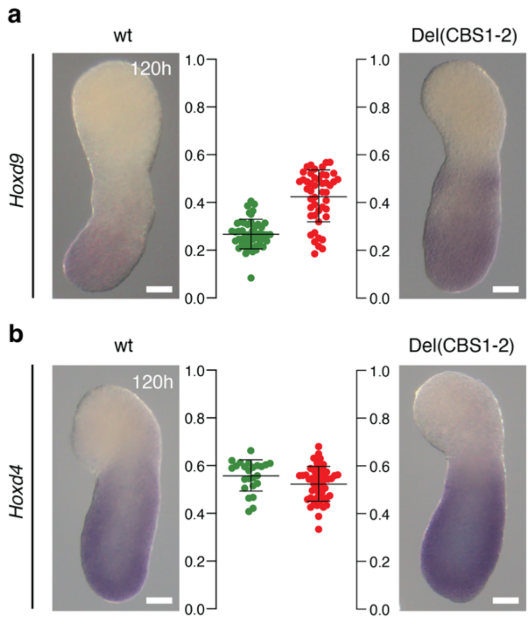

Supplementary Fig. 4. Spatial distribution of *Hoxd* genes in Del(CBS1-2) stembryos. a, *Hoxd9* and **b,** *Hoxd4* probes were used with control and Del(CBS1-2) stembryos at 120h. The size of the expression domain for each probe was quantified and normalized to the size of the whole stembryo. **a,** An 'anteriorization' in the expression domain of *Hoxd9* is observed in mutant stembryos (n=50, 47), while in (**b**) the expression domain of *Hoxd4* remains unchanged (n=24, 50). Values are represented as means ± SD. Scale bars, 100 µm.

Supplementary Notes 4

Deletions of the *Hoxd1-Hoxd4* fragment and of sub-TAD1

In addition to the single or multiple deletions of CTCF binding sites, we also looked at the importance of preferentially loading cohesin in the anterior portion of the cluster by deleting this region. This deletion removed from *Hoxd1* to *Hoxd4* included, yet CBS1 was left in place (Supplementary Fig. 5a). RNA-seq of mutant stembryos showed a marked decrease in transcripts level for *Hoxd8* and *Hoxd9*, both at 96h and 120h (Supplementary Fig. 5b). To see whether this decrease in transcription was associated with a change in the contacts established between the various intra-cluster CBSs and sub-TAD1, we produced a CHi-C dataset of Del(*Hoxd1-Hoxd4*) stembryos at 96h (Supplementary Fig. 5c). While the micro-TAD observed over the anterior region of the cluster under normal condition had expectedly disappeared along with the deletion (Supplementary Fig. 5c), a quantification of contacts between the various CBS1-5 and the CS38-40 sub-TAD1 region revealed important modifications (Supplementary Fig. 6). In 96h control stembryos, CBS2 showed a higher contact frequency with the CBSs located within the CS38-40 region, when compared to CBS1 (Supplementary Fig. 6a). In contrast, the Del(*Hoxd1-Hoxd4*) mutant stembryos showed comparable interaction levels between CS38-40 and CBS1 and 2 (Supplementary Fig. 6a). We interpret this as a reduction in cohesin complexes reaching more 'posterior' CBS, due to reduced loading in the absence of the DNA segment including early transcribed genes and targeted by NIPBL. Basal accumulation of cohesin was nevertheless sufficient to establish the expected constitutive contacts with the CS38-40 region.

We then deleted sub-TAD1 to evaluate its importance for the transcription of those central genes located in 5' of CBS1. Accordingly, the CBS-rich CS38-40 region was moved close to the anterior part of the cluster (Supplementary Fig. 5d). Changes in mRNAs level were already observed at 96h for *Hoxd8* (Supplementary Fig. 5e, top) and a substantial decrease was scored at 120h for both *Hoxd8* and *Hoxd9,* whereas anterior genes were less affected (Supplementary Fig. 5e, bottom). These results confirmed that posterior *Hoxd* genes somewhat require sub-TAD1 to be properly activated and that this may be achieved through those CBS-dependent interactions that are observed in the wild type situation. It also confirmed that the initial *Wnt*-dependent activation of anterior genes is -at least partially- independent from sub-TAD1 and thus potentially involves elements located within the cluster itself. The CHi-C profile of these mutant stembryos revealed a rewiring of contacts between the cluster and the now closely located region CS38-40

(Supplementary Fig. 5f), with a strong micro-TAD including the anterior portion of the cluster, where cohesin loading could presumably occur normally (Supplementary Fig. 6b).

Supplementary Figure 5

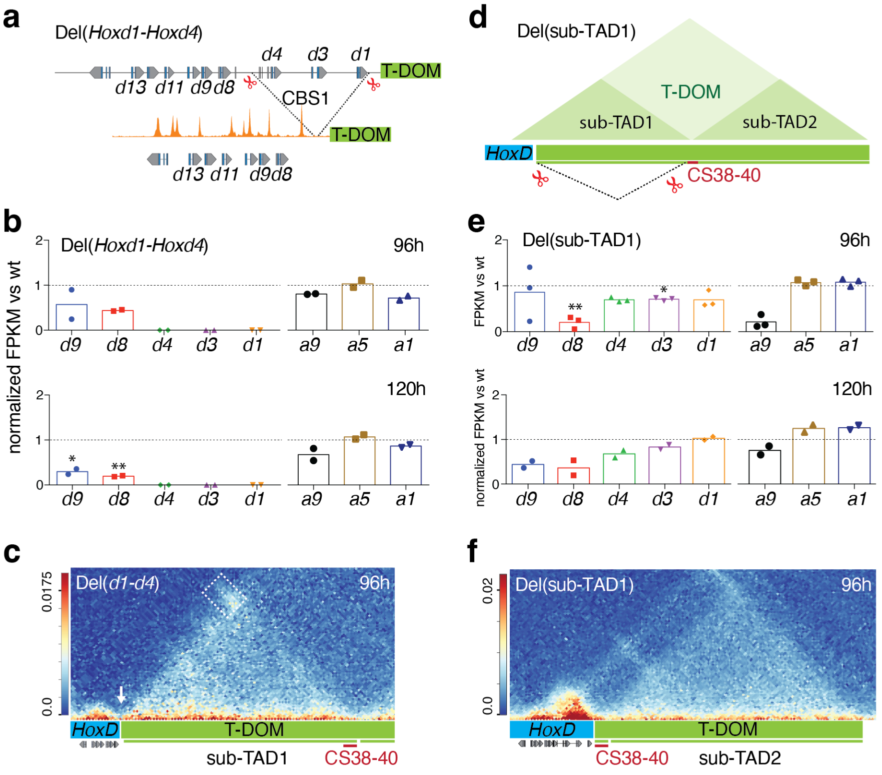

Supplementary Fig. 5. Larger deletions at the *HoxD* locus affect the timing of transcription. a, The Del(*Hoxd1-Hoxd4*) mutant, with the anterior region of the *HoxD* cluster removed. **b,** Normalized FPKM values of *Hoxd* and *Hoxa* RNAs from Del(*Hoxd1-Hoxd4*) stembryos compared to controls at 96h and 120h (n=2). **c,** CHi-C map produced from Del(*Hoxd1-Hoxd4*) stembryos at 96h, mapped onto an *in silico* reconstructed mutant genome. Bin size 5kb. The deletion breakpoint is indicated with a white arrow. Contacts between the cluster and the CS38-40 region are scored (dashed box) and quantified in Supplementary Fig. 6. Genomic coordinates: chr2:74635000-75165000 **d,** Deletion of sub-TAD1. The boundary between sub-TAD1 and sub-TAD2 (CS38-40) is left in place. **e,** FPKM (96h) and normalized FPKM (120h) values for *Hoxd* and *Hoxa* RNAs from Del(sub-TAD1) stembryos compared to control (96h: mutant (n=3), wt (n=2); 120h: n=2). In **b** and **e**, values are represented as means ± SD. P-values were determined by Welch's unequal variances t-test (* is p-value < 0.05, ** < 0.01). **f,** CHi-C map using Del(sub-TAD1) stembryos at 96h, mapped onto an *in silico* reconstructed mutant genome. Bin size 5kb. A higher contact frequency is observed between the *Hoxd* genes cluster and the CS38-40 boundary region, now relocated close by. Quantifications in Supplementary Fig. 6. Genomic coordinates: chr2:74635000-75240000.

Supplementary Figure 6

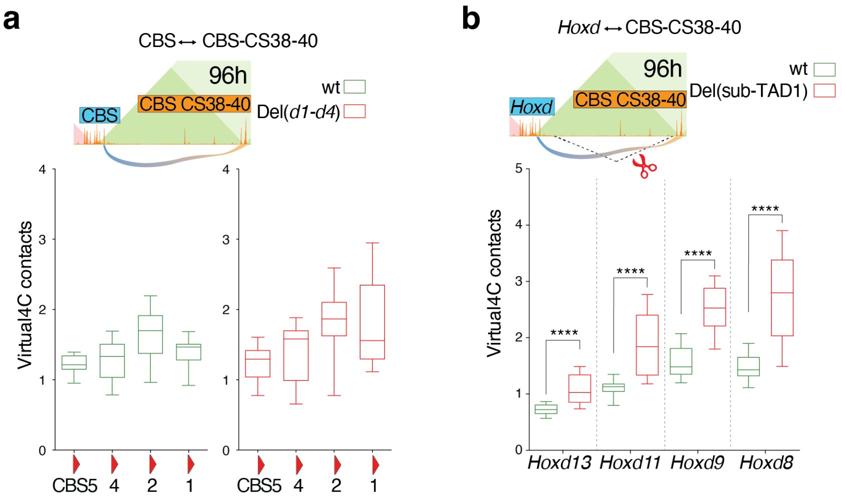

Supplementary Fig. 6. Virtual 4C contacts generated from Capture Hi-C datasets produced from either control or mutant stembryos. a, Del(*Hoxd1-Hoxd4*) and **b**, Del(sub-TAD1) stembryos were used at 96h. Data were mapped to corresponding *in-silico* reconstructed mutant genomes. **a,** Quantification of the contacts between the indicated *HoxD* cluster CBS1 to 5 (viewpoint) and the CBS of the CS38-40 region. While CBS2 is the CBS the more used in wt, in Del(CBS1-4) both CBS1 and 2 are preponderant. **b,** Contacts between the *Hoxd* gene bodies indicated below (viewpoints) and the CBS of the CS38-40 region. On top, scheme of the deleted fragment (dashed lines with scissors). An increase in contact is observed between *Hoxd* genes and the CS38-40 region in the mutant Del(sub-TAD1). In **a** and **b**, box plots with median value and 25-75% percentiles, whiskers represent minimum and maximum (wt: sum of 3 independent replicates, mutant: single replicate). In **b**, the Mann-Whitney nonparametric test was used to assess significant changes between wt and Del(sub-TAD1) mutant (**** is p-value < 0.0001).

REFERENCES

1. Kondo, M. *et al. de novo* transcription of multiple Hox cluster genes takes place simultaneously in early *Xenopus tropicalis* embryos. *Biology Open* bio.038422 (2019) doi:10.1242/bio.038422.
2. Zaborowska, J., Egloff, S. & Murphy, S. The pol II CTD: new twists in the tail. *Nat Struct Mol Biol* **23**, 771–777 (2016).
3. Amin, S. *et al.* Cdx and T Brachyury Co-activate Growth Signaling in the Embryonic Axial Progenitor Niche. *Cell Rep* **17**, 3165–3177 (2016).
4. Mazzoni, E. O. *et al.* Saltatory remodeling of Hox chromatin in response to rostrocaudal patterning signals. *Nat Neurosci* **16**, 1191–8 (2013).
5. Neijts, R. & Deschamps, J. At the base of colinear Hox gene expression: cis -features and trans -factors orchestrating the initial phase of Hox cluster activation. *Developmental Biology* **428**, 293–299 (2017).
6. Bel-Vialar, S., Itasaki, N. & Krumlauf, R. Initiating Hox gene expression: in the early chick neural tube differential sensitivity to FGF and RA signaling subdivides the HoxB genes in two distinct groups. *Development* **129**, 5103–15 (2002).

7. van den Akker, E. *et al.* Cdx1 and Cdx2 have overlapping functions in anteroposterior patterning and posterior axis elongation. *Development* **129**, 2181–93 (2002).
8. Amândio, A. R. *et al.* Sequential in *cis* mutagenesis in vivo reveals various functions for CTCF sites at the mouse *HoxD* cluster. *Genes Dev.* genesdev;gad.348934.121v1 (2021) doi:10.1101/gad.348934.121.

SUPPLEMENTARY MOVIES

Supplementary movie 1

Legend to Supplementary Movie 1: Dynamic of colinear activation at the *HoxD* cluster. Time-lapse reconstruction of H3K27ac coverage at the *HoxD* cluster between 72h and 168h, with a CTCF ChIP-seq profile (top). Each frame represents 1 h time resolution. A rapid coverage of H3K27ac is detected over the *Hoxd1* to *Hoxd4* region, followed by a slow and progressive spreading throughout the central and posterior part of the cluster.

Supplementary movie 2

Legend to Supplementary Movie 2: Time-lapse reconstruction of the dynamic topology at the *HoxD* locus upon transcriptional activation. Contacts generated from the CHi-C time-course with a 1 h per frame resolution. Within the *HoxD* cluster, the initial inactive state is visible by dense intra-cluster interactions (brown arrow). The cluster then translocates and two micro-TADs appear separated by CBS1 and corresponding to the inactive posterior region (brown arrow) and the newly activated anterior region (blue arrow). Constitutive interactions between the gene cluster and T-DOM are visible at 48h with a predominance of CBS1 (dashed white line). Soon after gene activation and the concurrent translocation of the cluster, this preferential interaction slowly shifts towards more central positions within the cluster (white line).

SUPPLEMENTARY TABLES

Supplementary Table 1

Supplementary Table 1: List of sgRNAs used to generate the various mutant ES cell lines as well as the Del(sub-TAD1) mutant mice.

Target	Forward primers	Reverse primers
Del(CBS1)_5UTR	CACCGTTCTGCCACGTCCACTGGAG	AAACCTCCAGTGGACGTGGCAGAAC
Del(CBS1)_3UTR	CACCGTGTTTCCCTCGGCGACCCAG	AAACCTGGGTCGCCGAGGGAAACAC
Del(CBS2)	CACCGGAGCAACAGCGCTCTCTAG	AAACCTAGAGAGCGCTGTTGCTCC
Del(CBS4)	CACCGTCTCCAACGCGCACTATCAC	AAACGTGATAGTGCGCGTTGGAGAC
Ins(2xCBS-d4d8)	CACCGAAATTACTCACCTATAGTG	AAACCACTATAGGTGAGTAATTTC
Del(d1-d4)_5UTR	CACCGCTGCCATTGCAACTCCCAGT	AAACACTGGGAGTTGCAATGGCAGC
Del(d1-d4)_3UTR	CACCGACTCACTCTGTAGACCACGT	AAACACGTGGTCTACAGAGTGAGTC
Del(sub-TAD1)_5UTR	CACCGATCCCGATGAATTCAGCTT	AAACAAGCTGAATTCATCGGGATC
Del(sub-TAD1)_3UTR	CACCGTAATTCAGCATCATGTTTG	AAACCAAACATGATGCTGAATTAC

Supplementary Table 2

Supplementary Table 2: List of genotyping primers used to characterize the various mutations produced in ES cells within the *HoxD* locus.

ES cell lines	Forward primers	Revers primers
Del(CBS1)	CTTGTAACGAACACCAGCACAG	GGGATTGCCTCAGACTTTCAGA
Del(CBS2)	TAAAGAATGGGGGACTTGCGGT	TTATGCCTTACTAGCACGGC
Del(CBS4)	GATTTTCACACAGCCCCCT	CATTCCATAGGTCCCCATGT
Del(d1-d4)	CTGCCCTTGAACTCACAAGAGA	AGGAAGTGGGGAGAAAGTAGGA
Ins(2xCBS-d4d8)	TGTTTCCCTCGGCGACCCAG	GGCTGGCCTCAAATTCAAATGT
Del(sub-TAD1)	GGGAAAGGAGGGCCAGAAAGTG	GTCAGGAATTCTTCCACATACTGGGG

Supplementary Table 3

Supplementary Table 3: Sanger sequencing of mutant ES cell lines produced in this study. DNA sequence was reconstructed for both alleles, using the genotyping primers shown in supplementary Table 2.

**ES cell lines**	**Allele_1**	**Allele_2**
Del(CBS1)	CTTGTAACGAACACCAGCACAGCACAT<br>TCTAAAGCTCAGGCTCAGCAGAGAGCA<br>ATCAGATAAACAAATAAGTAAATAAAT<br>AAACAAACAAACAAACAAACAAACAA<br>ACAAACTGGAGGGATTTTCCTCCTGCCT<br>CACCTAATCAAGGAGAGATGAGATGCT<br>CGCTTGGGGAAGCACAGCATGGATGGA<br>GACCCTTTCTAGCAAGGCAACTGACCT<br>CCTCCTGCTAATTTAGAACAGCGCCTGG<br>GCCCTGTGACACATTTCTGACTCTCTGA<br>AAGTCTGAGGCAATCCC	CTTGTAACGAACACCAGCACAGCACAT<br>TCTAAAGCTCAGGCTCAGCAGAGAGCA<br>ATCAGATAAACAAATAAGTAAATAAAT<br>AAACAAACAAACAAACAAACAAACAA<br>ACAAACTGGAGGGATTTTCCTCCTGCC<br>TCACCTAATCAAGGAGAGATGAGATGC<br>TCGCTTGGGGAAGCACAGCATGGATGG<br>AGACCCTTTCTAGCAAGGCAACTGACC<br>TCCTCCTGCTAATTTAGAACAGCGCCT<br>GGGCCCTGTGACACATTTCTGACTCTCT<br>GAAAGTCTGAGGCAATCCC
Del(CBS2)	TAAAGAATGGGGGACTTGCGGTGTCTG<br>GCAAAAGACAGGCTGGAAGCTTTTGGC<br>AAACACGCTACTTGCAAGTTCTTGCGA<br>GCACAAATGAGAATTTCCTCTGTATTCA<br>GGAGCCCATGGTTGCTGCACTTGACTTG<br>TTGACAGTCAAAGAGATGAAGCAGTAT<br>TAATGTCAGCGGTCCAAACCCAAGTCA<br>AACTAAAGAATTAAGATTAGAATTGGT<br>TTGAGCCAAATGAAAGGAAAATCAAAC<br>TCGGTTGGCTTGCGACCGCATTTGGCCC<br>CACTTTCTGATCTCGTAAAGCTAGCGTT<br>GTGGTGCAGGAAGGTTTTGATTAAAAA<br>TATTTACAATCTTACTTTTAATCTTAAA<br>TTAGGATAAATTTAACAAAAATAAACA<br>ACGTGAACTATTAAATTCTCAAGCCGT<br>GCTAGTAAGGCATAA	TAAAGAATGGGGGACTTGCGGTGTCTG<br>GCAAAAGACAGGCTGGAAGCTTTTGGC<br>AAACACGCTACTTGCAAGTTCTTGCGA<br>GCACAAATGAGAATTTCCTCTGTATTC<br>AGGAGCCCATGGTTGCTGCACTTGACT<br>TGTTGACAGTCAAAGAGATGAAGCAGT<br>ATTAATGTCAGCGGTCCAAACCCAAGT<br>CAAACTAAAGAATTAAGATTAGAATTG<br>GTTTGAGCCAAATGAAAGGAAAATCA<br>AACTCGGTTGGCTTGCGACCGCATTTG<br>GCCACTTTCTGATCTCGTAAAGCTAGC<br>GTTGTGGTGCAGGAAGGTTTTGATTAA<br>AAATATTTACAATCTTACTTTTAATCTT<br>AAATTAGGATAAATTTAACAAAAATAA<br>ACAACGTGAACTATTAAATTCTCAAGC<br>CGTGCTAGTAAGGCATAA
Del(CBS1-2)	TAAAGAATGGGGGACTTGCGGTGTCTG<br>GCAAAAGACAGGCTGGAAGCTTTTGGC<br>AAACACGCTACTTGCAAGTTCTTGCGA<br>GCACAAATGAGAATTTCCTCTGTATTCA<br>GGAGCCCATGGTTGCTGCACTTGACTTG<br>TTGACAGTCAAAGAGATGAAGCAGTAT<br>TAATGTCAGCGGTCCAAACCCAAGTCA<br>AACTAAAGAATTAAGATTAGAATTGGT<br>TTGAGCCAAATGAAAGGAAAATCAAAC<br>TCGGTTGGCTTGCGACCGCATTTGGCTG<br>TTGCTCCACTTTCTGATCTCGTAAAGCT<br>AGCGTTGTGGTGCAGGAAGGTTTTGAT<br>TAAAAATATTTACAATCTTACTTTTAAT<br>CTTAAATTAGGATAAATTTAACAAAAA<br>TAAACAACGTGAACTATTAAATTCTCA<br>AGCCGTGCTAGTAAGGCATAA	TAAAGAATGGGGGACTTGCGGTGTCTG<br>GCAAAAGACAGGCTGGAAGCTTTTGGC<br>AAACACGCTACTTGCAAGTTCTTGCGA<br>GCACAAATGAGAATTTCCTCTGTATTC<br>AGGAGCCCATGGTTGCTGCACTTGACT<br>TGTTGACAGTCAAAGAGATGAAGCAGT<br>ATTAATGTCAGCGGTCCAAACCCAAGT<br>CAAACTAAAGAATTAAGATTAGAATTG<br>GTTTGAGCCAAATGAAAGGAAAATCA<br>AACTCGGTTGGCTTGCGACCGCATTGC<br>TCCACTTTCTGATCTCGTAAAGCTAGC<br>GTTGTGGTGCAGGAAGGTTTTGATTAA<br>AAATATTTACAATCTTACTTTTAATCTT<br>AAATTAGGATAAATTTAACAAAAATAA<br>ACAACGTGAACTATTAAATTCTCAAGC<br>CGTGCTAGTAAGGCATAA

Del(CBS4)	GATTTTCACACAGCCCCCTTTGGAACCC<br>TAGGGAGGGATGTTTCCGAACTAGAGA<br>AATCGGACTCACCTTCCTGGCTGCCCAG<br>TACTAAATTAACCAGCCCGCGCCCCTAT<br>CCCTTCCTGGGAACCGGCCCTCTTGGGC<br>GCGTTCTGCCTGGGTATGTGCAGGGAG<br>AGCGCTTCTGTGAGCAGGCGCCACAGC<br>GCTCTGACTTTCCTCAGTACGCGTGTCC<br>TCCCGGAGGCCCCTCGAATCAAGCCTTT<br>CCTTCCGCTGGCGGGGCCCGGTGGCCG<br>GGTGGGGGACTCGAGTGGGGCAGGCCG<br>GCCGGCCAAACAAGCCCCGGGAGACAG<br>GCTGGCCGGCAGCAGAGCTCTGGGCCG<br>GTTGTTGTCCGTGCTTACGCGGCCAGCC<br>GGCCGGGCTGGGGAACACGTGACCTGC<br>AAGGAGGCTGCGGCTCAAGGCCATTTT<br>CAAATCTCATTGGCTTGGTTGTCATGTG<br>GTCGGCAGAGGCATCCACAATTACACC<br>GGGAATGTTTTCCTAGCGATGTCAGCCT<br>ACAAAGGACACAATCTCTCTTCTTCAA<br>ATTCCTCCCCAAAATGTCCTTTCCCAAC<br>AGCTCTCCTGCTGCTAATACTTTTTTAG<br>TAGATTCCTTGATCAGTGCCTGCAGGA<br>GTGACAGTTTTTATTCGAGCAGCGCCA<br>GCATGTACATGCCACCACCTAGCGCAG<br>ACATGGGGACCTATGGAATG	GATTTTCACACAGCCCCCTTTGGAACC<br>CTAGGGAGGGATGTTTCCGAACTAGAG<br>AAATCGGACTCACCTTCCTGGCTGCCC<br>AGTACTAAATTAACCAGCCCGCGCCCC<br>TATCCCTTCCTGGGAACCGGCCCTCTTG<br>GGCGCGTTCTGCCTGGGTATGTGCAGG<br>GAGAGCGCTTCTGTGAGCAGGCGCCAC<br>AGCGCTCTGACTTTCCTCAGTACGCGT<br>GTCCTCCCGGAGGCCCCTCGAATCAAG<br>CCTTTCCTTCCGCTGGCGGGGCCCGGT<br>GGCCGGGTGGGGGACTCGAGTGGGGC<br>AGGCCGGCCGGCCAAACAAGCCCCGG<br>GAGACAGGCTGGCCGGCAGCAGAGCT<br>CTGGGCCGGTTGTCTCCAACGCGCACT<br>ATCGCTGTCGCTGTTGTCCGTGCTTACG<br>CGGCCAGCCGGCCGGGCTGGGGAACA<br>CGTGACCTGCAAGGAGGCTGCGGCTCA<br>AGGCCATTTTCAAATCTCATTGGCTTG<br>GTTGTCATGTGGTCGGCAGAGGCATCC<br>ACAATTACACCGGGAATGTTTTCCTAG<br>CGATGTCAGCCTACAAAGGACACAATC<br>TCTCTTCTTCAAATTCCTCCCCAAAATG<br>TCCTTTCCCAACAGCTCTCCTGCTGCTA<br>ATACTTTTTTAGTAGATTCCTTGATCAG<br>TGCCTGCAGGAGTGACAGTTTTTATTC<br>GAGCAGCGCCAGCATGTACATGCCACC<br>ACCTAGCGCAGACATGGGGACCTATGG<br>AATG
Del(d1-d4)	CTGCCCTTGAACTCACAAGAGATTCAC<br>ATGCCTCTGCCTCCCAAGTGCTGGGATG<br>AAGGCCTGCACCACCACTGCCCAGCTT<br>CCTTACAGATGCTGCAGGAAGGCTACC<br>CTCTTTTCTCCCTTCCTTGTCGACTGGA<br>ACAACTACCAACATTCAAAGTCCTTGG<br>GAGCCAGAATGTTGGCTGGGGCTCAGA<br>GTTTGGGCTCTTGTCTAGCACTCAGAAA<br>TGCTCCTGCCTCTGCCTCCCAAGTGCTT<br>GAATTAAAGGTGTGAGCCAAAACCACT<br>GGCTACTTGTCTGTTTTTAACAGTGGAA<br>TATTTTTAAATTAATTTCTTAGAAGAGG<br>TACAACTTGAAATCTGGCCAGAGGGTA<br>GATTACAAATAAACAAAACACAAAACC<br>CTCTACTCTTATTTTTCACTTAGTCTCCC<br>TTCTGCAGAAGGTGCCCACAGTCAGCA<br>GGGCTCCTGGGCTACCTCGCAGTTACAT<br>AAACGGGGAAGAAAAGTGCAATAGAA<br>ACACATATAAGCAAGTGGGTACTTTAT<br>CAAGTTAGGATGTTTCTCCTACTTTCTC<br>CCCACTTCCT	CTGCCCTTGAACTCACAAGAGATTCAC<br>ATGCCTCTGCCTCCCAAGTGCTGGGAT<br>GAAGGCCTGCACCACCACTGCCCAGCT<br>TCCTTACAGATGCTGCAGGAAGGCTAC<br>CCTCTTTTCTCCCTTCCTTGTCGACTGG<br>AACAACTACCAACATTCAAAGTCCTTG<br>GGAGCCAGAATGTTGGCTGGGGCTCAG<br>AGTTTGGGCTCTTGTCTAGCACTCCCA<br>AGTTGGCCTTGAACTCACAGAAATGCT<br>CCTGCCTCTGCCTCCCAAGTGCTTGAAT<br>TAAAGGTGTGAGCCAAAACCACTGGCT<br>ACTTGTCTGTTTTTAACAGTGGAATATT<br>TTTAAATTAATTTCTTAGAAGAGGTAC<br>AACTTGAAATCTGGCCAGAGGGTAGAT<br>TACAAATAAACAAAACACAAAACCCTC<br>TACTCTTATTTTTCACTTAGTCTCCCTT<br>CTGCAGAAGGTGCCCACAGTCAGCAGG<br>GCTCCTGGGCTACCTCGCAGTTACATA<br>AACGGGGAAGAAAAGTGCAATAGAAA<br>CACATATAAGCAAGTGGGTACTTTATC<br>AAGTTAGGATGTTTCTCCTACTTTCTCC<br>CCACTTCCT

Ins(2xCBS-d4d8)	TGTTTCCCTCGGCGACCCAGAGGGGGC<br>GTTATTTCCTCATTCTGTACTCTAGGAA<br>CAGGCAACCAGATTTTGTGATAGAGCA<br>AGCTGTTCCTAAACCTCAGGGCCCCAG<br>TGAGAAGCAGCACCCCATGAACAGCTT<br>CAGTTCTTGCCTTTCACTTGTCCCAAGT<br>TCCTAGTACCCAGGACAGGCAGCTTGG<br>GGAAGCACAGCATGGATGGAGACCCTT<br>TCTAGCAAGGCAACTGACCTCCTCCTGC<br>TAATTTAGAACAGCGCCTGGGCCCTGT<br>GACACATTTCTGACTCTCTGAAAGTCTG<br>AGGCAATCCCACCTGCACCCCATAGCT<br>TGCGAATCATGCTGGTGTGTGGACTTCT<br>GTCCCCAACACTCCTGCCTCACCACTGT<br>GCTCGTCGCAGGCCTACTGTGTGGTTCG<br>CCGGCTGGTCTCCGTTCAAAGGTGACA<br>ACTCACCCAGCTTCACTGATCCTCAGTT<br>GACCCCTAGCAATGTCCCCCTCTTTGTT<br>AATTTAGGGCAATGATTCTCAGAGGTC<br>TGTGTGAGCCCAAATGTCTGGATAAAT<br>GAGGTTTCTAGGTTCCACACCAAACAC<br>CCTGAATTCAAATGTCAAGAAGCAGTG<br>TCCAGGCATCCTGAAAACACTCTCCCC<br>AAGGCAATGAAGATATGTACATAGGTT<br>GGAGAGCCATGGGTTAAGGGTGTCTCC<br>TGCTTAGCTCTTTATTCTCCTGGTCCTG<br>AGTTTCTTCTTTGTCTACACTGGGCATA<br>CCCGATTTTCCTCCTGCCTCACCTAATC<br>AAGGAGAGATGAGATGCTCGCTTCTGG<br>GTCACAGGCTTGTTGTCTGAGCTGGGG<br>GTGGGGCGGTAAGAGGTAGCCTCAAGG<br>ACTTTTGGACTCCTTTTTGCCTCTCCAG<br>TGGACGTGGCAGAATCAACTTCTGATG<br>TCCCAGGAAGGAAGTGCCTAGTCTAGG<br>TTTTCTAGGGCTGGTGGTGTCCCTTTTC<br>GGTTGTCTGGAGCTTTGTCCAGGAGGA<br>GGCGCTGTAGGACAAGCGTCTCGCTGG<br>CTTCGCCGATGCCGCCCATGCCCTGGGT<br>GGGAGGGGAATGGGAGGACACAAGAG<br>CCACCCGTTCTTCTGCCCCGCCTTCCAC<br>CTCAGCTCGCTCAGCGCTCAGCGTGCGT<br>GGCATCAGACAAGGGTGGGTGGTGTGA<br>TCACTTGTCCGCCAGCTGCCCTCAACCA<br>GGCATCTCTGAGGTCGAGATGAGATGC<br>TCGCTTCTGGGTCACAGGCTTGTTGTCT<br>GAGCTGGGGGTGGGGCGGTAAGAGGTA<br>GCCTCAAGGACTTTTGGACTCCTTTTTG<br>CCTCTCCAGTGGACGTGGCAGAATCAA<br>CTTCTGATGTCCCAGGAAGGAAGTGCC<br>TAGTCTAGGTTTTCTAGGGCTGGTGGTG<br>TCCCTTTTCGGTTGTCTGGAGCTTTGTC<br>CAGGAGGAGGCGCTGTAGGACAAGCGT<br>CTCGCTGGCTTCGCCGATGCCGCCCATG<br>CCCTGGGTGGGAGGGGAATGGGAGGAC<br>ACAAGAGCCACCCGTTCTTCTGCCCCGC<br>CTTCCACCTCAGCTCGCTCAGCGCTCAG<br>CGTGCGTGGCATCAGACAAGGGTGGGT	TGTTTCCCTCGGCGACCCAGAGGGGGC<br>GTTATTTCCTCATTCTGTACTCTAGGAA<br>CAGGCAACCAGATTTTGTGATAGAGCA<br>AGCTGTTCCTAAACCTCAGGGCCCCAG<br>TGAGAAGCAGCACCCCATGAACAGCTT<br>CAGTTCTTGCCTTTCACTTGTCCCAAGT<br>TCCTAGTACCCAGGACAGGCAGCTTGG<br>GGAAGCACAGCATGGATGGAGACCCTT<br>TCTAGCAAGGCAACTGACCTCCTCCTG<br>CTAATTTAGAACAGCGCCTGGGCCCTG<br>TGACACATTTCTGACTCTCTGAAAGTCT<br>GAGGCAATCCCACCTGCACCCCATAGC<br>TTGCGAATCATGCTGGTGTGTGGACTT<br>CTGTCCCCAACACTCCTGCCTCACCACT<br>GTGCTCGTCGCAGGCCTACTGTGTGGT<br>TCGCCGGCTGGTCTCCGTTCAAAGGTG<br>ACAACTCACCCAGCTTCACTGATCCTC<br>AGTTGACCCCTAGCAATGTCCCCCTCTT<br>TGTTAATTTAGGGCAATGATTCTCAGA<br>GGTCTGTGTGAGCCCAAATGTCTGGAT<br>AAATGAGGTTTCTAGGTTCCACACCAA<br>ACACCCTGAATTCAAATGTCAAGAAGC<br>AGTGTCCAGGCATCCTGAAAACACTCT<br>CCCCAAGGCAATGAAGATATGTACATA<br>GGTTGGAGAGCCATGGGTTAAGGGTGT<br>CTCCTGCTTAGCTCTTTATTCTCCTGGT<br>CCTGAGTTTCTTCTTTGTCTACACTGGG<br>CATACCCGATTTTCCTCCTGCCTCACCT<br>AATCAAGGAGAGATGAGATGCTCGCTT<br>CTGGGTCACAGGCTTGTTGTCTGAGCT<br>GGGGGTGGGGCGGTAAGAGGTAGCCT<br>CAAGGACTTTTGGACTCCTTTTTGCCTC<br>TCCAGTGGACGTGGCAGAATCAACTTC<br>TGATGTCCCAGGAAGGAAGTGCCTAGT<br>CTAGGTTTTCTAGGGCTGGTGGTGTCC<br>CTTTTCGGTTGTCTGGAGCTTTGTCCAG<br>GAGGAGGCGCTGTAGGACAAGCGTCTC<br>GCTGGCTTCGCCGATGCCGCCCATGCC<br>CTGGGTGGGAGGGGAATGGGAGGACA<br>CAAGAGCCACCCGTTCTTCTGCCCCGC<br>CTTCCACCTCAGCTCGCTCAGCGCTCA<br>GCGTGCGTGGCATCAGACAAGGGTGG<br>GTGGTGTGATCACTTGTCCGCCAGCTG<br>CCCTCAACCAGGCATCTCTGAGGTCGA<br>GATGAGATGCTCGCTTCTGGGTCACAG<br>GCTTGTTGTCTGAGCTGGGGGTGGGGC<br>GGTAAGAGGTAGCCTCAAGGACTTTTG<br>GACTCCTTTTTGCCTCTCCAGTGGACGT<br>GGCAGAATCAACTTCTGATGTCCCAGG<br>AAGGAAGTGCCTAGTCTAGGTTTTCTA<br>GGGCTGGTGGTGTCCCTTTTCGGTTGTC<br>TGGAGCTTTGTCCAGGAGGAGGCGCTG<br>TAGGACAAGCGTCTCGCTGGCTTCGCC<br>GATGCCGCCCATGCCCTGGGTGGGAGG<br>GGAATGGGAGGACACAAGAGCCACCC<br>GTTCTTCTGCCCCGCCTTCCACCTCAGC<br>TCGCTCAGCGCTCAGCGTGCGTGGCAT

	GGTGTGATCACTTGTCCGCCAGCTGCCC<br>TCAACCAGGCATCTCTGAGGTCTTTGGA<br>ACCTGAAAACTGATGCCGGGGTGACTG<br>GGTTTTGCAAGTGCCAAGAGCAGGTTC<br>TAAACTAAGTTCAGGCTTAATTGGGTTC<br>AAATTCAAACCCTACACTTGCTTGATGC<br>TTGGCCTTCAGTGAGCAGGACAATGTC<br>TTTGAGCTCATTTCCTTCTTTGAAAGTA<br>GGAATAGAAAGAGTATCATCAAGAGTT<br>CTTATGAGGATCTTACTTTTTTAGAGAC<br>AGGATGTCATTATGTTACCAGACTGGC<br>CTTGGACTTATGGCAATCCTTATGGCAG<br>TCCCCAGGGTACCATGGGGTGTAAGCC<br>CACCACACATGTCTTAGTTTTTACTTCC<br>ATGTTACTTTTATATTGAGCACAATCCT<br>AACTGTGTTGAGTATACTTATTAGCTAA<br>GTACTTAAAACAGTGCTTCGGCACATA<br>AACATTTAACACCATTAATGGTATTGGC<br>CCCGCTTTCTCATAAGTAAGACCTTGGA<br>TTTTCCAGGCAAAAGAAACTAGCTTATT<br>TTCTCACTAAATCCTTGAGTTCTATACA<br>GGATAAAAGAGACCTAAACTGGACATG<br>ATGGTTCATGCCTATAGTCCCCACACTT<br>AGGAAGCGGCAGCAGAAGGATCAACA<br>TTTGAATTTGAGGCCAGCC	CAGACAAGGGTGGGTGGTGTGATCACT<br>TGTCCGCCAGCTGCCCTCAACCAGGCA<br>TCTCTGAGGTCTTTGGAACCTGAAAAC<br>TGATGCCGGGGTGACTGGGTTTTGCAA<br>GTGCCAAGAGCAGGTTCTAAACTAAGT<br>TCAGGCTTAATTGGGTTCAAATTCAAA<br>CCCTACACTTGCTTGATGCTTGGCCTTC<br>AGTGAGCAGGACAATGTCTTTGAGCTC<br>ATTTCCTTCTTTGAAAGTAGGAATAGA<br>AAGAGTATCATCAAGAGTTCTTATGAG<br>GATCTTACTTTTTTAGAGACAGGATGT<br>CATTATGTTACCAGACTGGCCTTGGAC<br>TTATGGCAATCCTTATGGCAGTCCCCA<br>GGGTACCATGGGGTGTAAGCCCACCAC<br>ACATGTCTTAGTTTTTACTTCCATGTTA<br>CTTTTATATTGAGCACAATCCTAACTGT<br>GTTGAGTATACTTATTAGCTAAGTACTT<br>AAAACAGTGCTTCGGCACATAAACATT<br>TAACACCATTAATGGTATTGGCCCCGC<br>TTTCTCATAAGTAAGACCTTGGATTTTC<br>CAGGCAAAAGAAACTAGCTTATTTTCT<br>CACTAAATCCTTGAGTTCTATACAGGA<br>TAAAAGAGACCTAAACTGGACATGATG<br>GTTCATGCCTATAGTCCCCACACTTAG<br>GAAGCGGCAGCAGAAGGATCAACATT<br>TGAATTTGAGGCCAGCC
Del(sub-TAD1)	GGGAAAGGAGGGCCAGAAAGTGGTCA<br>TTTGAACTTGCAACAGTTAACAAAACA<br>AGCCACAGGCAATTGCAACGCATTTTT<br>AGGGGACATCAAACTGTTGAGTACATC<br>CTATCATCAGGAGCCTGCACCTATGCA<br>GTTTGAAAGGGCTCCATTAAGAAGACG<br>AATACAAAACTATGGCTGAGAGATAAG<br>GCTTTCATGGGACCAGAAAGGATCCCG<br>ATGAATTCAGCATCATGTTTGTCCCAGT<br>TTAATGAGGCATGCTGGGTGGGTATTA<br>ACTGGTCCTAACCAACCCTACTCTAAGT<br>AGATGGCAGCCTTTTCTACTGGAAACC<br>CCGTCAACTAAGTTTATATCAGATTGAA<br>GTTACTATGTAAAGACTCTCTGGGTAAT<br>TTTTTACCCTTCGCACCAATCTGGTTTA<br>AAATTACAGTTCCTCGGAGTGTTTAAG<br>GAACTCTGTGAGACTGTTTAAAAGTCTC<br>TGAGCACTAAAACTCAAAGAAGGCAGA<br>AACTCAGATGGAGCAGCCTGGGGAAAT<br>GAGTGGATAGGTTATGTAAGATTTAAA<br>AACAGTGGAAGCCCAACAGCCAAATGC<br>ATGAGACTCTCAAATCATTTTTCCCAAT<br>TTAAAATGCAAAGTCTAGGTGGATTTCT<br>GGCCAATTCTCTAAAAACCTTAGAGCT<br>GAGAATGTGACATTTAAGGCTTATATC<br>AATCACTACTAAACTCCGGAGGGTTTA<br>TTAAATTAACACACTGAGGATGAAGGT<br>GGTGAATGAAGGGCAGAAAATCTTATT<br>GCAAGGCCTCTACACCCCCAGTATGTG<br>GAAGAATTCCTGAC	GGGAAAGGAGGGCCAGAAAGTGGTCA<br>TTTGAACTTGCAACAGTTAACAAAACA<br>AGCCACAGGCAATTGCAACGCATTTTT<br>AGGGGACATCAAACTGTTGAGTACATC<br>CTATCATCAGGAGCCTGCACCTATGCA<br>GTTTGAAAGGGCTCCATTAAGAAGACG<br>AATACAAAACTATGGCTGAGAGATAA<br>GGCTTTCATGGGACCAGAAAGGATCCC<br>GATGAATTCAGCATCATGTTTGTCCCA<br>GTTTAATGAGGCATGCTGGGTGGGTAT<br>TAACTGGTCCTAACCAACCCTACTCTA<br>AGTAGATGGCAGCCTTTTCTACTGGAA<br>ACCCCGTCAACTAAGTTTATATCAGAT<br>TGAAGTTACTATGTAAAGACTCTCTGG<br>GTAATTTTTTACCCTTCGCACCAATCTG<br>GTTTAAAATTACAGTTCCTCGGAGTGT<br>TTAAGGAACTCTGTGAGACTGTTTAAA<br>AGTCTCTGAGCACTAAAACTCAAAGAA<br>GGCAGAAACTCAGATGGAGCAGCCTG<br>GGGAAATGAGTGGATAGGTTATGTAAG<br>ATTTAAAAACAGTGGAAGCCCAACAGC<br>CAAATGCATGAGACTCTCAAATCATTT<br>TTCCCAATTTAAAATGCAAAGTCTAGG<br>TGGATTTCTGGCCAATTCTCTAAAAAC<br>CTTAGAGCTGAGAATGTGACATTTAAG<br>GCTTATATCAATCACTACTAAACTCCG<br>GAGGGTTTATTAAATTAACACACTGAG<br>GATGAAGGTGGTGAATGAAGGGCAGA<br>AAATCTTATTGCAAGGCCTCTACACCC<br>CCAGTATGTGGAAGAATTCCTGAC

Supplementary Table 4

Supplementary Table 4: Quantification table of WISH experiment shown in Supplementary Fig. 4, top.

Hoxd4						*Hoxd9*					
wt			Del(CBS1-2)			wt			Del(CBS1-2)		
domain size	stembryo length	ratio	domain size	stembryo length	ratio	domain size	stembryo length	ratio	domain size	stembryo length	ratio
2.113	3.499	0.60	1.726	3.699	0.47	0.868	2.246	0.39	1.209	3.786	0.32
1.377	3.375	0.41	1.988	3.692	0.54	0.69	3	0.23	1.695	3.272	0.52
2.31	3.77	0.61	1.653	3.807	0.43	0.908	2.311	0.39	1.711	3.564	0.48
2.239	3.796	0.59	1.878	3.448	0.54	0.87	3.08	0.28	1.955	3.883	0.50
2.091	4.134	0.51	1.296	3.869	0.33	0.778	2.934	0.27	1.592	3.545	0.45
2.248	4.281	0.53	1.809	3.703	0.49	0.847	2.7	0.31	1.378	3.981	0.35
1.815	3.917	0.46	1.977	4.159	0.48	0.649	3.035	0.21	1.463	3.84	0.38
2.199	3.939	0.56	2.008	3.487	0.58	0.715	3.044	0.23	1.021	4.067	0.25
1.572	3.734	0.42	1.737	3.777	0.46	0.702	2.633	0.27	1.973	3.835	0.51
2.346	3.777	0.62	2.179	3.978	0.55	0.77	2.599	0.30	1.313	3.861	0.34
2.388	3.989	0.60	1.903	3.383	0.56	0.547	2.935	0.19	1.633	3.93	0.42
2.723	4.106	0.66	1.943	3.504	0.55	0.73	3.056	0.24	0.971	3.973	0.24
2.327	3.901	0.60	1.716	3.453	0.50	0.757	2.688	0.28	1.555	3.685	0.42
1.858	3.975	0.47	1.761	4.127	0.43	0.619	3.198	0.19	1.775	3.673	0.48
2.23	3.845	0.58	2.006	3.368	0.60	0.916	3.412	0.27	1.984	3.671	0.54
2.071	3.397	0.61	2.128	3.362	0.63	0.795	2.27	0.35	2.065	3.635	0.57
1.932	3.746	0.52	1.813	3.78	0.48	0.711	2.402	0.30	1.622	3.385	0.48
1.994	3.671	0.54	1.728	3.083	0.56	0.633	2.588	0.24	1.8	3.815	0.47
2.129	3.575	0.60	1.678	3.261	0.51	0.675	3.199	0.21	1.482	3.776	0.39
2.212	4.031	0.55	2.231	3.634	0.61	0.706	2.63	0.27	1.617	3.653	0.44
1.997	3.662	0.55	1.734	3.964	0.44	0.63	2.976	0.21	1.308	3.798	0.34
1.992	3.317	0.60	1.862	3.548	0.52	0.797	2.853	0.28	1.714	3.119	0.55
2.352	3.969	0.59	1.706	3.737	0.46	0.57	2.629	0.22	0.943	4.012	0.24
2.363	3.864	0.61	2.015	3.456	0.58	0.835	2.628	0.32	1.878	3.851	0.49
			1.753	4.025	0.44	0.789	2.981	0.26	1.92	3.635	0.53
			1.644	3.754	0.44	0.877	2.841	0.31	0.832	3.835	0.22
			1.732	4.057	0.43	0.872	2.785	0.31	1.582	3.191	0.50
			1.734	3.76	0.46	0.721	2.74	0.26	1.668	3.536	0.47
			1.458	3.754	0.39	0.663	2.659	0.25	1.743	3.131	0.56
			1.599	3.438	0.47	0.62	2.498	0.25	0.744	4.011	0.19

			2.104	3.236	0.65	0.785	2.956	0.27	1.718	3.756	0.46
			1.741	3.45	0.50	0.858	2.703	0.32	1.035	3.78	0.27
			1.723	2.844	0.61	0.62	3.065	0.20	1.771	3.993	0.44
			2.018	3.526	0.57	0.779	3.151	0.25	1.764	3.622	0.49
			2.125	3.798	0.56	0.848	3.203	0.26	1.717	3.447	0.50
			1.997	3.163	0.63	0.728	2.634	0.28	1.393	3.806	0.37
			1.925	3.444	0.56	0.64	3.072	0.21	1.453	3.893	0.37
			1.706	2.895	0.59	0.593	2.617	0.23	1.787	3.67	0.49
			1.461	2.882	0.51	0.694	2.814	0.25	1.775	3.227	0.55
			2.119	3.109	0.68	0.669	2.799	0.24	0.957	3.63	0.26
			1.563	2.859	0.55	0.906	3.237	0.28	1.728	3.302	0.52
			2.069	3.802	0.54	0.594	2.455	0.24	1.661	3.344	0.50
			1.968	3.425	0.57	0.679	3.375	0.20	0.799	3.868	0.21
			2.022	3.645	0.55	0.896	2.911	0.31	1.881	3.605	0.52
			1.579	3.384	0.47	0.875	2.459	0.36	1.443	3.804	0.38
			2.219	3.797	0.58	1.037	2.56	0.41	1.629	2.865	0.57
			2.012	3.581	0.56	0.913	2.503	0.36	1.549	3.793	0.41
			1.899	3.532	0.54	0.901	2.866	0.31			
			1.538	3.149	0.49	0.244	2.917	0.08			
			2.068	3.697	0.56	0.576	2.668	0.22			
