# Supplementary material for: Sequential and directional insulation by conserved CTCF sites underlies the Hox timer in stembryos
Source: Nat Genet. 2023 Jun 15;55(7):1164–75. doi: 10.1038/s41588-023-01426-7 (PMC10335938; doi:10.1038/s41588-023-01426-7)
Supplement: Supplementary file 2 — Reporting Summary [file 41588_2023_1426_MOESM2_ESM.pdf]

nature portfolio

Corresponding author(s): Denis Duboule

Last updated by author(s): Apr 27, 2023

Reporting Summary

Nature Portfolio wishes to improve the reproducibility of the work that we publish. This form provides structure for consistency and transparency in reporting. For further information on Nature Portfolio policies, see our Editorial Policies and the Editorial Policy Checklist.

Statistics

For all statistical analyses, confirm that the following items are present in the figure legend, table legend, main text, or Methods section.

n/a	Confirmed	
☐	☒	The exact sample size (*n*) for each experimental group/condition, given as a discrete number and unit of measurement
☐	☒	A statement on whether measurements were taken from distinct samples or whether the same sample was measured repeatedly
☐	☒	The statistical test(s) used AND whether they are one- or two-sided<br>*Only common tests should be described solely by name; describe more complex techniques in the Methods section.*
☒	☐	A description of all covariates tested
☐	☒	A description of any assumptions or corrections, such as tests of normality and adjustment for multiple comparisons
☐	☒	A full description of the statistical parameters including central tendency (e.g. means) or other basic estimates (e.g. regression coefficient) AND variation (e.g. standard deviation) or associated estimates of uncertainty (e.g. confidence intervals)
☐	☒	For null hypothesis testing, the test statistic (e.g. *F*, *t*, *r*) with confidence intervals, effect sizes, degrees of freedom and *P* value noted<br>*Give P values as exact values whenever suitable.*
☒	☐	For Bayesian analysis, information on the choice of priors and Markov chain Monte Carlo settings
☒	☐	For hierarchical and complex designs, identification of the appropriate level for tests and full reporting of outcomes
☒	☐	Estimates of effect sizes (e.g. Cohen's *d*, Pearson's *r*), indicating how they were calculated

Our web collection on statistics for biologists contains articles on many of the points above.

Software and code

Policy information about availability of computer code

Data collection

CRISPR guides for the production of the Del(sub-TAD1) mouse line were selected from the CRISPR/Cas9 -NGG Targets database (2019-10-13) in the UCSC Genome Browser.
Benchling platform was used to select sgRNA sequences used for mES cell lines production.
Stembryos images captured using the Olympus cellSens Standard software, version 2.1

Data analysis

All NGS analyses were performed on a local installation of galaxy.
All boxplots and barplots were plotted using Prism version 9. Associated p-values were evaluated by the same software.

Most of genomic tracks were plotted using pyGenomeTracks version 3.7 and modified with Illustrator 2022.
Heatmaps were plotted with R version 4.2.1

For RNA-seq analysis:
cutadapt version 1.16 was used to remove adapters
STAR version 2.7.7a was used to align on mm10
bamFilter version 2.4.1 was used to keep uniquely mapped reads
cufflinks version 2.2.1 was used to get FPKM values
A custom R script available on https://github.com/lldelisle/scriptsForRekaikEtAl2022 was used to normalize FPKM (R version 4.2.1).

For ChIP and ChIP-M analysis:
cutadapt version 1.16 was used to remove adapters
bowtie2 veresion 2.3.4.1 was used to align on mm10 or mutant genome

samtools version 1.8 was used to filter alignments
macs2 version 2.1.1.20160309 was used to compute coverage and call peaks
python version 3.9.12 was used to normalize coverages with a custom script available on https://github.com/lldelisle/scriptsForRekaikEtAl2022, compute cumulative coverage for figure 2A, compute linear extrapolation for Supplementary Movie 1, and to further normalize the H3K27ac and H3K27me3 datasets in Extended Data Figs. 8 and 10a.
multiBigwigSummary from deeptools version 3.0.0 was used to quantify
HOMER version 4.10 used for motif analysis of CDX2 ChIP-seq peaks

For Capture Hi-C and HiChIP analysis:
cutadapt version 1.16 was used to remove adapters
hicup version 0.6.1 was used to align and filter valid pairs
python version 3.8.5 with pysam 0.16.0.1 was used to convert bam file to juicebox format pairs file
cooler version 0.7.4 was used to generate matrices and balance them
Plot profile on ImageJ version 2.1.0/1.53c was used to generate histograms in Fig 3c.
python version 3.9.12 was used to generate virtual 4C with a script available on https://github.com/lldelisle/scriptsForRekaikEtAl2022 and to generate linear extrapolation for Supplementary Movie 2.

For single-cell RNA-seq:
Cell ranger version 6.0.0 was used to generate count matrix from fastq
R version 4.1.1 was used for the analysis.

For manuscripts utilizing custom algorithms or software that are central to the research but not yet described in published literature, software must be made available to editors and reviewers. We strongly encourage code deposition in a community repository (e.g. GitHub). See the Nature Portfolio guidelines for submitting code & software for further information.

Data

Policy information about availability of data

All manuscripts must include a data availability statement. This statement should provide the following information, where applicable:

- Accession codes, unique identifiers, or web links for publicly available datasets
- A description of any restrictions on data availability
- For clinical datasets or third party data, please ensure that the statement adheres to our policy

All raw and processed datasets are available in the Gene Expression Omnibus (GEO) repository under accession number GSE205783. All command lines and scripts to regenerate figures are available on https://github.com/lldelisle/scriptsForRekaikEtAl2022 (https://doi.org/10.5281/zenodo.7615150).

Human research participants

Policy information about studies involving human research participants and Sex and Gender in Research.

Reporting on sex and gender	not applicable
Population characteristics	not applicable
Recruitment	not applicable
Ethics oversight	not applicable

Note that full information on the approval of the study protocol must also be provided in the manuscript.

Field-specific reporting

Please select the one below that is the best fit for your research. If you are not sure, read the appropriate sections before making your selection.

☒ Life sciences ☐ Behavioural & social sciences ☐ Ecological, evolutionary & environmental sciences

For a reference copy of the document with all sections, see nature.com/documents/nr-reporting-summary-flat.pdf

Life sciences study design

All studies must disclose on these points even when the disclosure is negative.

Sample size

No statistical method was used to predetermine sample size. For RNA-seq experiments, biological duplicates were generated for each time-point and condition. ChIP and ChIP-M experiments were performed in biological duplicates when quantitative analysis was required. For time-courses, ChIP and ChIP-M were performed as single replicate for each time-point because we observed that the variation (enrichment, signal decrease) was maintained through the analyzed time-sequence and adding replicates will not provide more meaningful information to the final interpretation.
The same rationale was followed with CHiC time-courses, where single replicate was performed for each time-point and condition, however

	to increase the robustness of quantifications, triplicates were generated for wild-type 48h, 96h, 144h.
Data exclusions	Stembryos with poor elongation after 96h were excluded from the analysis. Some ChIP and ChIP-M libraries were excluded when a low fragment enrichment was detected after the analysis. The production of these datasets was repeated.
Replication	For RNA-seq and ChIP/ChIP-M, biological duplicate were performed. For each replicate, stembryos from 1 to 4 96-wells plates were pooled for each time-point and condition. Some ChIP/ChIP-M replicates were produced from different stembryos cultures.<br><br>For CHiC time-courses, pooled stembryos (1 to 4 plates) for each time-point were used to produce the library. In control at 48, 96 and 144h, 3 independent replicates were produced.<br><br>All attempts at replication were successful.
Randomization	No randomization were carried out in this study as experimental groups are based on genotypes and stembryos stages.
Blinding	Blinding was not relevant to this study because the nature of the data, based on genotypes and stembryos stages, can be independently identified.

Reporting for specific materials, systems and methods

We require information from authors about some types of materials, experimental systems and methods used in many studies. Here, indicate whether each material, system or method listed is relevant to your study. If you are not sure if a list item applies to your research, read the appropriate section before selecting a response.

Materials & experimental systems

n/a	Involved in the study
☐	☒ Antibodies
☐	☒ Eukaryotic cell lines
☒	☐ Palaeontology and archaeology
☐	☒ Animals and other organisms
☒	☐ Clinical data
☒	☐ Dual use research of concern

Methods

n/a	Involved in the study
☐	☒ ChIP-seq
☒	☐ Flow cytometry
☒	☐ MRI-based neuroimaging

Antibodies

Antibodies used	H3K27ac (ab4729, Abcam), polyclonal, lot# GR3216173-1<br>H3K27me3 (39155, Active Motif), polyclonal, lot# 31618020<br>PolII-Ser2p (04-1571, Millipore), clone 3E10, lot# 3589802<br>CTCF (61311, Active Motif), polyclonal, lot# 06520007<br>PolII (ab817, Abcam), clone 8WG16, lot# GR3254423-2<br>RAD21 (ab992, Abcam), polyclonal, lot# GR3310168-8<br>NIPBL (A301-779A, Bethyl Laboratories), polyclonal, lot# A301-779A<br>CDX2 (sc-393572, Santa Cruz), monoclonal, lot# C0821<br>Anti-DIG-AP (11093274910, Roche), polyclonal, lot# 32871921
Validation	All antibodies, according to their respective manufacturer, were stated to react to mouse and validated by Wester blot. In-house validation was performed by using ChIP or ChIP-M experiment on wild type stembryos. The signal intensity was assessed for each antibody at specific loci known to be positively enriched.

Eukaryotic cell lines

Policy information about cell lines and Sex and Gender in Research

Cell line source(s)	Del(sub-TAD1) and Del(CBS1-5) (Amândio et al. 2021) were derived from mouse blastocysts at the Mouse Clinical Institute (www.ics-mci.fr).<br>Wild type mES cells (EmbryoMax® 129/SVEV) were used to generate other mutant cell lines.
Authentication	PCR genotyping was performed to confirm the presence of the mutations in the cells.
Mycoplasma contamination	All cell lines tested negative for mycoplasma contamination
Commonly misidentified lines<br>(See ICLAC register)	No commonly misidentified lines were used in this study.

Animals and other research organisms

Policy information about studies involving animals; ARRIVE guidelines recommended for reporting animal research, and Sex and Gender in Research

Laboratory animals	10 weeks mice (mus musculus) were used for mES cell lines derivation. Animals were kept in a continuous back cross with BL6 X CBA F1 hybrids. Mice were housed in the University of Geneva Sciences III animalerie with light 07:00-19:00 in the summer, and 06:00-18:00 in winter with ambient temperatures maintained between 22-23°C and 45-55% humidity, the air is renewed 17-times per hour.
Wild animals	No wild animals were used in this study.
Reporting on sex	Sex of the animals was not considered in this study.
Field-collected samples	No field-collected samples were used in this study.
Ethics oversight	Experiments were performed in agreement with the Swiss Law on Animal Protection (LPA) under license numbers GE 81/14.

Note that full information on the approval of the study protocol must also be provided in the manuscript.

Data deposition

☒ Confirm that both raw and final processed data have been deposited in a public database such as GEO.

☒ Confirm that you have deposited or provided access to graph files (e.g. BED files) for the called peaks.

Data access links
May remain private before publication.

https://www.ncbi.nlm.nih.gov/geo/query/acc.cgi?acc=GSE205779

Files in database submission

Del(CBS1-2)_96h_CTCF.narrowPeak.gz
Del(CBS1-2)_96h_CTCF.bigwig
Del(CBS1-2)_96h_CTCF_R1.fastq.gz
Del(CBS1-2)_96h_CTCF_R2.fastq.gz
Del(CBS1-5)_96h_CTCF.narrowPeak.gz
Del(CBS1-5)_96h_CTCF.bigwig
Del(CBS1-5)_96h_CTCF_R1.fastq.gz
Del(CBS1-5)_96h_CTCF_R2.fastq.gz
Del(CBS1)_96h_CTCF.narrowPeak.gz
Del(CBS1)_96h_CTCF.bigwig
Del(CBS1)_96h_CTCF_R1.fastq.gz
Del(CBS1)_96h_CTCF_R2.fastq.gz
Del(CBS2)_96h_CTCF.narrowPeak.gz
Del(CBS2)_96h_CTCF.bigwig
Del(CBS2)_96h_CTCF_R1.fastq.gz
Del(CBS2)_96h_CTCF_R2.fastq.gz
Del(CBS4)_120h_CTCF.narrowPeak.gz
Del(CBS4)_120h_CTCF.bigwig
Del(CBS4)_120h_CTCF_R1.fastq.gz
Del(CBS4)_120h_CTCF_R2.fastq.gz
Ins(2xCBS-d4d8)_144h_CTCF.narrowPeak.gz
Ins(2xCBS-d4d8)_144h_CTCF.bigwig
Ins(2xCBS-d4d8)_144h_CTCF_R1.fastq.gz
Ins(2xCBS-d4d8)_144h_CTCF_R2.fastq.gz
wt_72h_CTCF.narrowPeak.gz
wt_72h_CTCF.bigwig
wt_72h_CTCF_R1.fastq.gz
wt_72h_CTCF_R2.fastq.gz
wt_168h_CTCF.narrowPeak.gz
wt_168h_CTCF.bigwig
wt_168h_CTCF_R1.fastq.gz
wt_168h_CTCF_R2.fastq.gz
wt_48h_H3K27ac_reptc.narrowPeak.gz
wt_48h_H3K27ac_reptc_Normalized.bigwig
wt_48h_H3K27ac_reptc_R1.fastq.gz
wt_48h_H3K27ac_reptc_R2.fastq.gz
wt_72h_H3K27ac_reptc.narrowPeak.gz
wt_72h_H3K27ac_reptc_Normalized.bigwig
wt_72h_H3K27ac_reptc_R1.fastq.gz
wt_72h_H3K27ac_reptc_R2.fastq.gz
wt_84h_H3K27ac_reptc.narrowPeak.gz

wt_84h_H3K27ac_reptc_Normalized.bigwig
wt_84h_H3K27ac_reptc_R1.fastq.gz
wt_84h_H3K27ac_reptc_R2.fastq.gz
wt_96h_H3K27ac_reptc.narrowPeak.gz
wt_96h_H3K27ac_reptc_Normalized.bigwig
wt_96h_H3K27ac_reptc_R1.fastq.gz
wt_96h_H3K27ac_reptc_R2.fastq.gz
wt_108h_H3K27ac_reptc.narrowPeak.gz
wt_108h_H3K27ac_reptc_Normalized.bigwig
wt_108h_H3K27ac_reptc_R1.fastq.gz
wt_108h_H3K27ac_reptc_R2.fastq.gz
wt_120h_H3K27ac_reptc.narrowPeak.gz
wt_120h_H3K27ac_reptc_Normalized.bigwig
wt_120h_H3K27ac_reptc_R1.fastq.gz
wt_120h_H3K27ac_reptc_R2.fastq.gz
wt_132h_H3K27ac_reptc.narrowPeak.gz
wt_132h_H3K27ac_reptc_Normalized.bigwig
wt_132h_H3K27ac_reptc_R1.fastq.gz
wt_132h_H3K27ac_reptc_R2.fastq.gz
wt_144h_H3K27ac_reptc.narrowPeak.gz
wt_144h_H3K27ac_reptc_Normalized.bigwig
wt_144h_H3K27ac_reptc_R1.fastq.gz
wt_144h_H3K27ac_reptc_R2.fastq.gz
wt_168h_H3K27ac_reptc.narrowPeak.gz
wt_168h_H3K27ac_reptc_Normalized.bigwig
wt_168h_H3K27ac_reptc_R1.fastq.gz
wt_168h_H3K27ac_reptc_R2.fastq.gz
Del(CBS1)_96h_H3K27ac_rep1.narrowPeak.gz
Del(CBS1)_96h_H3K27ac_rep1_Normalized.bigwig
Del(CBS1)_96h_H3K27ac_rep1_Normalized_HCN_96h.bedgraph
Del(CBS1)_96h_H3K27ac_rep1_R1.fastq.gz
Del(CBS1)_96h_H3K27ac_rep1_R2.fastq.gz
Del(CBS1)_96h_H3K27ac_rep2.narrowPeak.gz
Del(CBS1)_96h_H3K27ac_rep2_Normalized.bigwig
Del(CBS1)_96h_H3K27ac_rep2_Normalized_HCN_96h.bedgraph
Del(CBS1)_96h_H3K27ac_rep2_R1.fastq.gz
Del(CBS1)_96h_H3K27ac_rep2_R2.fastq.gz
Del(CBS1-2)_96h_H3K27ac_rep1.narrowPeak.gz
Del(CBS1-2)_96h_H3K27ac_rep1_Normalized.bigwig
Del(CBS1-2)_96h_H3K27ac_rep1_Normalized_HCN_96h.bedgraph.gz
Del(CBS1-2)_96h_H3K27ac_rep1_R1.fastq.gz
Del(CBS1-2)_96h_H3K27ac_rep1_R2.fastq.gz
Del(CBS1-2)_96h_H3K27ac_rep2.narrowPeak.gz
Del(CBS1-2)_96h_H3K27ac_rep2_Normalized.bigwig
Del(CBS1-2)_96h_H3K27ac_rep2_Normalized_HCN_96h.bedgraph.gz
Del(CBS1-2)_96h_H3K27ac_rep2_R1.fastq.gz
Del(CBS1-2)_96h_H3K27ac_rep2_R2.fastq.gz
wt_96h_H3K27ac_rep1.narrowPeak.gz
wt_96h_H3K27ac_rep1_Normalized.bigwig
wt_96h_H3K27ac_rep1_Normalized_HCN_96h.bedgraph
wt_96h_H3K27ac_rep1_R1.fastq.gz
wt_96h_H3K27ac_rep1_R2.fastq.gz
wt_96h_H3K27ac_rep2.narrowPeak.gz
wt_96h_H3K27ac_rep2_Normalized.bigwig
wt_96h_H3K27ac_rep2_Normalized_HCN_96h.bedgraph
wt_96h_H3K27ac_rep2_R1.fastq.gz
wt_96h_H3K27ac_rep2_R2.fastq.gz
Del(CBS1)_120h_H3K27ac_rep1.narrowPeak.gz
Del(CBS1)_120h_H3K27ac_rep1_Normalized.bigwig
Del(CBS1)_120h_H3K27ac_rep1_Normalized_HCN_120h.bedgraph
Del(CBS1)_120h_H3K27ac_rep1_R1.fastq.gz
Del(CBS1)_120h_H3K27ac_rep1_R2.fastq.gz
Del(CBS1)_120h_H3K27ac_rep2.narrowPeak.gz
Del(CBS1)_120h_H3K27ac_rep2_Normalized.bigwig
Del(CBS1)_120h_H3K27ac_rep2_Normalized_HCN_120h.bedgraph
Del(CBS1)_120h_H3K27ac_rep2_R1.fastq.gz
Del(CBS1)_120h_H3K27ac_rep2_R2.fastq.gz
Del(CBS1-2)_120h_H3K27ac_rep1.narrowPeak.gz
Del(CBS1-2)_120h_H3K27ac_rep1_Normalized.bigwig
Del(CBS1-2)_120h_H3K27ac_rep1_Normalized_HCN_120h.bedgraph.gz
Del(CBS1-2)_120h_H3K27ac_rep1_R1.fastq.gz
Del(CBS1-2)_120h_H3K27ac_rep1_R2.fastq.gz
Del(CBS1-2)_120h_H3K27ac_rep2.narrowPeak.gz
Del(CBS1-2)_120h_H3K27ac_rep2_Normalized.bigwig
Del(CBS1-2)_120h_H3K27ac_rep2_Normalized_HCN_120h.bedgraph.gz
Del(CBS1-2)_120h_H3K27ac_rep2_R1.fastq.gz

Del(CBS1-2)_120h_H3K27ac_rep2_R2.fastq.gz
wt_120h_H3K27ac_rep1.narrowPeak.gz
wt_120h_H3K27ac_rep1_Normalized.bigwig
wt_120h_H3K27ac_rep1_Normalized_HCN_120h.bedgraph
wt_120h_H3K27ac_rep1_R1.fastq.gz
wt_120h_H3K27ac_rep1_R2.fastq.gz
wt_120h_H3K27ac_rep2.narrowPeak.gz
wt_120h_H3K27ac_rep2_Normalized.bigwig
wt_120h_H3K27ac_rep2_Normalized_HCN_120h.bedgraph
wt_120h_H3K27ac_rep2_R1.fastq.gz
wt_120h_H3K27ac_rep2_R2.fastq.gz
wt_72h_H3K27me3_reptc.narrowPeak.gz
wt_72h_H3K27me3_reptc_Normalized.bigwig
wt_72h_H3K27me3_reptc_R1.fastq.gz
wt_72h_H3K27me3_reptc_R2.fastq.gz
wt_96h_H3K27me3_reptc.narrowPeak.gz
wt_96h_H3K27me3_reptc_Normalized.bigwig
wt_96h_H3K27me3_reptc_R1.fastq.gz
wt_96h_H3K27me3_reptc_R2.fastq.gz
wt_120h_H3K27me3_reptc.narrowPeak.gz
wt_120h_H3K27me3_reptc_Normalized.bigwig
wt_120h_H3K27me3_reptc_R1.fastq.gz
wt_120h_H3K27me3_reptc_R2.fastq.gz
wt_144h_H3K27me3_reptc.narrowPeak.gz
wt_144h_H3K27me3_reptc_Normalized.bigwig
wt_144h_H3K27me3_reptc_R1.fastq.gz
wt_144h_H3K27me3_reptc_R2.fastq.gz
Del(CBS1-5)_96h_H3K27me3_rep1.narrowPeak.gz
Del(CBS1-5)_96h_H3K27me3_rep1_Normalized.bigwig
Del(CBS1-5)_96h_H3K27me3_rep1_Normalized_HCN_96h.bedgraph
Del(CBS1-5)_96h_H3K27me3_rep1_R1.fastq.gz
Del(CBS1-5)_96h_H3K27me3_rep1_R2.fastq.gz
Del(CBS1-5)_96h_H3K27me3_rep2.narrowPeak.gz
Del(CBS1-5)_96h_H3K27me3_rep2_Normalized.bigwig
Del(CBS1-5)_96h_H3K27me3_rep2_Normalized_HCN_96h.bedgraph
Del(CBS1-5)_96h_H3K27me3_rep2_R1.fastq.gz
Del(CBS1-5)_96h_H3K27me3_rep2_R2.fastq.gz
wt_96h_H3K27me3_rep1.narrowPeak.gz
wt_96h_H3K27me3_rep1_Normalized.bigwig
wt_96h_H3K27me3_rep1_Normalized_HCN_96h.bedgraph
wt_96h_H3K27me3_rep1_R1.fastq.gz
wt_96h_H3K27me3_rep1_R2.fastq.gz
wt_96h_H3K27me3_rep2.narrowPeak.gz
wt_96h_H3K27me3_rep2_Normalized.bigwig
wt_96h_H3K27me3_rep2_Normalized_HCN_96h.bedgraph
wt_96h_H3K27me3_rep2_R1.fastq.gz
wt_96h_H3K27me3_rep2_R2.fastq.gz
wt_72h_NIPBL.narrowPeak.gz
wt_72h_NIPBL_Normalized.bigwig
wt_72h_NIPBL_R1.fastq.gz
wt_72h_NIPBL_R2.fastq.gz
wt_84h_NIPBL.narrowPeak.gz
wt_84h_NIPBL_Normalized.bigwig
wt_84h_NIPBL_R1.fastq.gz
wt_84h_NIPBL_R2.fastq.gz
wt_96h_NIPBL.narrowPeak.gz
wt_96h_NIPBL_Normalized.bigwig
wt_96h_NIPBL_R1.fastq.gz
wt_96h_NIPBL_R2.fastq.gz
wt_108h_NIPBL.narrowPeak.gz
wt_108h_NIPBL_Normalized.bigwig
wt_108h_NIPBL_R1.fastq.gz
wt_108h_NIPBL_R2.fastq.gz
wt_120h_NIPBL.narrowPeak.gz
wt_120h_NIPBL_Normalized.bigwig
wt_120h_NIPBL_R1.fastq.gz
wt_120h_NIPBL_R2.fastq.gz
wt_132h_NIPBL.narrowPeak.gz
wt_132h_NIPBL_Normalized.bigwig
wt_132h_NIPBL_R1.fastq.gz
wt_132h_NIPBL_R2.fastq.gz
wt_144h_NIPBL.narrowPeak.gz
wt_144h_NIPBL_Normalized.bigwig
wt_144h_NIPBL_R1.fastq.gz
wt_144h_NIPBL_R2.fastq.gz
wt_168h_NIPBL.narrowPeak.gz

wt_168h_NIPBL_Normalized.bigwig
wt_168h_NIPBL_R1.fastq.gz
wt_168h_NIPBL_R2.fastq.gz
wt_72h_PolII.narrowPeak.gz
wt_72h_PolII_Normalized.bigwig
wt_72h_PolII_R1.fastq.gz
wt_72h_PolII_R2.fastq.gz
wt_84h_PolII.narrowPeak.gz
wt_84h_PolII_Normalized.bigwig
wt_84h_PolII_R1.fastq.gz
wt_84h_PolII_R2.fastq.gz
wt_96h_PolII.narrowPeak.gz
wt_96h_PolII_Normalized.bigwig
wt_96h_PolII_R1.fastq.gz
wt_96h_PolII_R2.fastq.gz
wt_108h_PolII.narrowPeak.gz
wt_108h_PolII_Normalized.bigwig
wt_108h_PolII_R1.fastq.gz
wt_108h_PolII_R2.fastq.gz
wt_120h_PolII.narrowPeak.gz
wt_120h_PolII_Normalized.bigwig
wt_120h_PolII_R1.fastq.gz
wt_120h_PolII_R2.fastq.gz
wt_132h_PolII.narrowPeak.gz
wt_132h_PolII_Normalized.bigwig
wt_132h_PolII_R1.fastq.gz
wt_132h_PolII_R2.fastq.gz
wt_144h_PolII.narrowPeak.gz
wt_144h_PolII_Normalized.bigwig
wt_144h_PolII_R1.fastq.gz
wt_144h_PolII_R2.fastq.gz
wt_168h_PolII.narrowPeak.gz
wt_168h_PolII_Normalized.bigwig
wt_168h_PolII_R1.fastq.gz
wt_168h_PolII_R2.fastq.gz
wt_72h_pSer2PolII.narrowPeak.gz
wt_72h_pSer2PolII_Normalized.bigwig
wt_72h_pSer2PolII_R1.fastq.gz
wt_72h_pSer2PolII_R2.fastq.gz
wt_84h_pSer2PolII.narrowPeak.gz
wt_84h_pSer2PolII_Normalized.bigwig
wt_84h_pSer2PolII_R1.fastq.gz
wt_84h_pSer2PolII_R2.fastq.gz
wt_96h_pSer2PolII.narrowPeak.gz
wt_96h_pSer2PolII_Normalized.bigwig
wt_96h_pSer2PolII_R1.fastq.gz
wt_96h_pSer2PolII_R2.fastq.gz
wt_120h_pSer2PolII.narrowPeak.gz
wt_120h_pSer2PolII_Normalized.bigwig
wt_120h_pSer2PolII_R1.fastq.gz
wt_120h_pSer2PolII_R2.fastq.gz
wt_144h_pSer2PolII.narrowPeak.gz
wt_144h_pSer2PolII_Normalized.bigwig
wt_144h_pSer2PolII_rep1_R1.fastq.gz
wt_144h_pSer2PolII_rep1_R2.fastq.gz
wt_144h_pSer2PolII_rep2_R1.fastq.gz
wt_144h_pSer2PolII_rep2_R2.fastq.gz
wt_144h_pSer2PolII_rep3_R1.fastq.gz
wt_144h_pSer2PolII_rep3_R2.fastq.gz
Del(CBS1-5)_120h_RAD21.narrowPeak.gz
Del(CBS1-5)_120h_RAD21.bigwig
Del(CBS1-5)_120h_RAD21_R1.fastq.gz
Del(CBS1-5)_120h_RAD21_R2.fastq.gz
wt_48h_RAD21_rep1.narrowPeak.gz
wt_48h_RAD21_rep1_Normalized.bigwig
wt_48h_RAD21_rep1_R1.fastq.gz
wt_48h_RAD21_rep1_R2.fastq.gz
wt_72h_RAD21_rep1.narrowPeak.gz
wt_72h_RAD21_rep1_Normalized.bigwig
wt_72h_RAD21_R1.fastq.gz
wt_72h_RAD21_R2.fastq.gz
wt_84h_RAD21_rep1.narrowPeak.gz
wt_84h_RAD21_rep1_Normalized.bigwig
wt_84h_RAD21_R1.fastq.gz
wt_84h_RAD21_R2.fastq.gz
wt_96h_RAD21_rep1.narrowPeak.gz

Methodology

Replicates

ChIP and ChIP-M experiments were performed on biological duplicates when comparing wild type to mutants stembryos. Each replicate is composed of pool of stembryos. Wild type time-courses were performed on a singleton which was composed of pool of stembryos. CHiC and HiChIP experiments were also performed as singleton from a pool of stembryos.

Sequencing depth

Del(CBS1-2)_96h_CTCF: sequenced to 45.8939 million pairs of 42 and 42bp
Del(CBS1-5)_96h_CTCF: sequenced to 45.2406 million pairs of 43 and 43bp
Del(CBS1)_96h_CTCF: sequenced to 41.3872 million pairs of 43 and 43bp
Del(CBS2)_96h_CTCF: sequenced to 44.8301 million pairs of 42 and 42bp
Del(CBS4)_120h_CTCF: sequenced to 41.6316 million pairs of 42 and 42bp
Ins(2xCBS-d4d8)_144h_CTCF: sequenced to 38.4857 million pairs of 42 and 42bp
wt_72h_CTCF: sequenced to 74.1102 million pairs of 38 and 37bp
wt_168h_CTCF: sequenced to 48.0939 million pairs of 38 and 38bp
wt_48h_H3K27ac_reptc: sequenced to 46.8558 million pairs of 43 and 43bp
wt_72h_H3K27ac_reptc: sequenced to 38.2312 million pairs of 38 and 38bp
wt_84h_H3K27ac_reptc: sequenced to 35.2842 million pairs of 38 and 38bp
wt_96h_H3K27ac_reptc: sequenced to 41.0535 million pairs of 38 and 38bp
wt_108h_H3K27ac_reptc: sequenced to 39.4378 million pairs of 38 and 38bp
wt_120h_H3K27ac_reptc: sequenced to 35.5746 million pairs of 38 and 38bp
wt_132h_H3K27ac_reptc: sequenced to 42.7177 million pairs of 38 and 38bp
wt_144h_H3K27ac_reptc: sequenced to 39.3981 million pairs of 38 and 38bp
wt_168h_H3K27ac_reptc: sequenced to 36.9394 million pairs of 38 and 38bp
Del(CBS1)_96h_H3K27ac_rep1: sequenced to 46.6971 million pairs of 43 and 43bp
Del(CBS1)_96h_H3K27ac_rep2: sequenced to 42.0132 million pairs of 43 and 43bp
Del(CBS1-2)_96h_H3K27ac_rep1: sequenced to 43.2117 million pairs of 42 and 42bp
Del(CBS1-2)_96h_H3K27ac_rep2: sequenced to 39.8453 million pairs of 42 and 42bp
wt_96h_H3K27ac_rep1: sequenced to 42.6674 million pairs of 43 and 43bp
wt_96h_H3K27ac_rep2: sequenced to 37.9469 million pairs of 43 and 43bp
Del(CBS1)_120h_H3K27ac_rep1: sequenced to 45.3467 million pairs of 43 and 43bp
Del(CBS1)_120h_H3K27ac_rep2: sequenced to 44.2978 million pairs of 43 and 43bp
Del(CBS1-2)_120h_H3K27ac_rep1: sequenced to 49.1289 million pairs of 42 and 42bp
Del(CBS1-2)_120h_H3K27ac_rep2: sequenced to 44.5184 million pairs of 42 and 42bp
wt_120h_H3K27ac_rep1: sequenced to 41.3815 million pairs of 43 and 43bp
wt_120h_H3K27ac_rep2: sequenced to 36.1414 million pairs of 43 and 43bp
wt_72h_H3K27me3_reptc: sequenced to 33.5514 million pairs of 43 and 43bp
wt_96h_H3K27me3_reptc: sequenced to 28.9914 million pairs of 43 and 43bp
wt_120h_H3K27me3_reptc: sequenced to 33.7924 million pairs of 43 and 43bp
wt_144h_H3K27me3_reptc: sequenced to 24.9301 million pairs of 42 and 42bp
Del(CBS1-5)_96h_H3K27me3_rep1: sequenced to 46.3274 million pairs of 43 and 43bp
Del(CBS1-5)_96h_H3K27me3_rep2: sequenced to 49.7485 million pairs of 43 and 43bp
wt_96h_H3K27me3_rep1: sequenced to 48.9729 million pairs of 43 and 43bp
wt_96h_H3K27me3_rep2: sequenced to 47.1578 million pairs of 43 and 43bp
wt_72h_NIPBL: sequenced to 26.8563 million pairs of 38 and 38bp
wt_84h_NIPBL: sequenced to 45.1668 million pairs of 38 and 38bp
wt_96h_NIPBL: sequenced to 51.8706 million pairs of 38 and 37bp
wt_108h_NIPBL: sequenced to 64.1666 million pairs of 38 and 38bp
wt_120h_NIPBL: sequenced to 41.4113 million pairs of 38 and 38bp
wt_132h_NIPBL: sequenced to 39.1029 million pairs of 38 and 38bp
wt_144h_NIPBL: sequenced to 71.4503 million pairs of 38 and 38bp
wt_168h_NIPBL: sequenced to 79.6795 million pairs of 38 and 38bp
wt_72h_PolII: sequenced to 41.8611 million pairs of 38 and 37bp
wt_84h_PolII: sequenced to 43.0498 million pairs of 38 and 37bp
wt_96h_PolII: sequenced to 19.3932 million pairs of 38 and 37bp
wt_108h_PolII: sequenced to 37.8099 million pairs of 38 and 37bp
wt_120h_PolII: sequenced to 25.8376 million pairs of 38 and 37bp
wt_132h_PolII: sequenced to 42.1423 million pairs of 38 and 37bp
wt_144h_PolII: sequenced to 54.5459 million pairs of 38 and 37bp
wt_168h_PolII: sequenced to 58.0452 million pairs of 38 and 37bp
wt_72h_pSer2PolII: sequenced to 58.1197 million pairs of 43 and 43bp
wt_84h_pSer2PolII: sequenced to 46.0012 million pairs of 42 and 42bp
wt_96h_pSer2PolII: sequenced to 48.4638 million pairs of 43 and 43bp
wt_120h_pSer2PolII: sequenced to 59.1914 million pairs of 43 and 43bp
wt_144h_pSer2PolII: sequenced to 122.727 million pairs of 42 and 42bp
Del(CBS1-5)_120h_RAD21: sequenced to 57.6942 million pairs of 42 and 42bp
wt_48h_RAD21_rep1: sequenced to 50.5769 million pairs of 42 and 42bp
wt_72h_RAD21: sequenced to 92.0336 million pairs of 38 and 37bp
wt_84h_RAD21: sequenced to 41.1625 million pairs of 38 and 37bp
wt_96h_RAD21: sequenced to 47.8457 million pairs of 38 and 37bp
wt_108h_RAD21: sequenced to 42.1339 million pairs of 38 and 37bp
wt_132h_RAD21: sequenced to 49.416 million pairs of 38 and 37bp
wt_144h_RAD21: sequenced to 54.2442 million pairs of 38 and 37bp
wt_168h_RAD21: sequenced to 45.8563 million pairs of 38 and 38bp
wt_72h_RAD21_rep2: sequenced to 52.506 million pairs of 42 and 42bp
wt_84h_RAD21_rep2: sequenced to 52.178 million pairs of 42 and 42bp

wt_96h_RAD21_rep1_Normalized.bigwig
wt_96h_RAD21_R1.fastq.gz
wt_96h_RAD21_R2.fastq.gz
wt_108h_RAD21_rep1.narrowPeak.gz
wt_108h_RAD21_rep1_Normalized.bigwig
wt_108h_RAD21_R1.fastq.gz
wt_108h_RAD21_R2.fastq.gz
wt_132h_RAD21_rep1.narrowPeak.gz
wt_132h_RAD21_rep1_Normalized.bigwig
wt_132h_RAD21_R1.fastq.gz
wt_132h_RAD21_R2.fastq.gz
wt_144h_RAD21_rep1.narrowPeak.gz
wt_144h_RAD21_rep1_Normalized.bigwig
wt_144h_RAD21_R1.fastq.gz
wt_144h_RAD21_R2.fastq.gz
wt_168h_RAD21_rep1.narrowPeak.gz
wt_168h_RAD21_rep1_Normalized.bigwig
wt_168h_RAD21_R1.fastq.gz
wt_168h_RAD21_R2.fastq.gz
wt_72h_RAD21_rep2.narrowPeak.gz
wt_72h_RAD21_rep2_Normalized.bigwig
wt_72h_RAD21_rep2_R1.fastq.gz
wt_72h_RAD21_rep2_R2.fastq.gz
wt_84h_RAD21_rep2.narrowPeak.gz
wt_84h_RAD21_rep2_Normalized.bigwig
wt_84h_RAD21_rep2_R1.fastq.gz
wt_84h_RAD21_rep2_R2.fastq.gz
wt_96h_RAD21_rep2.narrowPeak.gz
wt_96h_RAD21_rep2_Normalized.bigwig
wt_96h_RAD21_rep2_R1.fastq.gz
wt_96h_RAD21_rep2_R2.fastq.gz
wt_108h_RAD21_rep2.narrowPeak.gz
wt_108h_RAD21_rep2_Normalized.bigwig
wt_108h_RAD21_rep2_R1.fastq.gz
wt_108h_RAD21_rep2_R2.fastq.gz
wt_132h_RAD21_rep2.narrowPeak.gz
wt_132h_RAD21_rep2_Normalized.bigwig
wt_132h_RAD21_rep2_R1.fastq.gz
wt_132h_RAD21_rep2_R2.fastq.gz
wt_144h_RAD21_rep2.narrowPeak.gz
wt_144h_RAD21_rep2_Normalized.bigwig
wt_144h_RAD21_rep2_R1.fastq.gz
wt_144h_RAD21_rep2_R2.fastq.gz
wt_96h_RAD21_rep3.narrowPeak.gz
wt_96h_RAD21_rep3_Normalized.bigwig
wt_96h_RAD21_rep3_R1.fastq.gz
wt_96h_RAD21_rep3_R2.fastq.gz
wt_144h_RAD21_rep3.narrowPeak.gz
wt_144h_RAD21_rep3_Normalized.bigwig
wt_144h_RAD21_rep3_R1.fastq.gz
wt_144h_RAD21_rep3_R2.fastq.gz
Del(CBS1)_96h_RAD21_rep1.narrowPeak.gz
Del(CBS1)_96h_RAD21_rep1_Normalized.bigwig
Del(CBS1)_96h_RAD21_rep1_R1.fastq.gz
Del(CBS1)_96h_RAD21_rep1_R2.fastq.gz
Del(CBS1)_96h_RAD21_rep2.narrowPeak.gz
Del(CBS1)_96h_RAD21_rep2_Normalized.bigwig
Del(CBS1)_96h_RAD21_rep2_R1.fastq.gz
Del(CBS1)_96h_RAD21_rep2_R2.fastq.gz
wt_72h_CDX2.narrowPeak.gz
wt_72h_CDX2_Normalized.bigwig
wt_72h_CDX2_R1.fastq.gz
wt_72h_CDX2_R2.fastq.gz
wt_96h_CDX2.narrowPeak.gz
wt_96h_CDX2_Normalized.bigwig
wt_96h_CDX2_R1.fastq.gz
wt_96h_CDX2_R2.fastq.gz
wt_120h_CDX2.narrowPeak.gz
wt_120h_CDX2_Normalized.bigwig
wt_120h_CDX2_R1.fastq.gz
wt_120h_CDX2_R2.fastq.gz

Genome browser session
(e.g. UCSC)

NA

wt_96h_RAD21_rep2: sequenced to 51.3222 million pairs of 42 and 42bp
wt_108h_RAD21_rep2: sequenced to 53.6701 million pairs of 42 and 42bp
wt_120h_CDX2: sequenced to 41.9977 million pairs of 43 and 43bp
wt_132h_RAD21_rep2: sequenced to 83.6686 million pairs of 42 and 42bp
wt_144h_RAD21_rep2: sequenced to 51.0171 million pairs of 42 and 42bp
wt_96h_RAD21_rep3: sequenced to 51.4115 million pairs of 42 and 42bp
wt_144h_RAD21_rep3: sequenced to 63.3835 million pairs of 42 and 42bp
wt_72h_CDX2: sequenced to 39.9208 million pairs of 43 and 43bp
wt_96h_CDX2: sequenced to 39.4182 million pairs of 43 and 43bp
wt_120h_CDX2: sequenced to 41.9977 million pairs of 43 and 43bp

Antibodies

H3K27ac (ab4729, Abcam), polyclonal, lot# GR3216173-1
H3K27me3 (39155, Active Motif), polyclonal, lot# 31618020
PolII-Ser2p (04-1571, Millipore), clone 3E10, lot# 3589802
CTCF (61311, Active Motif), polyclonal, lot# 06520007
PolII (ab817, Abcam), clone 8WG16, lot# GR3254423-2
RAD21 (ab992, Abcam), polyclonal, lot# GR3310168-8
NIPBL (A301-779A, Bethyl Laboratories), polyclonal, lot# A301-779A
CDX2 (sc-393572, Santa Cruz), monoclonal, lot# C0821

Peak calling parameters

```
# cutadapt version :1.16
cutadapt -j ${GALAXY_SLOTS:-1} -a 'Please use: For R1: - For Nextera: CTGTCTCTTATACACATCTCCGAGCCCACGAGAC - For TrueSeq:
GATCGGAAGAGCACACGTCTGAACTCCAGTCAC'='GATCGGAAGAGCACACGTCTGAACTCCAGTCAC' -A 'Please use: For R2: - For Nextera:
CTGTCTCTTATACACATCTGACGCTGCCGACGA - For TruSeq:
GATCGGAAGAGCGTCGTGTAGGGAAAGAGTGTAGATCTCGGTGGTCGCCGTATCATT'='GATCGGAAGAGCGTCGTGTAGGGAAAGAGTGTAGA
TCTCGGTGGTCGCCGTATCATT' --output='out1.gz' --paired-output='out2.gz' 'sample_R1.fq.gz' 'sample_R2.fq.gz' > report.txt

# bowtie2 version 2.3.4.1
ln -f -s 'cutadapt of sample_R1.fastqsanger.gz' input_f.fastq.gz
 ln -f -s 'cutadapt of sample_R2.fastqsanger.gz' input_r.fastq.gz
  bowtie2  -p ${GALAXY_SLOTS:-4}  -x '/data/galaxy/galaxy/var/tool-data/mm10_UCSC/bowtie2_index/mm10_UCSC/mm10_UCSC'
-1 'input_f.fastq.gz' -2 'input_r.fastq.gz' 2> 'mapping stats of cutadapt of sample .txt' | samtools sort -@${GALAXY_SLOTS:-2} -O bam -
o 'mapping of cutadapt of sample.bam'

# samtools version 1.8
ln -s 'mapping of cutadapt of sample.bam' input.bam
 ln -s '/data/galaxy/data/_metadata_files/012/metadata_12490.dat' input.bai
 samtools view -o 'filtered bam of mapping of cutadapt of sample.bam' -h   -b  -q 30 -f 0x2 input.bam 2>&1

# macs2 version 2.1.1.20160309:
macs2 callpeak --name 'MACS2' -t 'filtered bam of mapping of cutadapt of sample.bam' --format BAMPE --gsize '1870000000' --call-
summits --bdg
```

Data quality

To assess quality of ChIP 2 ranges are given, first the number of peaks, then the number of peaks with fold-change above 5 (7th field of narrowPeak).
CTCF: 25-60k of peaks and 15-45k with fold-change above 5
H3K27ac: 24-65k of peaks and 6-11k with fold-change above 5
H3K27me3: 7-11k of peaks and 600-2000 with fold-change above 5
NIPBL: 5-40k of peaks and 800-6000 with fold-change above 5
PolII: 12-20k of peaks and 8-12k with fold-change above 5
pSer2PolII: 4-24k of peaks and 400-6000 with fold-change above 5
RAD21: 32-70k of peaks and 16-35k with fold-change above 5
CDX2: 200-5000 peaks and 100-2000 with fold-change above 5

Software

cutadapt version 1.16 was used to remove adapters
bowtie2 version 2.3.4.1 was used to align on mm10 or mutant genome
samtools version 1.8 was used to filter alignments
macs2 version 2.1.1.20160309 was used to compute coverage and call peaks
python version 3.9.12 was used to normalize coverages with a custom script available on https://github.com/lldelisle/scriptsForRekaikEtAl2022, compute cumulative coverage for figure 2A, compute linear extrapolation for Supplementary Movie 1, and to further normalize the H3K27ac and H3K27me3 datasets in Extended Data Figs. 8 and 10a.
multiBigwigSummary from deeptools version 3.0.0 was used to quantify
R version 4.2.1 was used to generate heatmaps
HOMER version 4.10 was used to generate motif analysis of CDX2 ChIP-seq
